# The *Escherichia coli alkA* Gene Is Activated to Alleviate Mutagenesis by an Oxidized Deoxynucleoside

**DOI:** 10.3389/fmicb.2020.00263

**Published:** 2020-02-25

**Authors:** Kristin Grøsvik, Almaz Nigatu Tesfahun, Izaskun Muruzábal-Lecumberri, Gyri Teien Haugland, Ingar Leiros, Peter Ruoff, Jan Terje Kvaløy, Ingeborg Knævelsrud, Hilde Ånensen, Marina Alexeeva, Kousuke Sato, Akira Matsuda, Ingrun Alseth, Arne Klungland, Svein Bjelland

**Affiliations:** ^1^Department of Chemistry, Bioscience and Environmental Technology, Centre for Organelle Research, Faculty of Science and Technology, University of Stavanger, Stavanger, Norway; ^2^Department of Biological Sciences, University of Bergen, Bergen, Norway; ^3^The Norwegian Structural Biology Centre, Department of Chemistry, UiT – The Arctic University of Norway, Tromsø, Norway; ^4^Department of Mathematics and Physics, Faculty of Science and Technology, University of Stavanger, Stavanger, Norway; ^5^Faculty of Pharmaceutical Sciences, Health Sciences University of Hokkaido, Tobetsu, Japan; ^6^Faculty of Pharmaceutical Sciences, Hokkaido University, Sapporo, Japan; ^7^Department of Microbiology, Oslo University Hospital, Oslo, Norway; ^8^Department of Molecular Medicine, Institute of Basic Medical Sciences, University of Oslo, Oslo, Norway

**Keywords:** 5-formyluracil, oxidized base repair, base substitution, AlkA, DNA glycosylase

## Abstract

The cellular methyl donor *S*-adenosylmethionine (SAM) and other endo/exogenous agents methylate DNA bases non-enzymatically into products interfering with replication and transcription. An important product is 3-methyladenine (m^3^A), which in *Escherichia coli* is removed by m^3^A-DNA glycosylase I (Tag) and II (AlkA). The *tag* gene is constitutively expressed, while *alkA* is induced by sub-lethal concentrations of methylating agents. We previously found that AlkA exhibits activity for the reactive oxygen-induced thymine (T) lesion 5-formyluracil (fU) *in vitro*. Here, we provide evidence for AlkA involvement in the repair of oxidized bases by showing that the adenine (A) ⋅ T → guanine (G) ⋅ cytosine (C) mutation rate increased 10-fold in *E. coli* wild-type and *alkA*^–^ cells exposed to 0.1 mM 5-formyl-2′-deoxyuridine (fdU) compared to a wild-type specific reduction of the mutation rate at 0.2 mM fdU, which correlated with *alkA* gene induction. G⋅C → A⋅T alleviation occurred without *alkA* induction (at 0.1 mM fdU), correlating with a much higher AlkA efficiency for fU opposite to G than for that to A. The common keto form of fU is the AlkA substrate. Mispairing with G by ionized fU is favored by its exclusion from the AlkA active site.

## Introduction

Reactive oxygen species (ROS) are formed as by-products in the electron transport chain of bacterial plasma membranes, mitochondria, chloroplasts, and some other eukaryotic organelles. Thus, oxidative damage to DNA and its precursors is substantial in all aerobic cells. ROS are also formed by photosensitization reactions involving both ultraviolet and visible light. The most reactive ROS is the hydroxyl radical (⋅OH), which is formed from hydrogen peroxide (H_2_O_2_) and Fe^2+^ in the Fenton reaction, and by ionizing radiation. Singlet oxygen is another important reactive, but non-radical, oxygen form [for a review see Halliwell and Gutteridge (1989)].

Several different nucleic acid base modifications are formed by ROS (Cadet et al., 1997). Many of these, including ring-saturated and ring-fragmented thymine (T) residues, are mainly cytotoxic lesions that contribute slightly to mutation induction (Bjelland and Seeberg, 2003). In contrast, guanine (G) oxidized at the 8-position (8-oxo-7,8-dihydroguanine; oxoG) is a principal mutagenic ROS-induced lesion (Lindahl, 1993; Freudenthal et al., 2015). The pyrimidine oxidation products 5-hydroxycytosine, 5-hydroxyuracil, and uracil glycol also cause mispairing and contribute to mutation induction (Feig et al., 1994; Purmal et al., 1994; Kreutzer and Essigmann, 1998). In *Escherichia coli*, oxidized bases are primarily removed from DNA by three DNA glycosylases: formamidopyrimidine-DNA glycosylase (Fpg), endonuclease III (Nth), and endonuclease VIII (Nei) (Zharkov et al., 2003; Boiteux et al., 2017; Lee and Wallace, 2017). How cells repair and tolerate DNA lesions has considerable impact on human diseases, including cancer and neurodegeneration (Berquist and Wilson, 2012; Dizdaroglu, 2015).

Almost 30 years ago, 5-formyluracil (fU) was identified as an abundant oxidized form of thymine in DNA, generated in yields similar to oxoG (Kasai et al., 1990; Douki et al., 1996). Later, a potential fU mutagenic or cytotoxic effect was suggested from the observation that fU is removed efficiently from DNA by 3-methyladenine (m^3^A)-DNA glycosylase II (AlkA) in *E. coli* (Bjelland et al., 1994) and by DNA glycosylase present in mammalian and human cell-free extracts (Bjelland et al., 1995; Zhang et al., 1995). The *K*_*M*_ and *V*_*max*_ values for AlkA release of fU are similar to m^3^A (Bjelland et al., 1994). AlkA is the *E. coli* homolog to the extensively characterized mammalian methylpurine-DNA glycosylase (MPG) (Labahn et al., 1996; Yamagata et al., 1996; Hollis et al., 2000; O’Brien and Ellenberger, 2004; Sedgwick, 2004), which exhibits no activity for fU (Bjelland et al., 1994). DNA glycosylases initiate the base excision repair (BER) pathway, which is predominantly completed by reinsertion of a single nucleotide (nt) via 5′-acting apurinic/apyrimidinic (AP) endonuclease (Xth and Nfo proteins in *E. coli*) (Doetsch and Cunningham, 1990), DNA deoxyribose-5′-phosphate lyase, DNA polymerase, and DNA ligase activity (Lindahl and Wood, 1999; Friedberg et al., 2006; Zharkov, 2008). Several *in vitro* and *in vivo* studies have demonstrated that fU generated in DNA or incorporated into DNA from the nucleotide precursor pool induces several base substitutions, except the G ⋅ cytosine (C) → C⋅G transversion (Yoshida et al., 1997; Zhang et al., 1997; Fujikawa et al., 1998; Terato et al., 1999; Ånensen et al., 2001). Oxidized bases or nucleosides may be taken up from the environment, anabolized, and incorporated into DNA. Importantly, this metabolic pathway for base damage generation has been mostly neglected.

Methyl transfer reactions are involved in numerous cellular processes. *S*-adenosylmethionine (SAM) is the most common methyl donor used by methyltransferases. However, in addition to its function as a methyltransferase co-substrate, SAM and other endogenous and exogenous agents non-enzymatically methylate normal DNA bases into products that interfere with replication and transcription (Sedgwick, 2004; Shrivastav et al., 2010; Soll et al., 2017). An important product is m^3^A, which is removed by m^3^A-DNA glycosylase I (Tag) (Sakumi et al., 1986; Bjelland and Seeberg, 1987) and II (AlkA) in *E. coli* (Nakabeppu et al., 1984). While the *tag* gene is constitutively expressed, *alkA* is induced by sub-lethal methylating agent concentrations (Evensen and Seeberg, 1982; Karran et al., 1982). Because of this, the discovery that AlkA efficiently removes fU from DNA *in vitro* was unexpected (Bjelland et al., 1994). Although this was confirmed by other studies (Masaoka et al., 1999; Terato et al., 1999), no supporting *in vivo* evidence exists for AlkA involvement in counteracting the consequences of ROS-induced DNA damage.

Here, we present the first *in vivo* evidence that AlkA is involved in the processing of fU lesions in DNA. When 5-formyl-2′-deoxyuridine (fdU) is added to exponentially growing *E. coli* cells to incorporate fU into DNA, repair-proficient wild-type and repair-deficient *alkA* gene-lacking cells respond differently, indicating that AlkA is a key factor in determining the base substitution induced by fdU. In addition, we demonstrate that fdU increases *alkA* gene expression.

## Materials and Methods

### Materials

5-Formyl-2′-deoxyuridine was prepared as described (Ono et al., 1994). Rifampicin was obtained from Sigma (Cat. No. R-3501).

### Bacterial Cells

Three *E. coli* K-12 derivatives were investigated: AB1157 (wild-type) (Boyce and Howard-Flanders, 1964); MS23 [*alkA*^–^; AB1157 transduced by P1(*his^+^ alkA1^–^*)] (Yamamoto et al., 1978); and BW528 (*xth^–^ nfo^–^*) (Gu et al., 1993). Frozen glycerol cultures were stored at −80°C.

### Mutagenesis

Mutagenesis was performed as previously described (Cupples and Miller, 1989), with all growth carried out at 37°C. Cells were transferred from freeze culture to 5 mL LB (lysogeny-broth) medium, grown overnight, and spread on LB agar plates for further growth and short-term storage at 4°C. All cells in a mutagenesis culture were derived from a single colony picked from the LB agar plate and grown overnight in 2 mL A medium [A buffer (see below) containing 1 mM MgSO_4_, 0.2% (w/v) glucose, 0.04 mg/mL L-amino acids (Thr, Arg, Pro, Leu, His), and vitamin B_1_ (5 μg/mL)] (contamination controls without cells added). The number of cells after overnight culture was determined from the OD_600_. Overnight cultures (10 μL) were diluted in A buffer [K_2_HPO_4_ (10.5 g/L), KH_2_PO_4_ (4.5 g/L), (NH_4_)_2_SO_4_ (1 g/L), and C_6_H_5_Na_3_O_7_ × 2H_2_O (0.5 g/L)] to (usually) 100,000–200,000 cells/mL. Then, 9000–45,000 cells were added to 2 mL A medium. The cultures grew for 2 h to allow for adaptation of cells and increase cell number. fdU addition (controls without fdU determined the spontaneous mutagenesis rate) defined the start of the mutagenesis experiment/culture (2 mL). Cells cultured with fdU were grown for 20–50 h. The rif^R^ mutants and viable cells were selected on minimal agar plates containing rifampicin (150 μg/mL).

### Mutation Rate

The experimental procedures to determine the mutation rate μ follow recommendations described elsewhere (Rosche and Foster, 2000) for the *p*_0_ method (Luria and Delbrück, 1943), which is frequently used to estimate low mutation rates, such as deoxynucleoside analog (like fdU)-induced mutations. Thus, mutagenesis cultures grown to a similar final cell concentration (*N*_t_) of 1.5–1.8 × 10^9^ per 2 mL were selected from each experimental group (i.e. a certain *E. coli* strain/mutant growing in the presence of a defined fdU concentration). Then, μ was calculated according to the *p*_0_ method, where *p*_0_ (the mutant-free culture proportion) was between 0.1 and 0.3 for all groups. From each culture, we usually plated 100 μL on each rifampicin-containing plate (six replicates per culture), which resulted in a *z* (fraction of a culture plated) value of 0.3. Only cultures with *z*-values identical or very close to 0.3 were included (see the section “Mutagenesis Experiments” in the Supplementary Material). The estimator *m*_obs_ (observed number of mutations per culture) was calculated from the observed *p*_0_ value and ranged from 1.1 to 2.3, resulting in *m*_act_ (actual number of mutations per culture) values from 2.1 to 4.5, following calculation by equation [18] in the cited article (Rosche and Foster, 2000; see Supplementary Table S2A).

### DNA Amplification and Sequencing

The rif^R^ colonies were grown at 37°C in 2 mL LB medium containing 150 μL/mL rifampicin (only one mutant was characterized from each mutagenesis culture). Enriched chromosomal DNA was prepared for polymerase chain reaction (PCR) by heating 5 μL bacterial culture in 100 μL sterile water at 100°C for 5 min, followed by cooling on ice. Cultures were centrifuged and the supernatant collected. The *rpoB* rif^R^ region was amplified by PCR using the forward primer 5′-GCCAAGCCGATTTCC-3′ (F-1021) and the reverse primer 5′-GTATTCGTTAGTCTG-3′ (R-1022) (0.2 pmol/μL each) using a KOD Hot Start DNA Polymerase kit (VWR) or GoTaq HotStart Polymerase (Promega) in a 50 μL reaction volume. The PCR products (10–80 ng DNA) were purified using illustra MicroSpin S-400 HR columns (GE Healthcare) or NucleoSpin Gel and PCR Clean-up (Macherey-Nagel). DNA sequencing (using F-1021 as primer) was performed by the Genome Analysis Centre, Norwich, United Kingdom and GATC Biotech, Cologne, Germany (using an Applied Biosystems 3730xl DNA Analyzer).

### *alkA* Wild-Type Gene Cloning and Expression and AlkA Protein Purification

The *alkA* gene was amplified from DNA isolated from *E. coli* K-12 (AB1157) by PCR using the following primers: forward, 5′-GCCGTCATATGTATACCCTGAACTGGCAGCC-3′, *T*_*m*_ 67.26°C; reverse, 5′-CCGCTCGAG**TCA**TCATGCTTCGTCT GGTTGCCA-3′, *T*_*m*_ 71.46°C (*T*_*m*_ was determined by the Thermo Scientific *T*_*m*_ calculator web tool. The first stop codon is outlined in bold). The PCR product was inserted into the pET21a vector (NEB), using the restriction enzymes *Nde*I and *Xho*I (recognition sites underlined in the forward and reverse primer sequences, respectively) and T4 DNA ligase (all three enzymes from NEB). Plasmid was transformed into One shot chemical competent TOP10 *E. coli* cells (Invitrogen). Expressed plasmid was purified from three positive colonies identified by PCR screening using Dynazyme and Finnzymes (ThermoFisher Scientific). Correct insert sequences were confirmed by sequencing both DNA strands at the DNA Sequencing Facility at the High Technology Centre in Bergen, Norway. After testing the *E. coli* cells BL-21-CodonPlus (DE3)-RIL (Stratagene), BL21(DE3) (Invitrogen), and EB106DE3 for *alkA* gene overexpression and AlkA production, CodonPlus and BL21 cells were found to be superior. *alkA* was overexpressed in BL21 cells, since they produced the highest amount of soluble AlkA. The bacteria were grown in 1-l ZYP-5052 medium according to Studier’s auto-induction method (Studier, 2005) and incubated at 37°C for 24 h. After harvesting by centrifugation (4 g wet weight, stored at −20°C), the cells were resuspended in 0.1 M Tris–HCl pH 8.5, 1 mM ethylenediaminetetraacetic acid (EDTA), 10 mM β-mercaptoethanol, and 20% (v/v) glycerol (1 g cells per 10 mL), disrupted by sonication and the cell debris was removed by centrifugation. Soluble proteins were precipitated with ammonium sulfate (40–70% fraction). AlkA protein was purified using a previously described procedure (Bjelland et al., 1994), with slight modifications. The ammonium sulfate precipitate was dissolved in 5 mL 0.1 M Tris–HCl pH 8.5, 1 mM EDTA, 10 mM β-mercaptoethanol, and 20% (v/v) glycerol and filtered with a 26/60 Superdex 200 prep grade column (Cat. No. 17-1071-01, GE Healthcare) in 330 mL of the same buffer. AlkA eluted after 248 mL, and six 3-mL fractions were collected and pooled. The protein was concentrated by centrifugation using a 20 mL Vivaspin concentrator with a 3000 MWCO PES membrane (Sartorius Stedim Biotech). The elution buffer was exchanged to 50 mM Mes pH 6.0, 1 mM EDTA, 10 mM β-mercaptoethanol, and 20% (v/v) glycerol with PD-10 desalting columns (GE Healthcare). Cation exchange chromatography was carried out on a 5 mL Bio-Scale Mini Macro-Prep High S column (Bio-Rad) equilibrated with the same buffer. Proteins were eluted with a 0–1.0 M NaCl gradient. AlkA started to elute at 0.24 M NaCl, and 1-mL fractions were collected (total volume: 21 mL). The protein was concentrated using the same concentrator and membrane as above, and the buffer exchanged to 0.2 M Tris–HCl pH 7.27, 1 mM EDTA, 1 mM dithiothreitol (DTT), and 20% (v/v) glycerol with PD Spin Trap G25 columns (GE Healthcare, Cat. No. 28-9180-04). Protein fractions from each step during the purification procedure were analyzed by sodium dodecyl sulfate (SDS)-polyacrylamide gel electrophoresis (PAGE) (Supplementary Figure S3). Protein concentration was measured using Bio-Rad Protein Assay (BioRad, Cat. No. 500-0006).

### Incision of 5-Formyluracil-Containing DNA

Polydeoxynucleotides containing fdU monophosphate (fdUMP) at a specific site were synthesized as described (Ono et al., 1994), and fluorescently labeled with Cy3 at the 5′-end (Sigma and Eurofins). The labeled strand [Cy3] 5′-GGCGAATTCGAGCTCGGTACCCGGGGATCCTCTAGAG TfUGACCTGCAGGCATGCAAGCTTGAGT-3′ (64 nt) was annealed to equimolar amounts of a complementary strand with A or G opposite fU (called fU⋅A and fU⋅G substrate, respectively) by heating at 90°C for 3 min followed by cooling at 37°C for 15 min and to room temperature for 30 min. Increasing AlkA amounts were incubated with 11 nM fU⋅A or fU⋅G substrates in 70 mM MOPS [3-(*N*-morpholino)propanesulfonic acid] pH 7.0, 1 mM EDTA, 1 mM DTT, and 5% (v/v) glycerol (reaction buffer) at 37°C for 1 h in a final volume of 50 μL. Reactions were terminated by the addition of 20 mM EDTA, 0.5% (w/v) SDS, and 190 μg/mL proteinase K. DNA was precipitated with ethanol and resuspended in 10 μL water. The abasic sites formed by AlkA were incised by 0.1 M NaOH treatment at 90°C for 10 min. Samples were mixed with 10 μL loading solution containing 80% (v/v) formamide, 1 mM EDTA, and 0.05% (w/v) Blue Dextran, and incubated at 95°C for 5 min to denature DNA. After cooling on ice, 10 samples were subjected to denaturing PAGE [20% (w/v) polyacrylamide gels with 7 M urea]. Experimental data were fitted to Michaelis–Menten kinetics by non-linear regression using KaleidaGraph (Synergy Software).

### pH Dependence of the Excision of fU and m^3^A From DNA by AlkA

The m^3^A-DNA glycosylase activity was determined as a function of pH as previously described (Bjelland and Seeberg, 1987). This also applies to the determination of fU-DNA glycosylase activity using substrate and enzyme conditions as described elsewhere (Bjelland et al., 1994). To prepare [^3^H]methylated DNA, calf thymus DNA (Sigma, code D3664) was treated with [^3^H]*N-*methyl-*N*′-nitrosourea (MNU; 18.4 Ci/mmol, GE Healthcare), as previously described (Alseth et al., 2005). *E. coli* [*methyl*-^3^H]thymine-labeled DNA, which accumulates [^3^H]fU residues upon storage (Bjelland et al., 1995), was provided by New England Nuclear (Cat. No. NET-561). Essentially, [^3^H]MNU-treated [2000 disintegrations per min (dpm)] and aged [*methyl*-^3^H]thymine-labeled DNA (10,000 dpm; 4000 dpm [^3^H]fU) were incubated with AlkA (0.084–0.168 and 1.969 pmol, respectively) (Bjelland et al., 1994, 1995) in a modified universal buffer (Johnson and Lindsey, 1939; Bjelland and Seeberg, 1987) at 37°C for 10 min, followed by DNA precipitation with ethanol. Supernatant radioactivity was measured in a Tri-Carb^TM^ liquid scintillation analyzer (Model 1900 TR) from Packard.

### fU and m^3^A Docking in the AlkA Active Site

AlkA served as the receptor in automated docking simulations using AutoDock software (Morris et al., 1998), based on the AlkA crystal structure in complex with 10 nt double-stranded DNA containing the modified abasic nt 1-azaribose (Hollis et al., 2000). A single-stranded trinucleotide with the 1-azaribose part substituted by fdU was modified into the docking ligand to reduce the molecular complexity and computing power. Nevertheless, super-positioning the docked trinucleotide back onto the DNA fragment in the crystal structure of the DNA–protein complex (Hollis et al., 2000) resulted in similar conformations, indicating that the DNA–protein interactions were adequately mimicked in the automatic docking trials. Since m^3^A is the preferred AlkA substrate (Lindahl, 1993), m^3^A-containing trinucleotide was included as a positive control. To allow for some degree of ligand reorientation during docking, four fU and three m^3^A freely rotational bonds were selected.

### *alkA* and *ada* Gene Expression Analysis

RT-qPCR was used to determine the relative *alkA* and *ada* expression in wild-type *E. coli* (AB1157) 1, 3, 6, and 24 h after adding 0.1 or 0.2 mM fdU to the growth culture. A bacterial growth culture was prepared by adding 500 μL cells from an overnight culture (OD_600_ ∼1.5) to 10 mL LB medium. After 2 h incubation to OD_600_ ∼0.5, 3 mL growth culture was treated with 0.1 or 0.2 mM fdU (control samples without treatment). Then, the samples were incubated for 1, 3, 6, and 24 h. All incubations were performed at 37°C with shaking. Before RNA isolation, 1.5 mL culture (1 × 10^8^ cells) from each sample was mixed with 500 μL RNAprotect Cell Reagent (Qiagen) for RNA stabilization. Total RNA was extracted using a TRIzol Max Bacterial RNA Isolation Kit (Ambion Life Technologies). Genomic DNA contamination was removed by DNase I treatment using a RNA purification kit (Zymo Research), followed by resuspension of RNA pellets in 50 μL DNase/RNase-free water. Next, synthesis of double-stranded cDNA and integrated removal of genomic DNA was conducted using a QuantiTect reverse transcription reagent kit (Qiagen). To normalize the target gene expression level for each sample, 1 μg total RNA was used for cDNA synthesis in a 20 μL final reaction volume. cDNA was synthesized at 42°C for 15 min and 95°C for 3 min. Diluted cDNA (fivefold) was used as a template for amplifications using 10 μM target primer mix (Primerdesign) and SYBR green master mix (Qiagen) in a 20 μL reaction mixture. Test samples without reverse transcriptase and without template were used to test for genomic DNA and other technical contaminations. PCR assays were performed with a LightCycler 96 instrument (Roche, Germany) with the following conditions: 95°C for 2 min, followed by 45 cycles of 95°C for 15 s, 60°C for 60 s, and 65°C for 30 s. All data shown resulted from three independent replicates. Real-time PCR runs were measured in duplicate. Relative target gene expression was normalized to reference gene (*tag*) expression.

### Statistical Analysis

Continuous data are reported as averages and standard errors. Categorical data are reported as counts and percentages. Chi-square or Fisher’s exact tests, as appropriate, were used for testing differences in base substitution distributions between different mutant types and different fdU levels. *P*-values ≤ 0.05 were considered statistically significant. The statistical analyses were done in R version 3.4.3 (R Core Team, 2017). Confidence intervals for mutation rates were calculated as described (Rosche and Foster, 2000).

## Results

### AlkA Influences fdU Mutagenicity in *E. coli*

When fdU is converted to fdUMP *in vivo*, the latter inhibits thymidylate synthase causing cellular toxicity (Park et al., 1980; Ahmad et al., 1998; Masaoka et al., 1999; Klungland et al., 2001). However, mutagenesis experiments using an initial cell number (*N*_0_) of 9000 (as determined by OD_600_) indicated no significant growth inhibition by 0.1 or 0.2 mM fdU (Supplementary Figure S1 and Supplementary Table S1A) showing moderate toxicity in both the wild-type and the repair-deficient cells. Further, these results agree with the notion that fU in DNA is a mutagenic lesion (Bjelland and Seeberg, 2003).

Our goal was to investigate whether there is any connection between fdU mutagenesis and BER, focusing on AlkA as the most active DNA glycosylase that removes fU from DNA (Bjelland et al., 1994; Bjelland and Seeberg, 2003). We systematically determined the mutation rate (μ) following the recommendations presented elsewhere (Rosche and Foster, 2000). Accordingly, wild-type (*alkA*^+^) was compared to the *alkA*^–^ and *xth^–^ nfo^–^* strains. The latter is deficient in exonuclease III (Xth) and endonuclease IV (Nfo), and thus should have increased unrepaired AP sites following fU excision. The spontaneous mutation rates in the *alkA*^–^ and *xth^–^ nfo^–^* cells were identical, and only marginally higher than in wild-type cells (Table 1A), indicating efficient back-up repair systems for AlkA, Xth, and Nfo under normal physiological conditions. Supplementing the culture medium with 0.1 mM fdU roughly doubled the mutation rate in the wild-type and *alkA*^–^ cells, demonstrating a moderate mutagenicity both in the presence and absence of AlkA. Interestingly, 0.1 mM fdU caused a smaller increase (1.6-fold) in mutation rate in the *xth^–^ nfo^–^* cells than in the wild-type and *alkA*^–^ cells. This largely rules out the possibility that fdU mutagenesis occurs through trans-lesion bypass of AP sites caused by increased excision of fU from DNA at this concentration, rather than via the fdU lesion itself. Increasing fdU to 0.2 mM unexpectedly caused a noticeable decrease in the wild-type mutation rate, while the *alkA*^–^ cells remained unaffected. This indicates that AlkA counteracts fdU mutation induction. In *xth^–^ nfo^–^* cells, the mutation rate remained the same when the concentration increased from 0.1 to 0.2 mM fdU (Table 1A).

**TABLE 1:** Mutation rates for rif^R^ in exponentially growing wild-type, *alkA*^–^, and *xth^–^ nfo^–^ E. coli* in the presence of 0–0.2 mM fdU.

**(A)**						
	**Wild-type**		***alkA*^–^**		***xth^–^ nfo^–^***	
	
**fdU (mM)**	**Mutation rate (×10^–9^)**					
		**Fold change**		**Fold change**		**Fold change**

0	1.3 (1.1; 1.5)	1	1.4 (1.2; 1.6)	1	1.4 (1.1; 1.7)	1
0.1	2.4 (1.8; 3.0)	1.8	2.9 (2.2; 3.6)	2.1	2.3 (1.7; 2.9)	1.6
0.2	1.7 (1.3; 2.2)	1.3	2.7 (2.0; 3.4)	1.9	2.4 (1.8; 3.0)	1.7

(**B**)						

	**Wild-type**		***alkA*^–^**			
	
**fdU (mM)**	**Mutation rate (×10^–9^)**					
		**Fold change**		**Fold change**		

0	2.4 (1.5; 3.4)	1	1.3 (0.6; 2.2)	1		
0.1	5.6 (3.4; 8.2)	2.3	4.7 (2.8; 7.0)	3.6		
0.2	2.2 (1.3; 3.3)	0.92	3.1 (1.9; 4.4)	2.4		

*A, primary culture selection (*N*_t_ = 1.5–1.8 ± 0.5 × 10^9^ cells); B, secondary culture selection (*N*_t_ = 0.6–0.7 ± 0.2 × 10^9^ cells). The 95% confidence interval is indicated in parentheses. Number of experiments (*C*), median of viable cells (×10^9^) per 2 ml mutagenesis culture (*N*_t_), and other parameters are presented in Supplementary Table S2.*

Another selection of independent cultures with 0.6 ± 0.2 (median = 0.6) × 10^9^ cells per 2 mL (Supplementary Tables S1B, S2B, and the section “Mutagenesis Experiments, Secondary Selection” in the Supplementary Material), although resulting in less accurate mutation rate values due to the lower number of cultures (Table 1B), largely confirmed the above results (Table 1A). Importantly, the increased mutation rate at 0.1 mM fdU was larger for both wild type and *alkA*^–^ cells. When 0.2 mM fdU was added, the rate decreased to the spontaneous level in wild-type cells, but not *alkA*^–^ cells (Table 1B).

In conclusion, adding 0.1 mM fdU to the growth medium doubles the mutation rate in both wild-type and *alkA*^–^ cells, while 0.2 mM fdU causes a lower mutation rate in wild-type cells, but not *alkA*^–^ cells, suggesting that AlkA alleviates fdU-mediated mutagenesis.

### fdU Alters the Distribution of Base Substitutions Independent of AlkA

**FIGURE 1:**
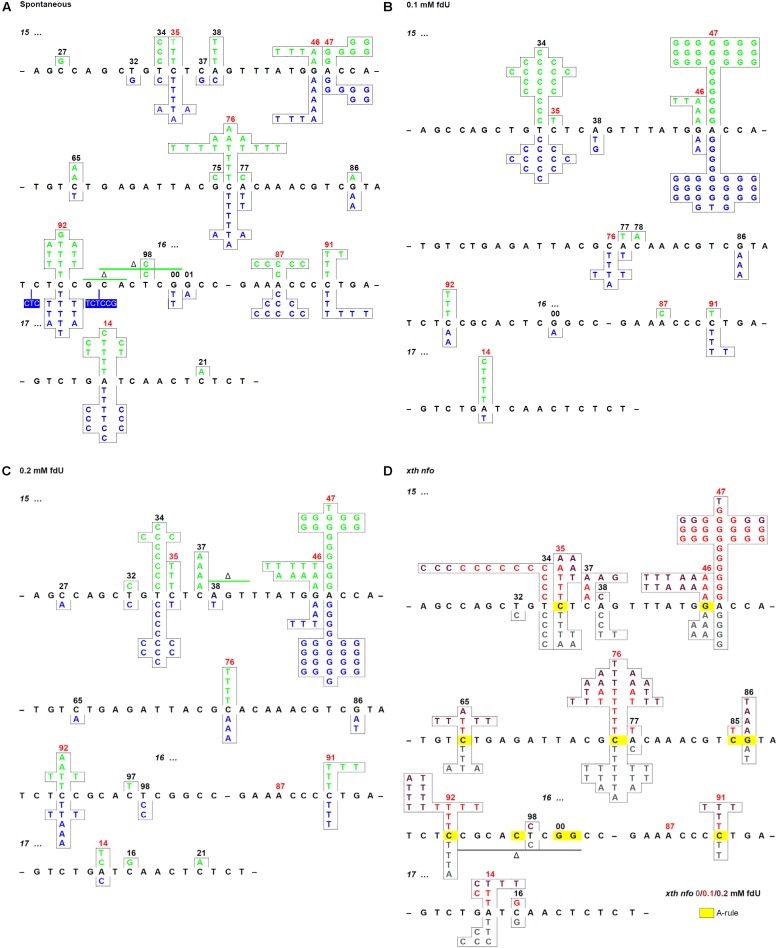
Mutation spectra of the *E. coli* repair deficient and proficient cells with and without fdU in the growth culture. **(A)** Spontaneous mutations in the *rpoB* gene rif^R^ region of wild-type (*alkA*^+^; green) and *alkA*^–^ (blue) cells. **(B)** Mutations in the rif^R^ region of wild-type (green) and *alkA*^–^ (blue) cells grown with 0.1 mM fdU, **(C)** 0.2 mM fdU. **(D)** *xth^–^ nfo^–^* cells grown with 0 (gray), 0.1 (red), and 0.2 mM (dark red) fdU. Spots where the A-rule should apply are marked in yellow. In omitted regions (indicated by a short black line), no mutation was detected. Spontaneous mutation hot spots are indicated by red numbers. See Table 2A for the number of mutations presented.

We next investigated whether AlkA deficiency influences the mutation spectrum at different fdU concentrations. Consequently, we determined the mutation types induced by fdU in *E. coli* by sequencing the rifampicin resistant (rif^R^) region of at least 60 mutants exposed to the same mutagen concentration (Table 2A and Figure 1). The results verified that most rif^R^ mutations in all investigated cell types are base substitutions, with very few insertions and deletions (indels) (Table 2A and Supplementary Figure S2A; see the sections “Base Substitutions” and “Indels” in the Supplementary Material for a more detailed description of the mutations). Therefore, neither indels nor the uncharacterized fraction outside the sequenced rif^R^ region are included in the statistical analysis.

**TABLE 2:** Base substitutions [**A**, in percent; **B**, their mutation rates (μ)] among *E. coli* rif^R^ mutants induced by 0.1 and 0.2 mM fdU.

**(A)**															
	**Wild-type**			***alkA*^–^**			***xth^–^ nfo^–^***								
**fdU (mM)**	**0**	**0.1**	**0.2**	**0**	**0.1**	**0.2**	**0**	**0.1**	**0.2**						

AT → CG	12 (10)	2.9 (2)	1.8 (1)	22 (19)	0	1.4 (1)	10 (7)	1.4 (1)	2.7 (2)						
GC → AT	35 (30)	12 (8)	35 (22)	38 (33)	23 (15)	15 (11)	46 (31)	33 (23)	45 (33)						
GC → CG	3.5 (3)	0	1.8 (1)	1.2 (1)	0	0	1.5 (1)	1.4 (1)	1.4 (1)						
GC → TA	14 (12)	4.4 (3)	19 (12)	15 (13)	4.6 (3)	17 (12)	13 (9)	7.1 (5)	26 (19)						
AT → TA	10 (9)	7.4 (5)	3.5 (2)	7.0 (6)	6.1 (4)	1.4 (1)	6.0 (4)	4.3 (3)	5.4 (4)						
AT → GC	14 (12)	66 (45)	40 (26)	9.3 (8)	62 (41)	55 (39)	15 (10)	49 (34)	11 (8)						
BS	88 (76)	93 (63)	93 (64)	93 (80)	95 (63)	90 (64)	93 (62)	96 (67)	91 (67)						
Indels	2.3 (2)	0	1.5 (1)	2.3 (2)	0	0	1.5 (1)	0	0						
Unknown*	9.3 (8)	7.4 (5)	5.8 (4)	4.7 (4)	4.6 (3)	9.9 (7)	6.0 (4)	4.3 (3)	9.5 (7)						
Total	100 (86)	100 (68)	100 (69)	100 (86)	100 (66)	100 (71)	100 (67)	100 (70)	100 (74)						

**(B)**															

	**Wild-type**					***alkA*^–^**					***xth^–^ nfo^–^***				
**μ (×10^–9^)**	**μ**		**μ_0.1_/μ_0_**	**μ**	**μ_0.2_/μ_0_**	**μ**		**μ_0.1_/μ_0_**	**μ**	**μ_0.2_/μ_0_**	**μ**		**μ_0.1_/μ_0_**	**μ**	**μ_0.2_/μ_0_**
**fdU (mM)**	**0**	**0.1**	**Ratio**	**0.2**	**Ratio**	**0**	**0.1**	**Ratio**	**0.2**	**Ratio**	**0**	**0.1**	**Ratio**	**0.2**	**Ratio**

AT → CG	0.148	0.0706	0.48	0.0248	0.17	0.307	0	0	0.0379	0.12	0.141	0.0327	0.23	0.0643	0.46
GC → AT	0.443	0.282	0.64	0.545	1.2	0.533	0.657	1.2	0.417	0.78	0.625	0.752	1.2	1.06	1.7
GC → CG	0.0443	0	0	0.0248	0.56	0.0161	0	0	0	0	0.0201	0.0327	1.6	0.0321	1.6
GC → TA	0.177	0.106	0.60	0.297	1.7	0.210	0.131	0.62	0.455	2.2	0.181	0.164	0.91	0.611	3.4
AT → TA	0.133	0.176	1.3	0.0496	0.37	0.0970	0.175	1.8	0.0379	0.39	0.0806	0.0982	1.2	0.129	1.6
AT → GC	0.177	1.59	9.0	0.644	3.6	0.129	1.80	14	1.48	11	0.202	1.11	5.5	0.257	1.3
BS	1.12	2.22	2.0	1.59	1.4	1.29	2.76	2.1	2.42	1.9	1.25	2.19	1.8	2.15	1.7
Indels	0.0296	0	0	0.0248	0.84	0.0324	0	0	0	0	0.0201	0	0	0	0
Unknown*	0.118	0.176	1.5	0.0992	0.84	0.0646	0.131	2.0	0.265	4.1	0.0806	0.0982	1.2	0.225	2.8
Total	1.27	2.40	1.9	1.71	1.3	1.39	2.89	2.1	2.69	1.9	1.35	2.29	1.7	2.38	1.8

Number of mutants is indicated in parenthesis. BS, base substitutions; *mutated outside the sequenced rif^R^ region.

The results showed no significant difference in distribution of spontaneous base substitutions between wild-type and *alkA*^–^ cells (*p* = 0.40), or between wild-type and the *xth^–^ nfo^–^* cells (*p* = 0.76). Thus, a similar baseline was used to score the effect of fdU in wild-type and mutant cell lines. As expected, the base substitutions in wild-type cells changed significantly after exposure to 0.1 mM (*p* = 0.000001) and 0.2 mM fdU (*p* = 0.0015; Table 2A), confirming fdU mutagenicity (Fujikawa et al., 1998). Similar results were obtained for the *alkA*^–^ cells at 0.1 mM (*p* = 0.000001) and 0.2 mM fdU (*p* = 0.000001), and for the *xth^–^ nfo^–^* cells at 0.1 mM fdU (*p* = 0.0005), when compared to the spontaneous distribution. Unexpectedly, 0.2 mM fdU in the *xth^–^ nfo^–^* cells caused no significant change in the base substitution distribution (*p* = 0.27; Table 2A).

Although there was a strong effect on the base substitutions distribution induced by fdU, no significant difference in distribution was observed between the wild-type and *alkA*^–^ cells when treated with 0.1 mM (*p* = 0.36) or 0.2 mM (*p* = 0.14) fdU, which implies that the AlkA effect on mutagenesis is quantitative rather than qualitative.

### The A⋅T → G⋅C Transition Is Primarily Induced by fdU and Is Alleviated by AlkA

Most mutations in the wild-type and *alkA*^–^ cells following fdU exposure were adenine (A)⋅ T *to* G⋅C transitions. While representing around 10–15% of the spontaneous mutations in the wild-type and *alkA*^–^ cells, this transition increased to 66% (*p* = 0.000001) and 62% (*p* = 0.000001) at 0.1 mM, and to 40% (*p* = 0.0012) and 55% (*p* = 0.000001) at 0.2 mM, respectively (Table 2A). This result corroborates our previous report showing that A⋅T → G⋅C is the most frequent base substitution induced by fdU in wild-type *E. coli* (Ånensen et al., 2001). Although this difference in substitution abundance between the wild-type and *alkA*^–^ cells was not significant in controls (*p* = 0.34) and at 0.1 mM fdU (*p* = 0.57), 0.2 mM fdU caused significantly different substitution levels (*p* = 0.034). The fraction of A⋅T → G⋅C in wild-type cells decreased to almost the half from 0.1 to 0.2 mM fdU, while being quite similar at the two fdU concentrations in *alkA*^–^ cells (Table 2A).

**FIGURE 2:**
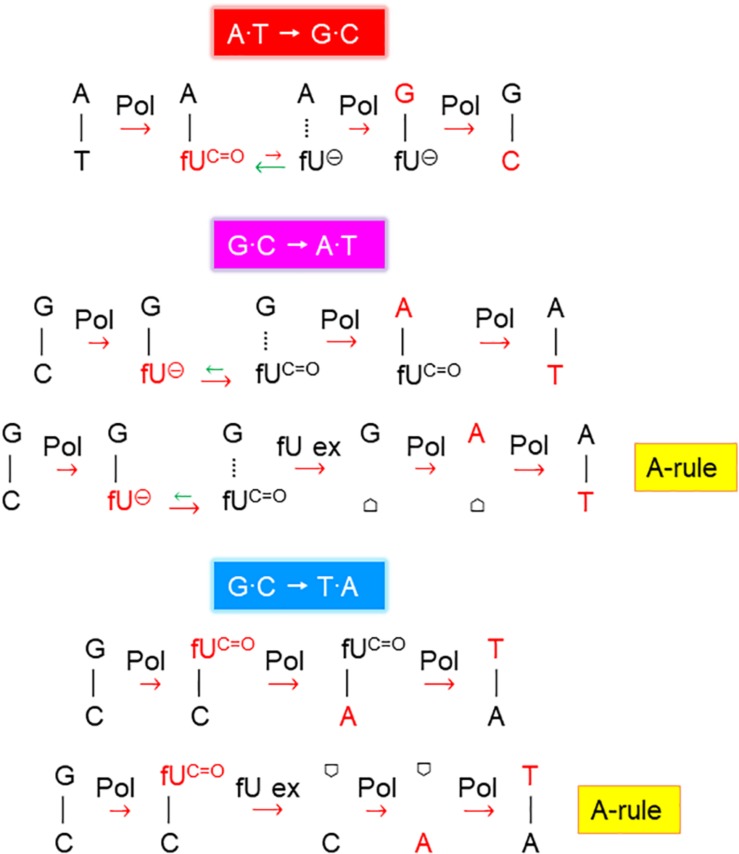
Proposed fdU mutagenesis mechanisms. Schemes suggesting the major molecular events inducing A⋅T → G⋅C, G⋅C → A⋅T, and G⋅C → T⋅A mutations by fdU. Pol, DNA polymerase; fU^C=O^, keto form of fU, which pairs with A (and less efficiently with C); fU^⊖^, ionized form of fU, which pairs with G; 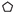, AP site/deoxyribose; template and newly inserted bases are presented in black and red, respectively; the major processes or events alleviating (green arrows) and contributing (red arrows) to mutagenesis are indicated (short, weak effect; long, stronger effect).

In the unexposed *xth^–^ nfo^–^* cells, the A⋅T → G⋅C fraction was equal to wild-type cells (*p* = 1). The fraction increased to 49% (*p* = 0.00003) with 0.1 mM fdU, which was significantly lower than in wild-type cells (*p* = 0.020; Table 2A). This supports the notion that A⋅T → G⋅C mutations cannot arise via AP site formation, according to the A-rule, because of fU excision (Figure 2). This conclusion was supported by 0.2 mM fdU treatment, where the A⋅T → G⋅C fraction in the *xth^–^ nfo^–^* cells (11%) was similar to that without fdU (*p* = 0.61), but significantly lower than in wild-type cells (*p* = 0.0003; Table 2A).

**FIGURE 3:**
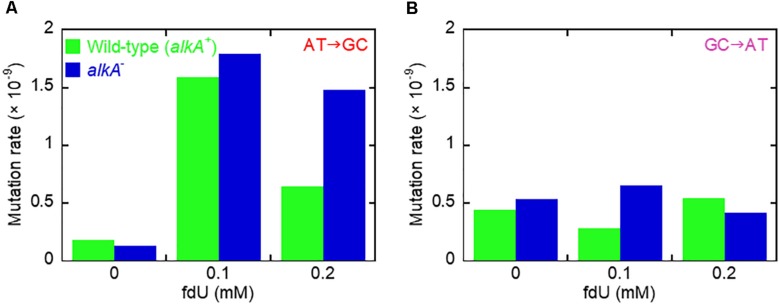
Mutation induction by fdU involves mutagen concentration-dependent alleviation by AlkA fU-DNA glycosylase. **(A)** The major base substitution induced by fdU, A⋅T → G⋅C, is alleviated by AlkA at 0.2 mM fdU. **(B)** AlkA seems to alleviate G⋅C → A⋅T transitions at 0.1 mM fdU.

Allocating the mutation rate (Table 1A) to the percent occurrence of each type of mutation (Table 2A) determines the quantitative contribution by each mutation and substitution type to mutagenesis (Table 2B and Supplementary Figure S2). This shows that the twofold increase in the mutation rate in the wild-type and *alkA*^–^ cells caused by 0.1 mM fdU (Table 1A) was mostly due to induction of the A⋅T → G⋅C transition (Table 2B). This substitution rate increased 9- and 14-fold higher than the spontaneous level in wild-type and *alkA*^–^ cells, respectively (Supplementary Figures S2B,C). However, while this rate was modestly lower at 0.2 than at 0.1 mM fdU in the *alkA*^–^ cells (Supplementary Figure S2C), it was less than half of the rate at 0.1 at 0.2 mM fdU in wild-type cells (Supplementary Figure S2B). Since the difference in A⋅T → G⋅C abundance in wild-type and *alkA*^–^ cells (Table 2A) was statistically significant, we conclude that AlkA alleviates A⋅T → G⋅C induction at 0.2 mM fdU (Figure 3A), resulting in a lower total mutation rate (Table 1A).

In *xth^–^ nfo^–^* cells, the A⋅T → G⋅C transition contributed to half the total base substitution rate at 0.1 mM fdU, while the A⋅T → G⋅C transition rate decreased to the spontaneous level at 0.2 mM fdU (Table 2B). The higher fdU load caused a bias toward the A-rule mutations, G⋅C → A⋅T and G⋅C → T⋅A (Figure 2), since the base substitution rate was the same at the two fdU concentrations (Table 2B and Supplementary Figure S2D). The higher number of unrepaired AP sites expected at 0.2 mM fdU is likely unable to be repaired efficiently in the absence of the major AP endonucleases, thus leaving behind more unrepaired AP sites to be bypassed by trans-lesion synthesis (Figure 2).

In conclusion, the A⋅T → G⋅C transition is the principal mutation induced by 0.1 mM fdU in *E. coli*, due to its large increase in abundance compared to other base substitutions (Table 2A). A significantly reduced A⋅T → G⋅C transition rate in the *xth^–^ nfo^–^* cells (Table 2B and Supplementary Figure S2D) suggests that the accumulation of unrepaired AP sites favors the A-rule mutations (Figure 2). The increased formation and A⋅T → G⋅C rate in *alkA*^–^ compared to wild-type cells at 0.2 mM fdU (Figure 3A), which theoretically should be connected to poor fU repair (Bjelland et al., 1994; Masaoka et al., 1999), suggests that AlkA initiates fU repair in DNA *in vivo* to alleviate fdU-induced mutations.

### G*⋅*C → A*⋅*T and G*⋅*C → T*⋅*A Were the Second and Third Most Common Mutations Induced by fdU

The G⋅C → A⋅T transition was the most abundant spontaneous base substitution, occurring at a similar fraction in wild-type and *alkA*^–^ cells (*p* = 0.87; Table 2A). Although the higher fraction in the *xth^–^ nfo^–^* cells accords with the A-rule (Figure 2), the difference was not significant (*p* = 0.23).

The G⋅C → A⋅T fraction at 0.1 mM fdU in wild-type was about half the fraction in *alkA*^–^ cells (*p* = 0.17; Table 2A), resulting in a 2.3-fold higher mutation rate in repair-deficient cells (Table 2B, Supplementary Figures S2B,C, and Figure 3B). However, a larger sample size is required to verify the possible G⋅C → A⋅T alleviation by AlkA. In contrast, the G⋅C → A⋅T fraction was twice as high in wild-type than in *alkA*^–^ cells at 0.2 mM fdU. However, this significant difference (*p* = 0.042) resulted in an only slightly higher transition rate (Figure 3B).

In *xth^–^ nfo^–^* cells, the G⋅C → A⋅T fraction at 0.1 mM fdU was significantly higher (*p* = 0.0042) than in wild-type cells. However, this difference decreased with 0.2 mM fdU (*p* = 0.11; Table 2A). The resulting two to threefold increase in the G⋅C → A⋅T rate with fdU in *xth^–^ nfo^–^* cells compared to wild-type cells (Supplementary Figures S2B,D, and Table 2B) could be explained if mutations formed by fU are alleviated by AlkA-initiated BER in wild-type cells, while mutations are formed via AP sites in *xth^–^ nfo^–^* cells lacking AP endonucleases (Figure 2). However, no firm conclusion can be made whether G⋅C → A⋅T is alleviated by AlkA (Figure 3B).

We previously showed that the G⋅C → A⋅T transition is the second most common base substitution induced by fdU in wild-type *E. coli* (Ånensen et al., 2001). Our present results support this finding.

The G⋅C → T⋅A transversion amounted to 13–15% of the spontaneous base substitutions in all three genotypes (Table 2A). Addition of 0.1 mM fdU significantly decreased the mutated fraction to about 4.5% in wild-type and *alkA*^–^ cells (*p* = 0.053 and 0.034, respectively). In contrast, addition of 0.2 mM fdU slightly increased the G⋅C → T⋅A fraction in wild-type (*p* = 0.66) and *alkA*^–^ cells (*p* = 0.82). Our results show no difference between the G⋅C → T⋅A transition in wild-type and *alkA*^–^ cells with 0, 0.1, or 0.2 mM fdU (*p* = 1) suggesting that AlkA minimally influences mutagenesis.

In *xth^–^ nfo^–^* cells, 0.1 mM fdU decreased the G⋅C → T⋅A fraction by about half (*p* = 0.26), while 0.2 mM fdU doubled the fraction (*p* = 0.086; Table 2A) indicating a slight mutation induction. Compared to the wild-type fractions at 0.1 and 0.2 mM fdU, these values are not significant (*p* = 0.72 and 0.22, respectively). However, at 0.2 mM fdU, the resulting G⋅C → T⋅A rate in *xth^–^ nfo^–^* cells doubled compared to wild-type cells (Table 2B and Supplementary Figures S2B,D), according to the A-rule (Figure 2).

The G⋅C → T⋅A transversion rate decreased similarly (times 0.6–0.9) with 0.1 mM fdU in wild-type, *alkA*^–^, and *xth^–^ nfo^–^* cells, but increased 1. 7-, 2. 2-, and 3.4-fold, respectively, with 0.2 mM fdU (Table 2B and Supplementary Figures S2B–D). This confirms our previous observation that the G⋅C→T⋅A transversion is the third most prevalent base substitution in wild-type *E. coli* (Ånensen et al., 2001), and suggests that unrepaired AP site accumulation also slightly affects transversion generation.

### A*⋅*T → C*⋅*G, G*⋅*C → C*⋅*G, and A*⋅*T → T*⋅*A Were Not Significantly Induced by fdU

The A⋅T → C⋅G transversion was the most distinct spontaneous mutation observed, as it roughly occurred one order of magnitude more often without mutagen compared to the few mutants observed in fdU-exposed cells, while the G⋅C → C⋅G transversion was the most rare mutation no matter whether fdU was present or not. The A⋅T → T⋅A transversion was the second least common spontaneous base substitution recorded, and in the presence of fdU its fraction decreased rather than increased (Table 2A). These results largely accord with our previous study showing no induction of G⋅C → C⋅G and weak induction of A⋅T → C⋅G and A⋅T → T⋅A by fdU in wild-type *E. coli* (Ånensen et al., 2001).

### Exposure to fdU Causes Increased *alkA* Expression

**FIGURE 4:**
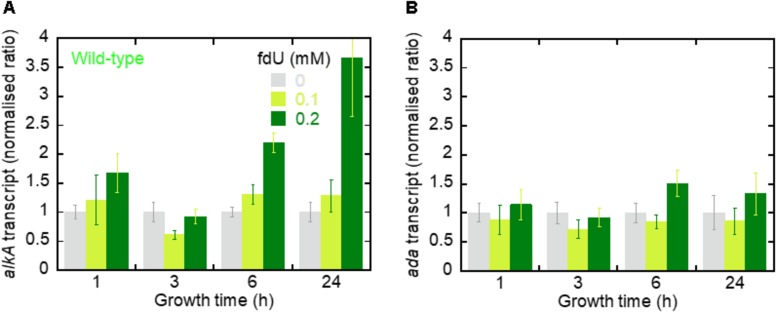
The *alkA* transcript is induced by fdU exposure. **(A)** The *alkA* transcript expression levels (fold changes) in *E. coli* wild-type as a function of growth time with 0, 0.1, and 0.2 mM fdU. Target gene expression (*alkA*) is normalized to the endogenous *tag* gene. The columns represent the mean value ± SD for three independent experiments. **(B)** *ada* transcript expression levels (fold changes). Target gene expression (*ada*) is normalized to the endogenous *tag* gene. The columns represent the mean value ± SD for three independent experiments.

Since fdU-induced mutagenesis is alleviated by AlkA, we asked whether *alkA* gene expression is also induced by fdU. Thus, relative *alkA* gene expression was determined by RT-qPCR in wild-type *E. coli* with and without fdU treatment up to 24 h. *alkA* transcript remained at the same level after treatment with 0.1 mM fdU, even after 24 h exposure. This contrasted with a clear time-dependent induction of *alkA* with the highest fdU concentration (Figure 4A). Exposure to 0.2 mM fdU for 6 h increased *alkA* expression 2.2-fold, and 3.7-fold after 24 h.

We then analyzed *ada* gene expression, which controls the *alkA* gene (Lindahl et al., 1988; Landini and Volkert, 2000; Mielecki et al., 2015). We observed no difference in *ada* expression across most fdU concentrations and time durations (Figure 4B). The only exception was after 6 h, where 0.2 mM fdU treatment increased *ada* expression 1.5-fold. Thus, treating *E. coli* wild-type cells with 0.2 mM fdU induces *alkA* gene transcription.

### AlkA-Mediated fU Excision From DNA Is Dependent on the Complementary Base

**FIGURE 5:**
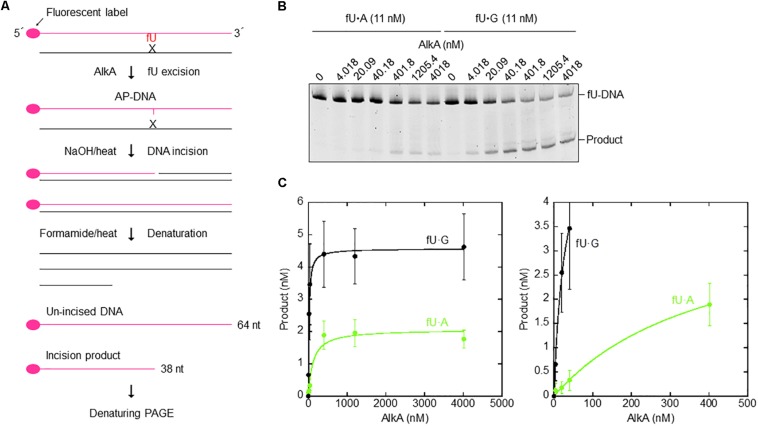
Single-turnover kinetics for AlkA-mediated fU excision opposite A and G in DNA. **(A)** DNA substrates and assays (see the section “Materials and Methods”). **(B)** AlkA was incubated with 11 nM fU⋅A (Left) or fU⋅G (Right) substrate at 37°C for 60 min in 50 μL reaction buffer. Electrophoresis was performed on a 20% (w/v) polyacrylamide gel containing 7 M urea at 500 V for ∼1.5 h. **(C)** Product (fU) formation as a function of enzyme concentration (Left); a magnified image of product formation when approaching enzyme saturation (Right). Each value represents the average of seven independent measurements.

AlkA protein was purified (Supplementary Table S3) to apparent physical homogeneity (Supplementary Figure S3) as described in the section “Materials and Methods.” To analyze the efficiency of fU excision, a defined, 64 nt double-stranded DNA oligonucleotide with a central fU opposite A or G (Figure 5A) was used as an AlkA substrate. Significant NaOH-mediated damaged strand cleavage was observed in the presence of enzyme. However, the amount of enzyme needed to create alkaline labile AP sites in DNA was highly dependent on the opposite base. Kinetic analysis showed that the fU⋅G substrate was cleaved 17 times more efficiently than the fU⋅A substrate (Figures 5B,C), according to the *k*_*cat*_*/K_*D*_* value (Table 3). More efficient fU excision opposite G compared to A was also reported for human SMUG1 (Knævelsrud et al., 2009), and agrees with the widely accepted flipping-out mechanism for DNA glycosylase action. In this mechanism, the enzyme has to disrupt fU base pairing and base-stacking interactions in double-stranded DNA to be accommodated in the active site pocket (McCullough et al., 1999; Wibley et al., 2003).

**TABLE 3:** Kinetic parameters for the opposite-base dependent fU excision by AlkA.

**Opposite base**	***k*_*cat*_ (min^–1^)**	***K*_*D*_ (nM)**	***k*_*cat*_/*K*_*D*_ (min^–1^/nM) [fold]**
A	0.028 ± 0.002	116.8 ± 51.5	0.0002 [1]
G	0.054 ± 0.002	13.22 ± 3.14	0.0041 [17]

*The standard error of the mean (SEM) is indicated for five independent experiments, estimated as a function of increasing enzyme concentration.*

### The Keto, but Not the Ionic Form, of fU Is Efficiently Accommodated in the AlkA Active Site Pocket, and Is Indicated by the fU Release pH Dependency Profile

**FIGURE 6:**
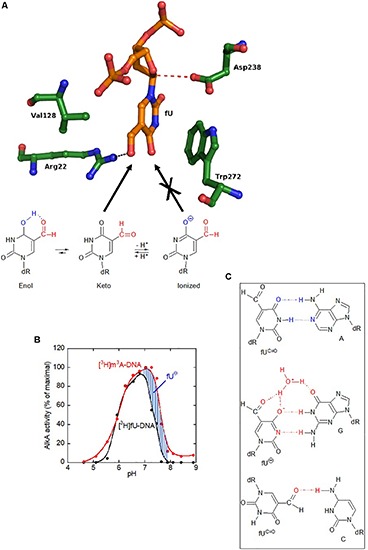
The AlkA active site does not accommodate the ionized form of fU, which erroneously pairs with guanine. **(A)** Docking of the keto, but not the ionic form of fU, into the AlkA substrate-binding pocket. Unlike primary substrate m^3^A docking, no stacking occurs between fU and Trp272. Instead, the fU formyl group forms a hydrogen bond to Arg22. Regarding m^3^A, Asp238 is positioned to attack the N1–C1 bond. The abundance of the ionized (⊖) form of fU increases with pH (lower panel). While the AlkA active site pocket accommodates the fU keto form, it excludes the ionized form (not shown). **(B)** Exclusion of fU^⊖^ from the AlkA active site was inferred from enzymatic fU release. AlkA fU- and m^3^A-DNA glycosylase activity as a function of pH shows a much stronger decline in the activity for fU than for m^3^A at high pH, which is explained by the higher abundance of non-excisable fU^⊖^. AlkA (2 pmol and 0.08/0.16 pmol) was incubated with aged [*methyl*-^3^H]thymine-labeled (11 independent experiments) and [^3^H]MNU-treated DNA (4 independent experiments), respectively. **(C)** Postulated base pairing properties of fU residues in DNA. Since fU is a thymine analog that efficiently pairs with adenine, the fU⋅A pair is most likely to occur *in vivo*. The fU⋅G mispair formed by the ionized form (fU^⊖^) is stabilized by a water bridge. Both these base pairs have been demonstrated by X-ray analyses of DNA oligomers with fU inserted at a specific site (Tsunoda et al., 2001, 2002). However, the fU⋅C mispair is tentative (Kamiya et al., 2002). Cognate base pairing with adenine is due to the keto form (fU^C=O^). Cognate hydrogen bonding pattern is shown in blue; non-cognate hydrogen bonding/base pairing pattern in red.

To indicate how AlkA accommodates fU in the substrate-binding pocket (Figure 6A, upper panel), AlkA served as receptor in automated docking simulations. Following sampling of 250 possible docking runs, fU showed a docking energy distribution, with several runs clustering into the lowest energy bin. For the primary AlkA substrate, m^3^A, this result was even more pronounced. The fewer successful docking runs obtained with fU may reflect a reduced binding affinity compared to m^3^A. However, successful m^3^A docking was dependent on its positive charge (extensively described in Supplementary Material, section “Docking m^3^A and fU in the Binding Pocket of *E. coli* AlkA” and Supplementary Figures S4–S10). In the specific conformation with the lowest docking energy, the formyl group in the 5-position of the uracil ring forms a hydrogen bond to the Arg22 side chain in the bottom of the binding pocket (Figure 6A, upper panel), which appears to stabilize binding of the neutral fU keto form (fU^*C=O*^). Unlike m^3^A (Supplementary Figure S4), π–π stacking with the Trp272 side chain does not appear to be important for fU binding. Importantly, if fU adopts the ionic form (fU^⊖^), where the negative charge is delocalized to the uracil ring (Figure 6A, lower panel), the overall sum of repulsive forces to the catalytic residues Asp238 and Trp272 could outweigh the potentially attractive forces to Arg22 (Figure 6A, upper panel), thus excluding fU^⊖^ from the active site pocket.

To provide chemical evidence for *in silico* fU^⊖^ exclusion from the active site pocket, AlkA activity for radioactively labeled fU in DNA was measured as a function of pH. Since fU^⊖^ abundance increases with pH, its rejection from active site should cause decreased enzyme activity for fU, compared to m^3^A, which exists in one chemical form. Interestingly, a large decrease in DNA glycosylase activity occurred at pH 7.25 for fU, rather than pH 7.6 for m^3^A (Figure 6B), which supports the molecular docking simulation results showing fU^⊖^ exclusion from the active site pocket.

## Discussion

**FIGURE 7:**
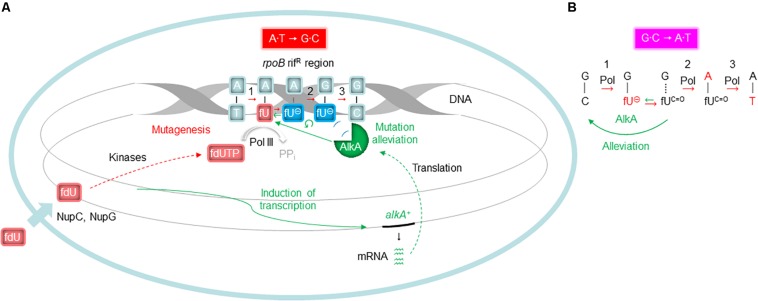
Proposed molecular events influencing fdU mutagenesis alleviation by AlkA. **(A)** Alleviation of A⋅T → G⋅C (Figure 3A) needs *alkA* induction (Figure 4A), due to low *k*_*cat*_/*K*_*D*_ for fU⋅A (Table 3). Since the common fU keto form (fU^C=O^) primarily behaves like thymine, the corresponding fdUMP is most efficiently incorporated into DNA opposite adenine (replicative event 1). If this newly inserted fU is removed by AlkA, the mutation-initiating event is reversed or alleviated. Alternatively, when some fU^C=O^ paired with A transforms to the ionic state (fU^⊖^), AlkA recognition is excluded (Figure 6A) in favor of polymerase binding, leading to guanine insertion opposite fU^⊖^, causing mutagenesis (replicative events 2 and 3). **(B)** The possible alleviation of G⋅C → A⋅T (Figure 3B) takes place without *alkA* induction (Figure 4A) because of high *k*_*cat*_/*K*_*D*_ for fU⋅G (Table 3). Although the most frequent replicative event (event 1 in A) is fdUTP pairing with adenine, fdUTP will pair with template guanine if it adopts the ionic state (replicative event 1). When the newly inserted fU^⊖^ reverts to fU^C=O^, it will be an efficient target for AlkA reversing or alleviating the mutation-initiating event. If replication takes place before AlkA reaches fU, an adenine will usually be inserted, causing mutagenesis (replicative events 2 and 3). Events leading to base substitutions are in red; events promoting alleviation of mutagenesis are in green. Processes demonstrated in this study are shown by unbroken arrows and otherwise by broken arrows; ↺, exclusion from AlkA active site. Plasma membrane with nucleoside transporter (short thick arrow at the bottom left) is shown in light blue. PP_*i*_, pyrophosphate. See Figure 2 for definitions for the other symbols.

In spite of several studies establishing AlkA as the major glycosylase to initiate fU BER in DNA (Bjelland et al., 1994; Masaoka et al., 1999; Terato et al., 1999), no evidence has emerged indicating such a role *in vivo*. In fact, it appeared strange to allocate this function to a protein whose expression is induced by sub-lethal methylating agent concentrations, and is dependent on enhanced *ada* expression (Evensen and Seeberg, 1982; Karran et al., 1982; Sedgwick, 2004; Shrivastav et al., 2010). To gain insight into this issue, we investigated whether BER influences fU-induced mutagenesis. We added fdU to *E. coli* growth cultures to specifically introduce fU into the DNA of repair proficient (wild-type; *alkA^+^ xth^+^ nfo^+^*) and deficient (*alkA*^–^ and *xth^–^ nfo^–^*) cells, and systematically determined the mutation rate and spectrum. To bind the *E. coli* plasma membrane transporter NupC, a C3′-hydroxyl group is required, while binding to NupG is dependent on a hydroxyl group at both the C3′ and C5′-position (Patching et al., 2005). Thus, fdU is most likely taken up from the culture medium by both these proteins (Figure 7A). We chose to select for rifampicin-resistant cells formed as a result of a targeted mutation in the *rpoB* rif^R^ region, which was then sequenced using a single mutant clone collected from each experiment. The spontaneous mutation spectrum obtained from wild-type cells (Figure 1A, green) shows the distribution of the six possible base substitutions (Table 2A). The distribution was very similar to recent results from a comprehensive whole-genome sequencing analysis of several *E. coli* wild-type strains (Foster et al., 2015). These data confirm that the rif^R^ system is adequate for such investigations.

Our results confirm that the A⋅T → G⋅C transition is the principal mutation arising from fU base lesions in DNA (Table 2A and Figures 1B–D) due to occasional mispairing with guanine (Figure 6C, middle panel). The connection with the fU base is indicated by lower formation in the *xth^–^ nfo^–^* mutant (Table 2B and Supplementary Figure S2D). The predominance of A⋅T → G⋅C at low fdU (0.1 mM) is challenged by G⋅C → A⋅T at higher (0.2 mM) fdU concentration (Table 2A) in the wild-type, but not the *alkA*^–^ cells (Supplementary Figures S2B,C, respectively, and Table 2B). The G⋅C → A⋅T transition is a typical A-rule mutation that is also formed via AP sites (Figure 2), which is underscored by its consistently higher rates in the *xth^–^ nfo^–^* cells (Table 2B and Supplementary Figure S2D). However, trans-lesion synthesis past AP sites formed following enzymatic fU removal does not appear to be the single major mechanism generating G⋅C → A⋅T after fdU incorporation into DNA, because AlkA deficiency causes a similar increase in the (0.1 mM) fdU mutation rate as AP endonuclease deficiency (Supplementary Figures S2C,D, respectively). Indeed, fewer AP sites should form from fU in *alkA*^–^ cells compared to wild-type cells. As previously shown (Ånensen et al., 2001), the present results confirm that the G⋅C → A⋅T transition is an important fU-induced mutation formed by occasional non-cognate fdUMP incorporation opposite guanine in DNA (Figure 2). The G⋅C → T⋅A transversion is also an A-rule mutation (Figure 2), as confirmed by its higher rate in *xth^–^ nfo^–^* cells compared to wild-type cells at 0.2 mM fdU (Table 2B and Supplementary Figures S2B,D). The increased mutation rate in *alkA*^–^ cells indicates that mechanisms other than trans-lesion synthesis past AP sites are involved in G⋅C → A⋅T transition generation. Although our data lacks statistical significance due to the insufficient number of experiments required for this particular mutant, the results corroborate previous studies showing that G⋅C → T⋅A transversions arise in *E. coli* (Fujikawa et al., 1998; Ånensen et al., 2001) because of mispairing between fU and cytosine (Zhang et al., 1997; Figure 6C, lower panel). In our study, this was likely due to occasional non-cognate fdUMP incorporation opposite cytosine in DNA (Figure 2). Consequently, mutations change from mostly A⋅T → G⋅C at low fdU (0.1 mM) to additional G⋅C → A⋅T and G⋅C → T⋅A at higher fdU (0.2 mM) concentrations. The other possible base substitutions, A⋅T→C⋅G, A⋅T → T⋅A, and G⋅C → C⋅G, were not significantly induced by fdU in this investigation (Table 2A), which confirms our previous results showing that the two former are less efficiently formed, while the latter is not formed by fdU (Ånensen et al., 2001).

We made some mechanistic conclusions regarding the fdU-induced base substitutions based on the abundance in repair proficient and deficient strains. However, the major topic of the investigation was to investigate whether AlkA-initiated BER counteracts fdU-mediated mutagenesis. The results show that AlkA significantly influences mutagenesis, and that this *in vivo* effect is dependent on the mutagen concentration in the culture medium. At the lowest fdU concentration (0.1 mM), *alkA*^–^ cells experienced a similar mutation rate as wild-type cells (Table 1A). Although we observed an alleviating AlkA effect on G⋅C → A⋅T induction that was not statistically significant (Figure 3B), such lack of phenotype for fU repair can be explained by the presence of several back-up systems, like Fpg, Nth, and Nei glycosylases (Zhang et al., 2000). However, this study employed oligonucleotides with fU inserted at a specific site, which is more sensitive to glycosylase fU recognition due to lack of competition from other lesions than the multiple-lesion containing [^3^H]thymine-DNA we used in our first study comparing Fpg, Nth, and AlkA, which showed no activity for fU (Bjelland et al., 1994). Because Nei exhibits similar fU activity as Nth (Zhang et al., 2000), and only AlkA exhibited clearly detectable fU activity using [^3^H]thymine-DNA, we conclude that AlkA is superior to Fpg, Nth, and Nei in removing fU from DNA. In contrast, we observed significantly different A⋅T → G⋅C transition levels between wild-type and *alkA*^–^ cells at 0.2 mM fdU (Table 2A) resulting in a more than twice as high mutation rate (Figure 3A). These results provide evidence that AlkA alleviates fdU-induced mutagenesis (Figure 7A). It was especially interesting that the *alkA* gene is indeed induced at 0.2 mM fdU (Figure 4A), explaining the higher impact of AlkA on mutagenesis at this concentration. It should be noted that the A⋅T → G⋅C rate increase in *alkA*^–^ compared to wild-type at 0.2 mM fdU is in the same order of magnitude as the more than fivefold increase in total methyl methanesulfonate (MMS)-induced mutagenesis in the former compared with that in the latter cell type, although this was investigated under different conditions using another mutagenicity tester system (Grzesiuk et al., 2001).

The *alkA* gene is induced by sub-lethal concentrations of alkylating agents (Evensen and Seeberg, 1982; Karran et al., 1982; Teo et al., 1986; Sakumi and Sekiguchi, 1989; Saget and Walker, 1994; Landini and Volkert, 2000; Kleibl, 2002; Mielecki et al., 2015). While SN1 and SN2 methylating agents like *N*-methyl-*N*′-nitro-*N*-nitrosoguanidine (MNNG) and MMS (Sedgwick, 2004) cause a 5–20-fold increase in *alkA* expression or AlkA activity (Evensen and Seeberg, 1982; Karran et al., 1982; Baek et al., 2009; Booth et al., 2013), we detected a nearly fourfold increase by fdU when normalized to the constitutively expressed *tag* gene (Figure 4A), which encodes m^3^A-DNA glycosylase I (Sakumi et al., 1986; Bjelland and Seeberg, 1987). Whether this increase is dependent on regulation by the Ada protein is questionable, since fdU only caused a minimal increase in *ada* expression (Figure 4B). The hypothesis that *alkA* is only induced by alkylation exposure was also challenged by a previous study, showing that RNA polymerase associated with the stationary phase σ^s^ instead of the normal σ^70^ transcription factor transcribes *alkA* independent of *ada* (Landini and Busby, 1999). It has been calculated that an un-adapted *E. coli* cell contains ∼50 AlkA molecules (Bjelland et al., 1994). If we consider that the 3.7-fold increase in transcripts (Figure 4A) causes a similar increase in protein concentration, fdU increased AlkA expression to ∼200 molecules per cell in our experiments, which equals the concentration of Tag in *E. coli* (Bjelland and Seeberg, 1987). Tag and AlkA have similar activity for m^3^A, which is the primary substrate for both glycosylases. Thus, both the cellular concentration of AlkA caused by fdU and the significant effects on fdU-mediated mutagenesis caused by AlkA inactivation suggest a significant contribution by this enzyme in modulating some ROS-mediated effects.

Although we have demonstrated a connection between the fU-DNA glycosylase activity of AlkA, *alkA* gene induction, and fdU-induced mutagenesis, *in vivo* evidence for the participation of other glycosylases like Fpg, Nth, Nei, and Mug in *E. coli* fU repair is elusive (Zhang et al., 2000; Liu et al., 2003). Since the latter enzymes seem to exhibit significantly lower *in vitro* activity than AlkA (see above), it is probably challenging to detect phenotypic differences in fdU-mediated mutagenesis between wild-type and cells with only one of these glycosylase genes inactivated. Instead, investigation of doubly and/or triply inactivated cells like *fpg^–^nth^–^* and *fpg^–^nth^–^nei^–^* might be more fruitful. Because mutagenesis is expected to mainly take place during the exponential growth phase, we judge Mug as less important due to its function during the late stationary phase of cells (Mokkapati et al., 2001). Interestingly, the KsgA enzyme exhibits DNA glycosylase activity for C opposite fU and potentially might promote G⋅C → T⋅A transversions (Figure 2). In addition, mismatch repair is probably involved in mutation prevention because the fU⋅G mismatch is recognized by MutS protein (Terato et al., 1999). The mammalian nucleotide excision repair component XPC-HR23B recognizes fU in DNA (Kino et al., 2004), which calls for examination of the analogous UvrABC system of *E. coli* (Van Houten et al., 2005).

In addition to showing *alkA* gene induction by fdU and demonstrating that AlkA alleviates mutagenesis caused by this oxidized nucleoside, our findings also provide mechanistic understanding of how fdU shifts the *E. coli* mutation spectrum to mainly A⋅T → G⋅C transitions (Table 2A). A major breakthrough came with the description of stable reversed wobble type pairing between fU^⊖^ and guanine (Figure 6C). *In silico* modeling showed this pairing is accommodated by DNA polymerase (Tsunoda et al., 2001, 2010), providing the molecular basis for both A⋅T → G⋅C and G⋅C → A⋅T mutations. Here, we substantiate this finding with biochemical evidence (Figure 6B) that fU^⊖^ is also excluded from the AlkA active site pocket (Labahn et al., 1996; Yamagata et al., 1996), further leading to polymerase 2′-deoxyguanosine monophosphate (dGMP) mis-insertion, which primarily causes A⋅T → G⋅C transitions due to the ability of the major fU^*C=O*^ species to first pair with A. Actually, we believe that the fU^⊖^ inaccessibility to the AlkA active site pocket is essential for allowing polymerase access and enough time for dGMP mis-incorporation (Figure 7A). It is interesting to note that to alleviate this mutation at 0.2 mM fdU, *alkA* gene expression is required (Figure 4A), which might be related with the low *k*_*cat*_/*K*_*D*_ for fU⋅A (Table 3). This can be observed by the slight AlkA alleviation offered by 0.1 mM fdU without *alkA* induction (Figure 3A). Similarly, the possible alleviation of the G⋅C → A⋅T transition (Figure 7B) at 0.1 mM fdU (Figure 3B), which occurs without *alkA* induction (Figure 4A), may be explained by the nearly 20 times higher *k*_*cat*_/*K*_*D*_ for fU⋅G compared to fU⋅A (Table 3). As indicated by the predominance of the A⋅T → G⋅C transition, most fdUMP is inserted opposite adenine in DNA during replication. When inserted fU is converted to the ionized form, DNA polymerase processes the lesion, inserting a G opposite fU, instead of AlkA (Figure 7A). By contrast, how may the efficient excision of fU opposite G (Table 3) occur, when the fU^⊖^ ⋅G pair should be stable (Figure 6C, middle panel)? This can be explained by the favored conversion of fU^⊖^ to the keto form, which disrupts the hydrogen bonding to G. This results in efficient “flipping out” and fU excision by AlkA, possibly alleviating the G⋅C → A⋅T transition.

## Conclusion

This is the first report demonstrating that an oxidized deoxynucleoside (fdU) at a certain concentration in the culture medium induces expression of the *alkA* gene in *E. coli*, resulting in alleviation of mutagenesis caused by that agent. Specifically, the occurrence of the A⋅T → G⋅C transition, which is the primary mutation caused by fdU, is reduced to half by *alkA* gene induction. Thus, for the first time we show *in vivo* evidence for the participation of the AlkA DNA glycosylase in the repair of base lesions damaged by oxidation. Our study also elaborates a novel mutation mechanism in *E. coli*, which may be used to explain other instances of fU-mediated mutagenesis. Further studies are required to clarify the molecular mechanisms involved in the fdU-mediated *alkA* gene induction and search for links to concomitant changes in cell physiology.

## Data Availability Statement

All datasets generated for this study are included in the article/Supplementary Material.

## Author Contributions

KG and IM-L designed and carried out the mutagenesis experiments, analyzed the data, and wrote parts of the manuscript. IK, HÅ, and IA designed and carried out the mutagenesis experiments and analyzed the data. AT designed and carried out the RT-PCR experiments, analyzed the data, and wrote parts of the manuscript. MA carried out the RT-PCR experiments and analyzed the data. GH produced and characterized the AlkA enzyme and wrote parts of the manuscript. IL performed the *in silico* analyses and wrote parts of the manuscript. KG, IK, and PR performed the kinetic analysis. JK performed the statistical analyses and wrote parts of the manuscript. KS and AM produced and characterized fdU and the fdU-containing DNA. IA and AK suggested and contributed to experiments, and revised the manuscript. SB conceived the project, designed the experiments, analyzed the data, supervised and managed the study, and wrote the manuscript. All authors took part in the analysis of data and approved the final version of the manuscript.

## Conflict of Interest

The authors declare that the research was conducted in the absence of any commercial or financial relationships that could be construed as a potential conflict of interest.
