# Supplementary material for: The Escherichia coli alkA Gene Is Activated to Alleviate Mutagenesis by an Oxidized Deoxynucleoside
Source: Front Microbiol. 2020 Feb 25;11:263. doi: 10.3389/fmicb.2020.00263 (PMC7051996; doi:10.3389/fmicb.2020.00263)
Supplement: Supplementary file 1 [file Data_Sheet_1.pdf]

Supplementary Material

1 Supplementary Results

1.1 Base Substitutions

We detected base substitutions at 25 different sites in the rif^R region. The numbering scheme was adopted from a previous report describing 24 base substitution mutations (Garibyan et al., 2003). The two extra positions are 1575 (**Figure 1A**) and 1597 (**Figure 1C**). In addition to the latter sites, mutations occurred infrequently (1–4 recorded) at positions 1527, 1532, 1585, 1600, 1601, 1716, and 1721 (**Figures 1A–D**). By defining hot spot as harboring ~10% or more of the total mutations detected in each bacterial cell type, 8 positions were identified for spontaneous mutations in wild-type: 1535, 1546, 1547, 1576, 1592, 1687, 1691, and 1714 (**Figure 1A**). For wild-type and *alkA*⁻ cells, 80 and 85% of the spontaneous mutations were respectively located at these sites, compared to 74% for *xth*⁻ *nfo*⁻ cells. However, position 1691 was not a spontaneous mutation hot spot in *xth*⁻ *nfo*⁻ cells (2 mutations recorded), and no mutation was observed at position 1687 (**Figure 1D**).

1.2 Indels

Five spontaneous indels were observed (**Table 2A**). Wild-type clones exhibited triplet GCA (positions 1594–1596) and sextuplet CACTCG deletions (positions 1595–1600; **Figure 1A**). *alkA*⁻ clones exhibited sextuple TCTCCG insertion between positions 1594 and 1595, and triplet CTC insertion between positions 1589 and 1590 (**Figure 1A**). *xth*⁻ *nfo*⁻ clones exhibited a deletion of the nonet CGCACTCGG (positions 1593–1601; **Figure 1D**). Only one indel was recorded from fdU-exposed cells, *i.e.*, a wild-type cell exposed to 0.2 mM fdU (**Table 2A**) experienced a triplet AGT deletion (positions 1538–1540; **Figure 1C**).

1.3 Docking m^3A and fU in the Binding Pocket of *E. coli* AlkA

The published crystal structure of *E. coli* AlkA complexed with the double-stranded 10-nt DNA containing the abasic 1-azaribose (PDB 1 diz) (Hollis et al., 2000) formed the starting point of our automated docking analysis. The goal was to study how fU (**Figure 6A**, lower panel) could form a stable intermediate in the substrate-binding pocket. The principal AlkA substrate m^3A (**Supplementary Figure S4**) was included as a positive control. The protein part of the complex was selected as receptor, and a 3-nt single-stranded fragment with the 1-azaribose part substituted by the chosen base analog was modified into the docking ligand. A total of 3 (m^3A) and 4 (fU) rotatable bonds were selected to allow for some degree of ligand reorientation during docking (see **Supplementary Figures S5,S6**).

1.3.1. Docking Results for m^3A

For m^3A, 250 docking attempts were run. As indicated in **Supplementary Figure S7**, 65 docked conformations were found in the bin with the lowest docking energy. This clearly indicates a successful docking run, where many docking attempts cluster into the lowest energy bin. Most conformations in the lowest energy bin had the base analog neatly positioned in the substrate-binding pocket, with the side chain of the catalytically-essential Asp238 nicely aligned for cleavage of the m^3A nucleotide C1–N1 bond. In addition, the aromatic side chain of Trp272 stacks against the

electron-deficient m^3A base (see **Supplementary Figure S8**). It was intriguing to observe that for docking to be successful, the m^3A base showed an absolute requirement for being positively charged. Docking attempts with neutral m^3A were not successful. Thereafter, spreading a charge of +1 onto the atoms in the six-membered ring of m^3A caused the result shown in **Supplementary Figure S7**.

1.3.2. Docking Results for fU

For fU, 250 possible docking runs were sampled, yielding the docking energy distribution shown in **Supplementary Figure S9**. Similar, but less pronounced for m^3A, there is a distinct shift towards several docking runs clustering into the lowest energy bin. The reason for obtaining fewer successful docking runs for fU than for m^3A might simply reflect the reduced binding affinity for fU. Attempts to dock more substrates with characterized binding strength could clarify this. We obtained one distinct conformation with the lowest docking energy, as indicated in **Supplementary Figure S10**. Interestingly, the formyl group in the 5-position forms a hydrogen bond to the side chain of Arg22 in this conformation. Unlike m^3A, π–π stacking with the Trp272 side chain does not appear to be important in binding fU.

1.3.3. Supplementary Discussion and Conclusions

Automatic docking appears to work well in this case. It seems that the results obtained are reasonable. For m^3A, the presence of a positively charged substrate is sufficiently for protein binding. For neutral fU, the formation of a hydrogen bond to the side chain of Arg22 in the bottom of the binding pocket appears to be necessary to stabilize the substrate, similar to Arg286 in the homologous AfAlkA binding pocket (Leiros et al., 2007). Further docking studies and comparison to existing literature could shed light on these speculations. Optionally, mutational analysis could also clarify these speculations.

To reduce ligand size and complexity leading to excessive CPU use, a trinucleotide was used as the ligand in the automatic docking. Nevertheless, when the docked trinucleotide was super-positioned back onto the DNA fragment from the DNA–protein complex crystal structure, the DNA backbone conformation overlapped well, indicating that the DNA–protein interactions are accurately simulated in the docking.

Because the protein residue that replaces the flipped-out nucleotide is the aliphatic Leu125, no polar interactions are formed with the complementary bases. Thus, we speculate that the flipped-in stability of fU with complementary (purine) bases determines the efficiency of fU cleavage.

2. Supplementary Figures and Tables

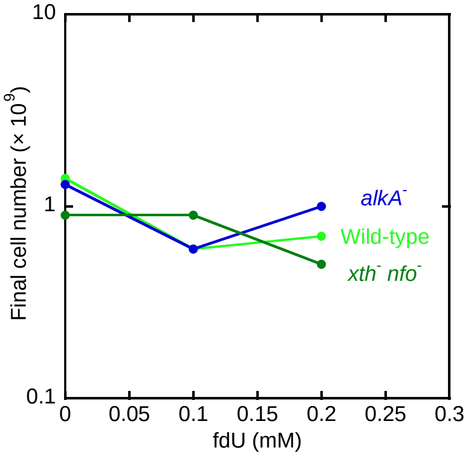

Supplementary Figure S1. The thymidine analog fdU exhibits low or moderate cytotoxicity. Final cell number (viable cells per 2 mL cell culture) of repair-proficient wild-type (*alkA*$^{+}$) and repair-deficient *alkA*$^{-}$ and *xth*$^{-}$ *nfo*$^{-}$ *E. coli* mutants as a function of fdU concentration in the growth medium. Growth started with about 9000 cells and proceeded for 44–47 h. Each point represents the median of 17–133 independent measurements. ●, Wild-type (AB1157); ●, *alkA*$^{-}$ (MS23); ●, *xth*$^{-}$ *nfo*$^{-}$ (BW528). See **Supplementary Table S1A** for details.

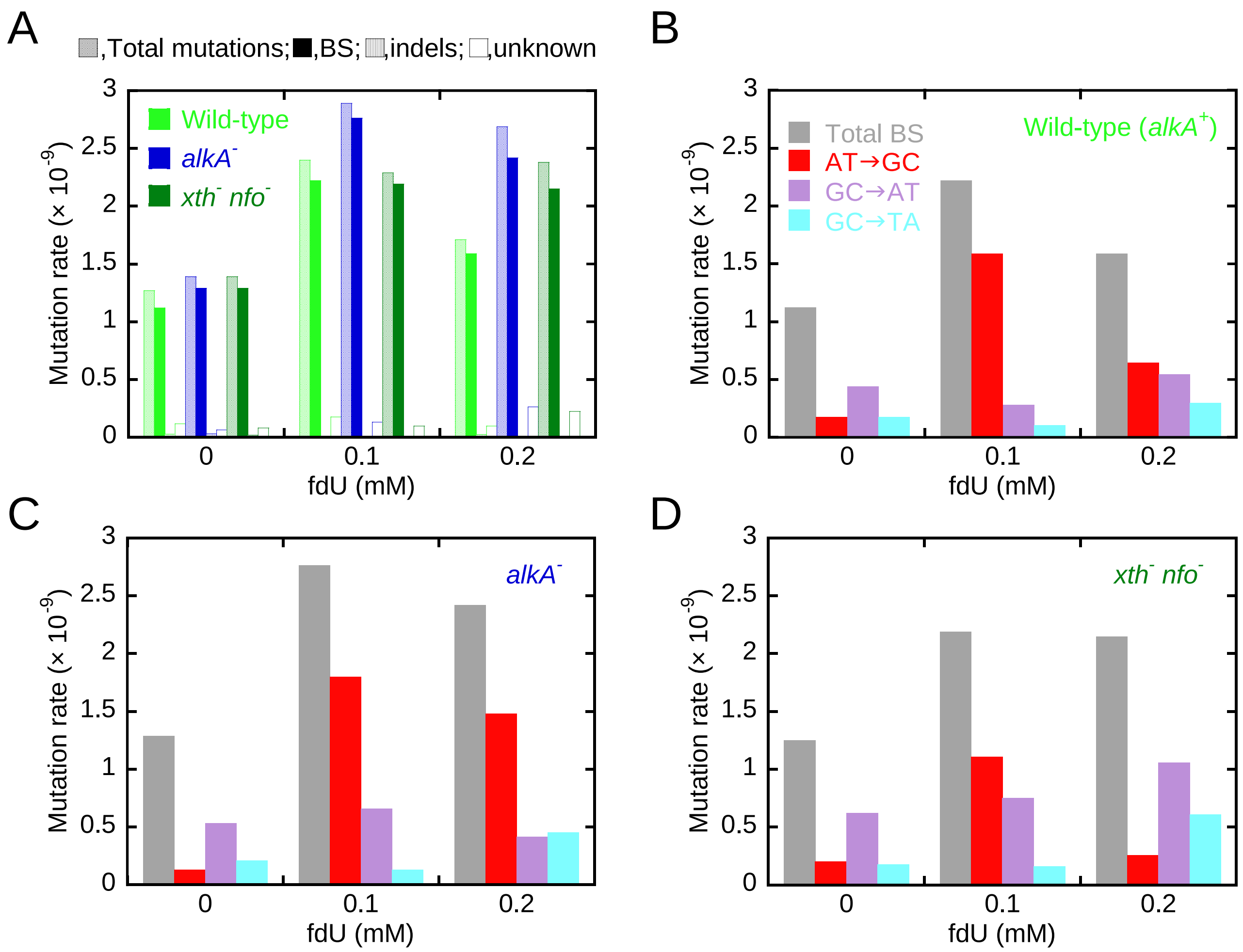

Supplementary Figure S2. Mutagenicity caused by fdU in *E. coli* is dependent on DNA repair capacity. **(A)** The contribution to the total mutation rate by base substitutions (BS), indels, and unknown mutations (*i.e.*, outside the sequenced rif^R region) in fU-DNA glycosylase-proficient ($alkA^+$, wild-type) and deficient ($alkA^-$) cells, and in AP endonuclease-deficient (xth^- nfo^-) cells. **(B)** The major fdU-induced A·T → G·C, G·C → A·T and G·C → T·A BS rates in wild-type, **(C)** $alkA^-$, and **(D)** xth^- nfo^- cells. The mutation rate (**Table 2B**) was calculated for each base substitution at each fdU concentration in the growth medium from the values in **Tables 1A,2A**.

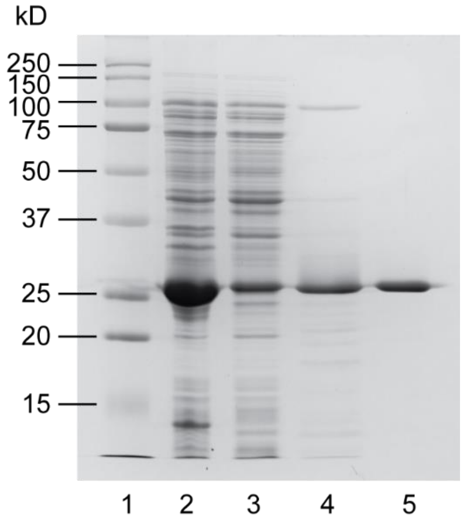

Supplementary Figure S3. SDS-polyacrylamide gel electrophoresis of protein fractions obtained during AlkA purification. Lane 1, Precision Plus Protein Kaleidoscope standard (BioRad); lane 2, cell extract; lane 3, soluble protein; lane 4, protein purified by gel filtration; lane 5, protein purified by cation exchange chromatography (1.6 µg).

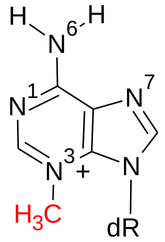

Supplementary Figure S4. The m^3A molecular structure used as a positive control. The erroneous methyl group is indicated in red. The fU structure is shown in **Figure 6A**, lower panel.

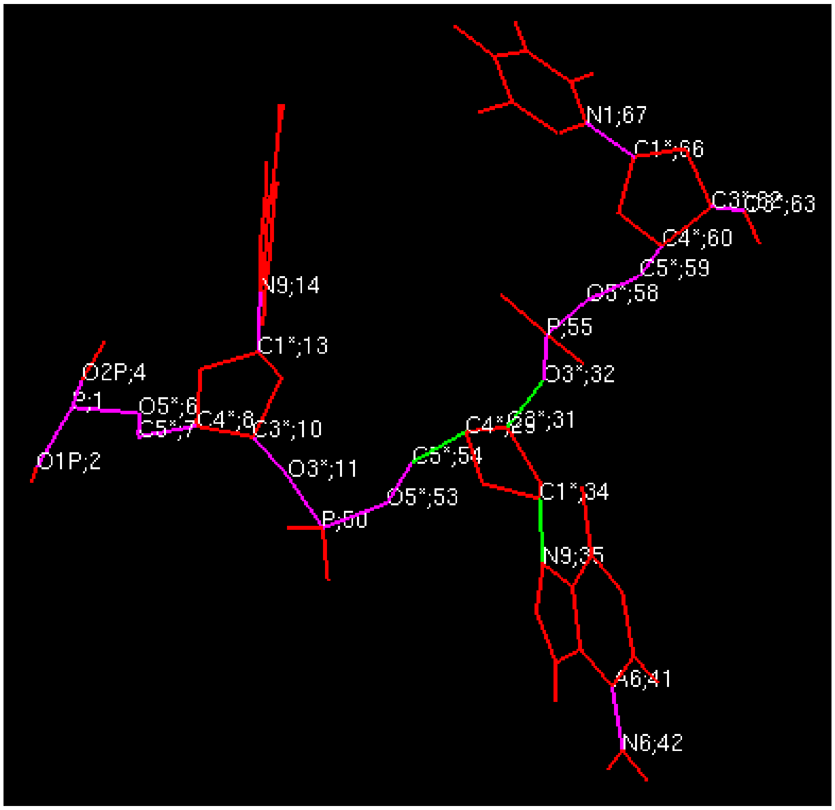

Supplementary Figure S5. Rotatable bonds shown in green for the docking of m^3A. Rotational freedom is allowed on the base and on both sides of the central ribose unit, to allow for some minor adjustments of the nucleotide into the binding pocket.

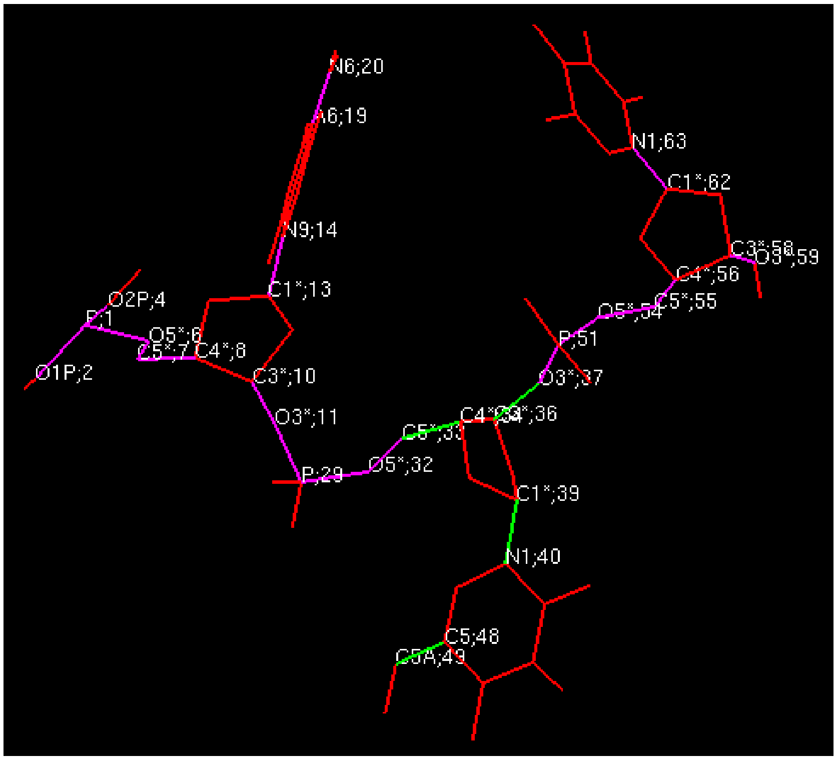

Supplementary Figure S6. For fU docking, additional rotational freedom was granted for the C5–C5A bond, putting the formyl group into play, in addition to the three rotations already mentioned for m^{3}A.

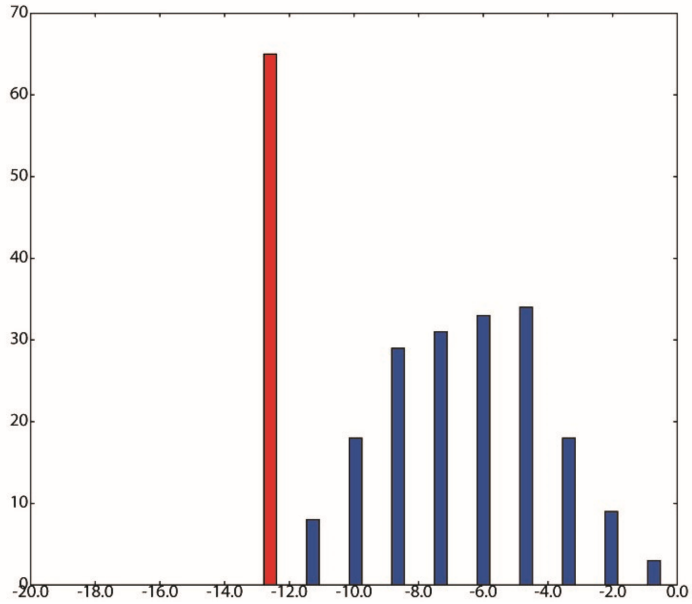

Supplementary Figure S7. Docking energy distribution for 248 docking attempts of a trinucleotide containing m^3A. The red bar shows the docking conformations with the lowest docking energy. Most conformations are conserved, such as the m^3A base analog, which fits nicely within the substrate-binding pocket.

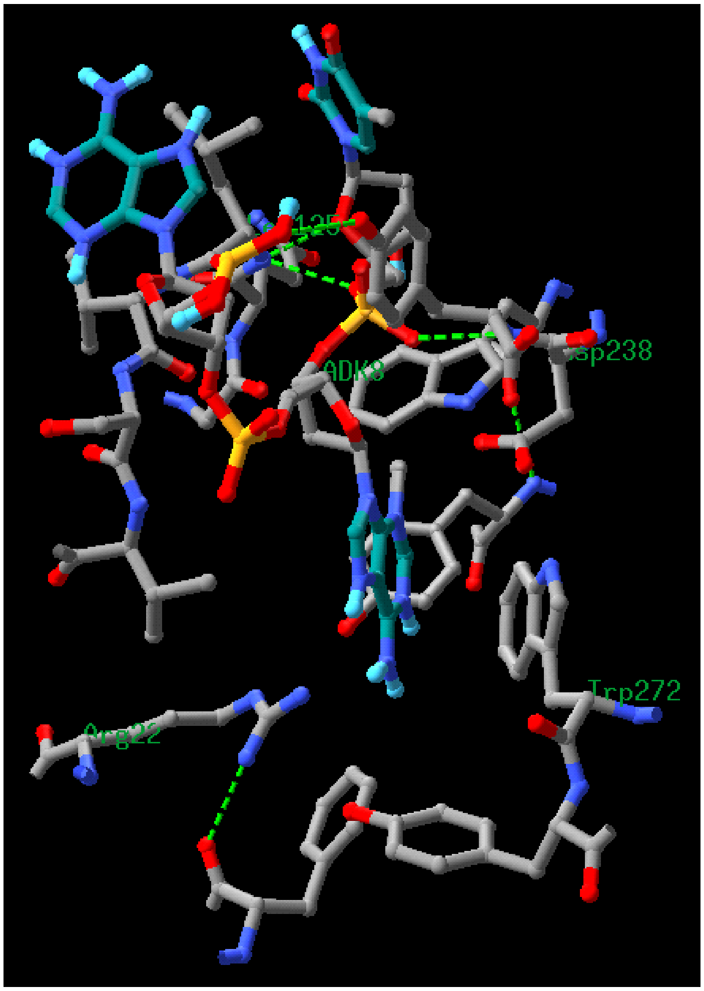

Supplementary Figure S8. Snapshot illustrating m^{3}A docking into the AlkA substrate-binding pocket. The m^{3}A base analog is positioned in the middle of the figure, stacking towards the Trp272 side chain. The side chain of the catalytically essential Asp238 residue is positioned to attack the C1–N1 bond.

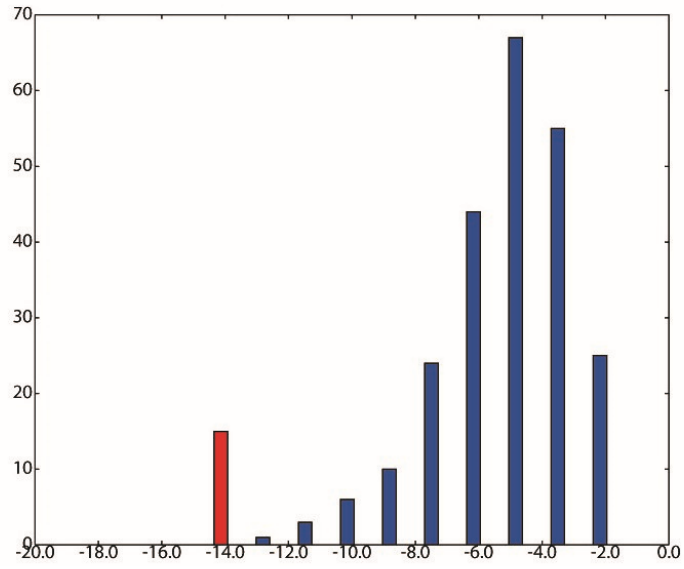

Supplementary Figure S9. Docking energy distribution for 250 docking attempts of a trinucleotide containing fU. Most ligand conformations in the lowest energy bin have the fU base analog bound in the substrate-binding pocket, forming reasonable interactions with the protein.

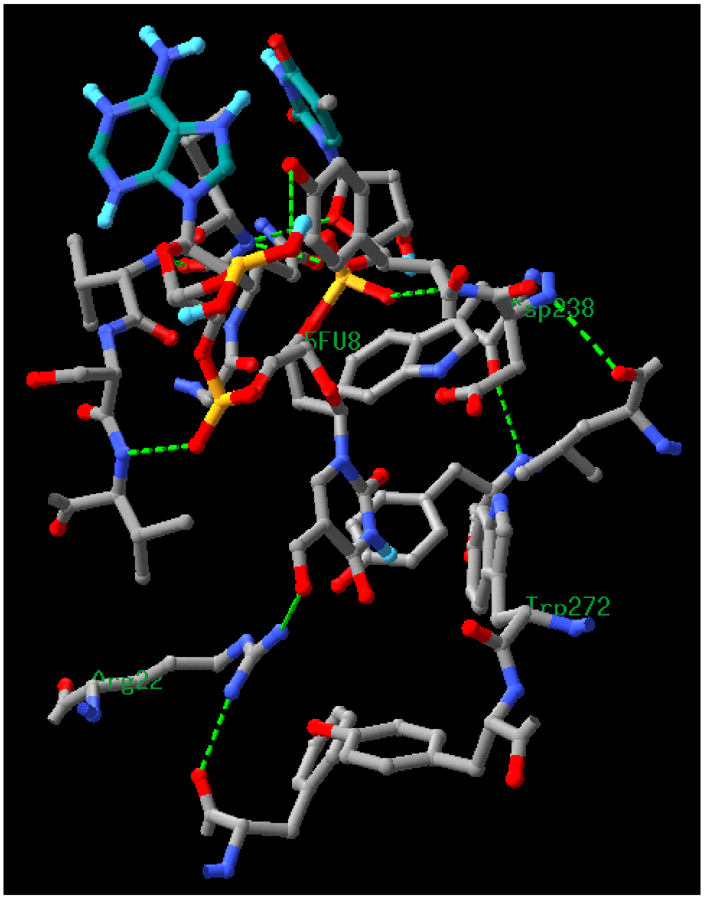

Supplementary Figure S10. Snapshot illustrating fU docking. Unlike m^3A docking, no stacking is formed to Trp272, but instead the fU formyl group forms a hydrogen bond with Arg22. Similar to m^3A docking, Asp238 is positioned to attack the C1–N1 bond.

Supplementary Table S1. Final cell number of wild-type, *alkA*⁻, and *xth*⁻ *nfo*⁻ mutants without and with fdU in the culture medium. A, primary culture selection (N_t = 1.5–1.8±0.5 × 10^9 cells); B, secondary culture selection (N_t = 0.6–0.7±0.2 × 10^9 cells)

A

fdU	N_t (× 10^9)		Time (h)		*C*
(mM)	Average ± SD	Median	Average± SD	Median	
	Wild-type				
0	1.8 ± 0.5	1.8	38 ± 18	41	241
0.1	1.6 ± 0.5	1.5	39 ± 11	44	68
0.2	1.6 ± 0.5	1.6	39 ± 12	45	59
	alkA⁻				
0	1.8 ± 0.5	1.8	37 ± 15	42	189
0.1	1.5 ± 0.5	1.4	49 ± 16	45	57
0.2	1.6 ± 0.6	1.3	43 ± 9	46	59
	xth⁻ *nfo*⁻				
0	1.8 ± 0.6	1.7	47 ± 8	48	105
0.1	1.5 ± 0.4	1.5	48 ± 9	47	58
0.2	1.7 ± 0.5	1.5	47 ± 4	48	59

B

fdU	N_t (× 10^9)		Time (h)		C
(mM)	Average ± SD	Median	Average ± SD	Median	
	Wild-type				
0	0.6 ± 0.1	0.7	56 ± 28	48	41
0.1	0.6 ± 0.2	0.6	44 ± 18	45	22
0.2	0.6 ± 0.2	0.6	53 ± 18	46	36
	alkA⁻				
0	0.6 ± 0.2	0.6	45 ± 18	46	26
0.1	0.6 ± 0.2	0.6	46 ± 8	46	23
0.2	0.7 ± 0.2	0.6	48 ± 4	47	33

N_t, final number of viable cells per 2 mL cell culture after the approximate incubation time indicated; C, number of cultures.

Supplementary Table S2. Growth and mutagenesis experiment parameters. A, primary culture selection (N_t = 1.5–1.8±0.5 × 10^9 cells); B, secondary culture selection (N_t = 0.6–0.7±0.2 × 10^9 cells).

A

fdU	Wild-type				*alkA*⁻				*xth*⁻ *nfo*⁻			
(mM)	*C*	N_t	p_0	m_{act}	*C*	N_t	p_0	m_{act}	*C*	N_t	p_0	m_{act}
0	241	1.8	0.307	2.29	189	1.8	0.28	2.46	105	1.7	0.305	2.30
0.1	68	1.5	0.162	3.53	57	1.4	0.123	4.06	58	1.5	0.172	3.41
0.2	59	1.6	0.254	2.65	59	1.3	0.169	3.44	59	1.5	0.153	3.64

The mutation rates are presented in **Table 1A**.

B

fdU	Wild-type				*alkA*⁻			
(mM)	*C*	N_t	p_0	m_{act}	*C*	N_t	p_0	m_{act}
0	41	0.7	0.439	1.60	26	0.6	0.654	0.823
0.1	22	0.6	0.182	3.30	23	0.6	0.261	2.60
0.2	36	0.6	0.528	1.24	33	0.6	0.340	1.96

The mutation rates are presented in **Table 1B**.

C, number of cultures; N_t, median viable cells (× 10^9) per 2 mL mutagenesis culture; p_0, proportion of cultures without mutants; m_{act}, the actual number of mutations per culture following estimation from the observed number of mutations per culture (m_{obs}) using the equation $m_{act} = m_{obs} (z - 1)/z \ln(z)$, where *z* is the fraction of culture plated (see *Mutagenesis Experiments* for details).

Supplementary Table S3. AlkA purification.

Fraction	Volume (mL)	Protein (mg)	Protein concentration (mg/mL)
Cell extract	40	198	4.948
Soluble protein	40	74	1.851
Gel filtration	18	6.2	0.343
Cation exchange chromatography	3.5	1.9	0.541

Garibyan, L., Huang, T., Kim, M., Wolff, E., Nguyen, A., Nguyen, T., Diep, A., Hu, K., Iverson, A., Yang, H., and Miller, J.H. (2003). Use of the *rpoB* gene to determine the specificity of base substitution mutations on the *Escherichia coli* chromosome. *DNA Repair (Amst)* 2**,** 593–608.

Hollis, T., Ichikawa, Y., and Ellenberger, T. (2000). DNA bending and a flip-out mechanism for base excision by the helix-hairpin-helix DNA glycosylase, *Escherichia coli* AlkA. *EMBO J* 19**,** 758–766.

Leiros, I., Nabong, M.P., Grøsvik, K., Ringvoll, J., Haugland, G.T., Uldal, L., Reite, K., Olsbu, I.K., Knævelsrud, I., Moe, E., Andersen, O.A., Birkeland, N.-K., Ruoff, P., Klungland, A., and Bjelland, S. (2007). Structural basis for enzymatic excision of N^1-methyladenine and N^3-methylcytosine from DNA. *EMBO J* 26**,** 2206–2217.
