# Supplementary material for: The Escherichia coli alkA Gene Is Activated to Alleviate Mutagenesis by an Oxidized Deoxynucleoside
Source: Front Microbiol. 2020 Feb 25;11:263. doi: 10.3389/fmicb.2020.00263 (PMC7051996; doi:10.3389/fmicb.2020.00263)
Supplement: Supplementary file 2 [file Data_Sheet_2.pdf]

Mutagenesis Experiments

Primary Selection: N_t = 1.5–1.8±0.5 × 10^9 cells

Wild-type

0 mM fdU (spontaneous mutagenesis)

241 experiments selected from totally 313 experiments

Culture	N_0	Time (h)	N_t	Mutants/plate							z	r	N_0/N_t
				1	2	3	4	5	6	7			
wt0-1	9000	24.25	2034000000	0	0	0	0	0	0		0.30	0	0.000004
wt0-2	9000	24.25	2800000000	1	0	2	1	1	1		0.30	20	0.000003
wt0-3	9000	24.25	2540000000	0	0	0	0	1	0		0.30	3	0.000004
wt0-4	9000	24.25	2410000000	0	2	0	1	0	0		0.30	10	0.000004
wt0-5	9000	24.25	2434000000	0	0	0	0	0	1		0.30	3	0.000004
wt0-6	9000	24.25	1920000000	0	0	0	1	0	0		0.30	3	0.000005
wt0-7	9000	25	2410000000	1	0	2	1	3	1		0.30	27	0.000004
wt0-8	9000	25	3080000000	1	1	0	1	1	1		0.30	17	0.000003
wt0-9	9000	25	2694000000	0	2	3	1	1	0		0.30	23	0.000003
wt0-10	9000	25	2980000000	5	3	4	1	1	3		0.30	57	0.000003
wt0-11	9000	20.28	1420000000	0	0	0	0	0	1		0.30	3	0.000006
wt0-12	9000	43.25	1570000000	2	0	0	0	0	1		0.30	10	0.000006
wt0-13	18000	49.67	1140000000	0	0	0	0	0	0		0.30	0	0.00002
wt0-14	9000	43.25	1310000000	1	0	0	0	0	0		0.30	3	0.000007
wt0-15	18000	89.75	1910000000	1	0	0	0	0	0		0.30	3	0.000009
wt0-16	18000	89.75	1850000000	0	1	0	0	0	0		0.30	3	0.00001
wt0-17	18000	42.75	2390000000	0	0	0	0	0	0		0.30	0	0.000008
wt0-18	18000	42.75	2690000000	0	0	3	0	3	2		0.30	27	0.000007
wt0-19	18000	42.75	3000000000	1	0	1	0	0	1		0.30	10	0.000006
wt0-20	9000	114.42	1660000000	1	0	0	1	0	0		0.30	7	0.000005
wt0-21	9000	20	1240000000	0	1	0	0	0	0		0.30	3	0.000007
wt0-22	9000	20	1870000000	2	1	0	2	2	3		0.30	33	0.000005
wt0-23	9000	20	1370000000	1	0	0	0	0	0		0.30	3	0.000007
wt0-24	9000	20	1180000000	2	2	0	0	0	1		0.30	17	0.000008
wt0-25	9000	20	2120000000	0	0	0	0	0	0		0.30	0	0.000004
wt0-26	9000	20	1630000000	5	2	0	1	4	2		0.30	47	0.000006
wt0-27	9000	20	1080000000	2	3	0	4	0	3		0.30	40	0.000008
wt0-28	9000	20	1170000000	2	1	2	0	2	1		0.30	27	0.000008
wt0-29	9000	23.5	2060000000	3	1	1	0	0	0		0.30	17	0.000004
wt0-30	9000	23.5	1570000000	2	1	0	0	0	0		0.30	10	0.000006
wt0-31	9000	44.75	2070000000	1	1	1	1	4	3		0.30	37	0.000004
wt0-32	9000	44.75	1880000000	2	0	0	0	0	0		0.30	7	0.000005
wt0-33	9000	23.5	1860000000	1	3	0	0	0	0		0.30	13	0.000005
wt0-34	9000	44.75	2190000000	4	4	4	2	0	0		0.30	47	0.000004
wt0-35	9000	23.5	1900000000	1	2	1	0	0	0		0.30	13	0.000005
wt0-36	9000	44.75	1910000000	0	0	0	0	0	0		0.30	0	0.000005
wt0-37	9000	44.75	1470000000	4	1	1	5	2	0		0.30	43	0.000006
wt0-38	9000	40.75	1070000000	1	0	0	0	0	0		0.30	3	0.000008
wt0-39	9000	43	1780000000	0	0	0	0	0	0		0.30	0	0.000005
wt0-40	9000	43	1010000000	1	0	0	0	0	0		0.30	3	0.000009
wt0-41	9000	43	1180000000	0	0	0	0	1	1		0.30	7	0.000008
wt0-42	9000	43	1660000000	1	1	1	0	0	0		0.30	10	0.000005

wt0-43	9000	46.5	2020000000	1	2	2	0	0	0	0.30	17	0.000005
wt0-44	9000	46.5	1650000000	0	0	0	0	0	0	0.30	0	0.000006
wt0-45	9000	46.5	1270000000	0	0	0	0	0	0	0.30	0	0.000007
wt0-46	9000	46.5	940000000	2	2	6	2	0	0	0.30	40	0.00001
wt0-47	9000	46.5	1760000000	0	0	0	0	0	0	0.30	0	0.000005
wt0-48	9000	26.25	2050000000	3	10	6	9	11	9	0.30	160	0.000004
wt0-49	9000	26.25	2400000000	0	0	0	0	0	0	0.30	0	0.000004
wt0-50	9000	26.25	2350000000	0	0	0	0	0	0	0.30	0	0.000004
wt0-51	9000	26.25	2360000000	0	1	0	1	0	0	0.30	7	0.000004
wt0-52	9000	26.25	2580000000	0	0	0	0	0	0	0.30	0	0.000004
wt0-53	9000	26.25	1540000000	0	0	0	0	0	0	0.30	0	0.000006
wt0-54	9000	26.25	1690000000	0	0	0	0	0	0	0.30	0	0.000005
wt0-55	9000	26.25	1530000000	0	0	0	0	0	0	0.30	0	0.000006
wt0-56	9000	26.25	1450000000	0	0	0	0	0	0	0.30	0	0.000006
wt0-57	9000	26.25	1590000000	0	2	1	2	0	0	0.30	17	0.000006
wt0-58	9000	41.25	1470000000	1	1	1	1	1	1	0.30	20	0.000006
wt0-59	9000	41.25	2000000000	1	0	0	0	0	0	0.30	3	0.000005
wt0-60	9000	41.25	1720000000	2	0	0	0	0		0.25	8	0.000005
wt0-61	9000	41.25	2060000000	1	1	0	0	0	0	0.30	7	0.000004
wt0-62	9000	41.25	1460000000	6	1	1	4	3	5	0.30	67	0.000006
wt0-63	9000	41.25	1990000000	1	0	0	0	0	0	0.30	3	0.000005
wt0-64	9000	41.25	1950000000	4	1	6	4	4	2	0.30	70	0.000005
wt0-65	9000	41.25	1900000000	1	0	0	0	0	0	0.30	3	0.000005
wt0-66	9000	41.25	2670000000	1	1	1	0	0	0	0.30	10	0.000003
wt0-67	9000	41.25	2100000000	0	0	0	0	0	0	0.30	0	0.000004
wt0-68	9000	64.5	2190000000	0	0	0	0	0	0	0.30	0	0.000004
wt0-69	9000	64.5	1950000000	2	1	0	0	0	0	0.30	10	0.000005
wt0-70	9000	64.5	1990000000	1	1	1	0	0	0	0.30	10	0.000005
wt0-71	9000	64.5	2110000000	1	7	5	4	2	0	0.30	63	0.000004
wt0-72	9000	64.5	2570000000	0	0	0	0	0	0	0.30	0	0.000004
wt0-73	9000	64.5	910000000	0	0	0	0	0	0	0.30	0	0.00001
wt0-74	9000	64.5	1220000000	0	0	0	0	0	0	0.30	0	0.000007
wt0-75	9000	64.5	940000000	0	0	0	0	0	0	0.30	0	0.00001
wt0-76	45000	46.58	2390000000	3	3	6	3	4	8	0.30	90	0.00002
wt0-77	45000	46.58	2600000000	0	1	0	2	0	0	0.30	10	0.00002
wt0-78	45000	46.58	2410000000	0	0	0	0	0	1	0.30	3	0.00002
wt0-79	45000	46.58	2320000000	0	0	0	0	0	1	0.30	3	0.00002
wt0-80	45000	46.58	1040000000	3	4	1	1	7	4	0.30	67	0.00004
wt0-81	45000	46.58	2160000000	1	0	2	1	1	0	0.30	17	0.00002
wt0-82	45000	56.00	1980000000	2	0	1	1	1	4	0.30	30	0.00002
wt0-83	45000	56.00	1470000000	0	0	1	2	0	0	0.30	10	0.00003
wt0-84	45000	46.15	1530000000	2	5	3	3	0		0.25	52	0.00003
wt0-85	45000	46.15	1930000000	0	0	0	0	0		0.25	0	0.00002
wt0-86	45000	46.15	1590000000	0	0	0	0	0		0.25	0	0.00003
wt0-87	45000	46.15	1610000000	1	0	0	1	0		0.25	8	0.00003
wt0-88	45000	46.15	1410000000	0	0	0	0	0		0.25	0	0.00003
wt0-89	45000	23.5	1940000000	0	0	0	0	0	0	0.30	0	0.00002
wt0-90	45000	23.5	1520000000	0	0	0	0	0	0	0.30	0	0.00003
wt0-91	45000	23.5	1750000000	0	0	1	1	0	0	0.30	7	0.00003
wt0-92	45000	23.5	1800000000	1	0	1	2	0	0	0.30	13	0.00003
wt0-93	45000	23.5	1700000000	2	0	2	1	0	1	0.30	20	0.00003
wt0-94	45000	23.5	1980000000	1	2	0	1	0	1	0.30	17	0.00002
wt0-95	45000	22.42	2270000000	0	0	0	0	2	0	0.30	7	0.00002
wt0-96	45000	22.42	1850000000	5	2	4	4	3	2	0.30	67	0.00002
wt0-97	45000	22.42	1750000000	0	0	0	0	0	0	0.30	0	0.00003

wt0-98	45000	22.42	1890000000	5	0	3	1	0	3	0.30	40	0.00002
wt0-99	45000	22.42	1840000000	0	0	0	0	0	0	0.30	0	0.00002
wt0-100	45000	22.42	1440000000	0	0	1	0	0	0	0.30	3	0.00003
wt0-101	45000	22.5	1870000000	1	0	2	2	0	0	0.30	17	0.00002
wt0-102	45000	22.5	1940000000	2	0	1	1	0	0	0.30	13	0.00002
wt0-103	45000	22.5	1610000000	0	1	1	1	1	3	0.30	23	0.00003
wt0-104	45000	22.5	1720000000	3	3	3	4	6	4	0.30	77	0.00003
wt0-105	45000	22.5	1730000000	12	9	3	7	15	3	0.30	163	0.00003
wt0-106	45000	22.5	1460000000	0	1	2	1	1	2	0.30	23	0.00003
wt0-107	45000	22.58	2100000000	5	12	3	3	3	10	0.30	120	0.00002
wt0-108	45000	22.58	2220000000	1	1	1	3	2	1	0.30	30	0.00002
wt0-109	45000	22.58	2010000000	0	0	0	2	2	1	0.30	17	0.00002
wt0-110	45000	22.58	2010000000	5	4	8	6	5	12	0.30	133	0.00002
wt0-111	45000	22.58	1740000000	1	1	0	0	0	0	0.30	7	0.00003
wt0-112	45000	20.92	2480000000	8	8	4	12	10		0.25	168	0.00002
wt0-113	45000	20.92	2030000000	0	0	0	1	1		0.25	8	0.00002
wt0-114	45000	20.92	1410000000	0	0	1	1	2		0.25	16	0.00003
wt0-115	45000	20.92	1980000000	4	9	3	9	6		0.25	124	0.00002
wt0-116	45000	20.92	1480000000	14	42	29	58	49		0.25	768	0.00003
wt0-117	45000	22.42	1520000000	0	1	0	0	0		0.25	4	0.00003
wt0-118	45000	22.42	950000000	83	95	94	94	75		0.25	1764	0.00005
wt0-119	45000	22.42	1020000000	5	1	6	3	0		0.25	60	0.00004
wt0-120	45000	22.42	1880000000	4	10	8	8	9		0.25	156	0.00002
wt0-121	9000	21.75	1890000000	0	0	0	0	1	1	0.30	7	0.000005
wt0-122	9000	21.75	2830000000	0	0	1	1	0	0	0.30	7	0.000003
wt0-123	9000	21.75	2950000000	0	0	0	0	0	0	0.30	0	0.000003
wt0-124	9000	21.75	3090000000	0	0	0	0	0	0	0.30	0	0.000003
wt0-125	9000	21.75	2420000000	0	0	0	0	0	1	0.30	3	0.000004
wt0-126	9000	19.67	1730000000	3	3	5	2	5	0	0.30	60	0.000005
wt0-127	9000	19.67	1860000000	1	0	0	0	0	0	0.30	3	0.000005
wt0-128	9000	19.67	2760000000	1	0	0	0	0	0	0.30	3	0.000003
wt0-129	9000	19.67	2430000000	0	0	0	0	0	0	0.30	0	0.000004
wt0-130	9000	19.67	2740000000	0	0	0	0	0	0	0.30	0	0.000003
wt0-131	9000	23.83	1110000000	0	0	0	0	0	0	0.30	0	0.000008
wt0-132	9000	23.83	2360000000	0	0	0	1	1	0	0.30	7	0.000004
wt0-133	9000	15.17	2320000000	1	2	0	0	0	0	0.30	10	0.000004
wt0-134	9000	15.17	2746000000	0	0	0	0	0	0	0.30	0	0.000003
wt0-135	9000	15.17	2754000000	1	1	0	0	0	0	0.30	7	0.000003
wt0-136	9000	18.08	2270000000	1	0	0	0	0	0	0.30	3	0.000004
wt0-137	9000	18.08	1560000000	0	3	0	0	0	0	0.30	10	0.000006
wt0-138	9000	18.08	1730000000	1	0	0	0	1	0	0.30	7	0.000005
wt0-139	9000	18.08	1790000000	1	0	2	2	1	0	0.30	20	0.000005
wt0-140	9000	19	2460000000	0	1	0	2	0	2	0.30	17	0.000004
wt0-141	9000	19	2060000000	1	1	0	0	0	0	0.30	7	0.000004
wt0-142	9000	19	2106000000	0	0	0	0	0	0	0.30	0	0.000004
wt0-143	9000	19	2106000000	0	0	0	0	0	0	0.30	0	0.000004
wt0-144	9000	23.25	1850000000	1	0	0	1	1	0	0.30	10	0.000005
wt0-145	9000	23.25	1854000000	0	0	0	0	0	0	0.30	0	0.000005
wt0-146	9000	23.25	1854000000	0	0	0	0	0	0	0.30	0	0.000005
wt0-147	9000	23.25	1920000000	1	0	0	0	0	0	0.30	3	0.000005
wt0-148	9000	23.25	1320000000	0	0	0	0	1	0	0.30	3	0.000007
wt0-149	9000	23	1546000000	0	0	0	0	0	0	0.30	0	0.000006
wt0-150	9000	23	2134000000	2	1	1	2	0	0	0.30	20	0.000004
wt0-151	9000	23	1934000000	0	0	0	0	0	0	0.30	0	0.000005
wt0-152	9000	23	1626000000	0	0	1	0	1	1	0.30	10	0.000006

wt0-153	9000	23	2314000000	0	0	0	0	0	0		0.30	0	0.000004
wt0-154	9000	20.42	2320000000	1	2	9	7	8	6		0.30	110	0.000004
wt0-155	9000	20.42	2720000000	1	0	0	0	0	0		0.30	3	0.000003
wt0-156	9000	20.42	2920000000	1	0	0	0	0	0		0.30	3	0.000003
wt0-157	9000	20.42	1750000000	0	0	0	0	0	0		0.30	0	0.000005
wt0-158	9000	23.08	1413400000	1	0	1	0	2	0		0.30	13	0.000006
wt0-159	9000	23.08	1800000000	5	2	1	2	1	3		0.30	47	0.000005
wt0-160	9000	23.08	2814000000	0	0	11	3	3	7		0.30	80	0.000003
wt0-161	9000	23.08	1406000000	1	1	0	0	0	0		0.30	7	0.000006
wt0-162	9000	23.08	1300000000	6	26	6	4	8	5		0.30	183	0.000007
wt0-163	9000	20.25	1700000000	1	0	0	0	0	0		0.30	3	0.000005
wt0-164	9000	20.25	2320000000	2	0	0	1	0	1		0.30	13	0.000004
wt0-165	9000	20.25	1820000000	3	1	1	1	0	0		0.30	20	0.000005
wt0-166	9000	20.25	2226000000	0	1	0	0	0	0		0.30	3	0.000004
wt0-167	9000	45.5	1516000000	0	0	0	0	0	0		0.30	0	0.000006
wt0-168	9000	45.5	1331000000	0	0	0	0	0	0		0.30	0	0.000007
wt0-169	9000	41.25	1633333333	0	0	0	0	0	0		0.30	0	0.000006
wt0-170	9000	44.5	2240000000	0	0	0	0	0	0		0.30	0	0.000004
wt0-171	9000	44.5	1486666667	1	1	2	6	2	1		0.30	43	0.000006
wt0-172	9000	48	1604000000	0	1	0	0	1	0		0.30	7	0.000006
wt0-173	9000	48	1670000000	0	1	0	0	1	0		0.30	7	0.000005
wt0-174	9000	48	2914666667	0	0	0	0	0	0		0.30	0	0.000003
wt0-175	9000	48	1994666667	0	0	0	0	0	0		0.30	0	0.000005
wt0-176	9000	44	2526666667	0	0	1	0	0	0		0.30	3	0.000004
wt0-177	9000	44	1613333333	0	0	0	0	0	0		0.30	0	0.000006
wt0-178	9000	45	1768000000	0	2	1	1	1	2		0.30	23	0.000005
wt0-179	9000	45	1688000000	0	0	0	0	0	0		0.30	0	0.000005
wt0-180	9000	49.75	957000000	1	1	2	1	1	0	6	0.35	34	0.000009
wt0-181	9000	44	970000000	0	0	0	0	0	0		0.30	0	0.000009
wt0-182	9000	44	1273333333	0	0	0	0	0	0		0.30	0	0.000007
wt0-183	9000	44	1620000000	0	0	0	0	0	0		0.30	0	0.000006
wt0-184	9000	43	1440000000	0	0	0	0	0	0		0.30	0	0.000006
wt0-185	9000	43	910666667	0	0	0	0	0	0		0.30	0	0.00001
wt0-186	9000	43	923000000	0	0	0	0	0	0		0.30	0	0.00001
wt0-187	9000	45.17	1179000000	0	0	0	0	0	0		0.30	0	0.000008
wt0-188	9000	46.25	1505500000	0	0	0	0	1	0		0.30	3	0.000006
wt0-189	9000	144	1010000000	0	0	0	0	0	0		0.30	0	0.000009
wt0-190	9000	72	1233333333	0	0	0	0	0	0		0.30	0	0.000007
wt0-191	9000	144	1006666667	0	0	0	0	0	0		0.30	0	0.000009
wt0-192	9000	48	1406666667	1	0	1	0	0	0		0.30	7	0.000006
wt0-193	9000	48	1220000000	3	3	1	3	5	2		0.30	57	0.000007
wt0-194	9000	48	1233333333	0	0	0	0	0	0		0.30	0	0.000007
wt0-195	9000	48	1010000000	0	0	0	0	0	0		0.30	0	0.000009
wt0-196	9000	48	1366666667	0	0	0	0	0	0		0.30	0	0.000007
wt0-197	9000	48	1153333333	0	1	0	0	0	0		0.30	3	0.000008
wt0-198	9000	48	1346666667	0	0	0	0	0	0		0.30	0	0.000007
wt0-199	9000	48	1273333333	0	0	0	0	0	0		0.30	0	0.000007
wt0-200	9000	48	1420000000	0	0	0	0	0	0		0.30	0	0.000006
wt0-201	9000	48	1313333333	0	0	0	0	0	0		0.30	0	0.000007
wt0-202	9000	48	1270000000	0	0	0	0	0	0		0.30	0	0.000007
wt0-203	9000	48	1266666667	0	0	0	0	0	0		0.30	0	0.000007
wt0-204	9000	48	1050000000	0	0	0	1	0	0		0.30	3	0.000009
wt0-205	9000	48	1280000000	5	5	6	4	9	10		0.30	130	0.000007
wt0-206	9000	48	1130000000	0	0	0	0	0	0		0.30	0	0.000008
wt0-207	9000	42.5	1480000000	0	0	0	0	0	0		0.30	0	0.000006

wt0-208	9000	42.5	1593333333	1	1	3	2	4	0	0.30	37	0.000006
wt0-209	9000	42.5	1120000000	1	1	0	2	0	0	0.30	13	0.000008
wt0-210	9000	48	1710000000	1	0	2	5	1	0	0.30	30	0.000005
wt0-211	9000	48	1886666667	5	7	8	3	6	3	0.30	107	0.000005
wt0-212	9000	48	1810000000	0	1	1	1	0	3	0.30	20	0.000005
wt0-213	9000	48	1693333333	2	2	0	1	2	1	0.30	27	0.000005
wt0-214	9000	48	1540000000	0	2	4	1	0	0	0.30	23	0.000006
wt0-215	9000	68.75	1390000000	1	0	2	0	0	1	0.30	13	0.000007
wt0-216	9000	68.75	1360000000	0	0	2	0	0	0	0.30	7	0.000007
wt0-217	9000	68.75	1740000000	1	5	2	1	4	4	0.30	57	0.000005
wt0-218	9000	68.75	1530000000	1	2	2	0	1	1	0.30	23	0.000006
wt0-219	9000	68.75	1590000000	1	1	2	0	0	1	0.30	17	0.000006
wt0-220	9000	68.75	1470000000	1	1	0	1	1	1	0.30	17	0.000006
wt0-221	9000	46	1553333333	3	11	21	10	28	25	0.30	327	0.000006
wt0-222	9000	46	1193333333	0	0	0	0	0	0	0.30	0	0.000008
wt0-223	9000	46	1726666667	3	2	4	7	7	2	0.30	83	0.000005
wt0-224	9000	46	2940000000	0	1	0	0	1	2	0.30	13	0.000003
wt0-225	9000	46	2630000000	3	1	1	5	0	0	0.30	33	0.000003
wt0-226	9000	46	2940000000	0	0	0	0	0	1	0.30	3	0.000003
wt0-227	9000	46	1690000000	0	0	0	0	0	0	0.30	0	0.000005
wt0-228	9000	46	2500000000	1	7	3	3	6	1	0.30	70	0.000004
wt0-229	9000	46	2160000000	1	0	0	5	4	2	0.30	40	0.000004
wt0-230	9000	46	2786666667	0	1	0	3	2	3	0.30	30	0.000003
wt0-231	9000	46	1966666667	0	0	1	0	0	0	0.30	3	0.000005
wt0-232	9000	46	1573333333	0	4	1	2	1	1	0.30	30	0.000006
wt0-233	9000	48	1980000000	2	2	1	0	0	1	0.30	20	0.000005
wt0-234	9000	48	2400000000	1	0	0	0	0	0	0.30	3	0.000004
wt0-235	9000	48	2260000000	0	1	1	2	1	0	0.30	17	0.000004
wt0-236	9000	48	2206666667	0	1	0	0	0	0	0.30	3	0.000004
wt0-237	9000	48	2100000000	0	5	6	0	1	0	0.30	40	0.000004
wt0-238	9000	48	2120000000	0	1	0	0	0	0	0.30	3	0.000004
wt0-239	9000	46	2126666667	1	0	2	1	0	2	0.30	20	0.000004
wt0-240	9000	46	2160000000	0	2	0	0	0	0	0.30	7	0.000004
wt0-241	9000	46	1930000000	0	1	6	1	0	1	0.30	30	0.000005
Average	15946	38	1833057676									0.000009
SD	14021	18	513364369									0.000009
Median	9000	41	1800000000									0.000006
Average			1.8×10^9									
SD			0.5×10^9									
Median			1.8×10^9									

zeros = 74

C = 241

$p_0 = 0.307$

$m_{obs} = 1.18$

$m_{act} = 2.29$

$\mu = 1.27 \times 10^{-9}$

	%	μ, $\times 10^{-9}$
AT→GC	14	0.177
GC→AT	35	0.443
GC→TA	14	0.177
AT→CG	12	0.148
AT→TA	10	0.133
GC→CG	3.5	0.0443
BS	88	1.12
Indels	2.3	0.0296
Unknown	9.3	0.118
**Total**	100	1.27

0.1 mM fdU

68 experiments selected from totally 140 experiments

Culture	N_0	Time (h)	N_t	Mutants/plate							z	r	N_0/N_t
				1	2	3	4	5	6	7			
wt0.1-1	9000	46.58	2426000000	5	14	17	10	18	16		0.3	267	0.000004
wt0.1-2	9000	46.58	2954000000	13	15	11	15	17	18		0.3	297	0.000003
wt0.1-3	9000	46.58	2300000000	10	12	9	14	14	13		0.3	240	0.000004
wt0.1-4	9000	46.58	2246000000	28	26	23	35	34	48		0.3	647	0.000004
wt0.1-5	9000	46.58	2066000000	160	129	165	166	181	163		0.3	3213	0.000004
wt0.1-6	9000	46.58	2340000000	289	285	287	296	322	330		0.3	6030	0.000004
wt0.1-7	9000	47.33	1230000000	9	9	10	5	9	4		0.3	153	0.000007
wt0.1-8	9000	47.33	2140000000	3	4	5	4	0	3		0.3	63	0.000004
wt0.1-9	9000	47.33	1450000000	47	40	51	44	46	36		0.3	880	0.000006
wt0.1-10	9000	47.33	1930000000	12	18	17	17	12	14		0.3	300	0.000005
wt0.1-11	9000	43.25	1110000000	0	0	0	0	0	0		0.3	0	0.000008
wt0.1-12	18000	22.17	1170000000	0	1	0	1	0	0		0.3	7	0.00002
wt0.1-13	18000	49.67	1100000000	4	8	7	6	11	4		0.3	133	0.00002
wt0.1-14	18000	49.67	1050000000	0	6	2	2	3	3		0.3	53	0.00002
wt0.1-15	18000	42.75	1840000000	1	0	0	1	0	2		0.3	13	0.00001
wt0.1-16	18000	42.75	1720000000	1	1	7	8	4	3		0.3	80	0.00001
wt0.1-17	18000	42.75	1720000000	0	1	2	1	2	1		0.3	23	0.00001
wt0.1-18	18000	42.75	1720000000	0	0	1	1	0	0		0.3	7	0.00001
wt0.1-19	9000	20	1290000000	0	2	1	1	0	1		0.3	17	0.000007
wt0.1-20	9000	20	1040000000	1	3	0	1	1	1		0.3	23	0.000009
wt0.1-21	9000	20	1520000000	0	2	1	0	1	3		0.3	23	0.000006
wt0.1-22	9000	20	1130000000	0	0	1	0	0	0		0.3	3	0.000008
wt0.1-23	9000	20	1140000000	0	0	2	1	2	3		0.3	27	0.000008
wt0.1-24	9000	20	1300000000	0	3	1	0	1	1		0.3	20	0.000007
wt0.1-25	9000	20	1390000000	2	2	2	1	3	3		0.3	43	0.000007
wt0.1-26	9000	20	1000000000	1	2	1	0	1	1		0.3	20	0.000009
wt0.1-27	9000	20	1450000000	13	6	9	6	10	6		0.3	167	0.000006
wt0.1-28	9000	23.5	1290000000	2	4	1	1	0	0		0.3	27	0.000007
wt0.1-29	9000	23.5	1300000000	1	1	2	2	2	4		0.3	40	0.000007
wt0.1-30	9000	44.75	1060000000	1	1	3	1	4	0		0.3	33	0.000009
wt0.1-31	9000	44.75	1390000000	1	1	1	1	1	0		0.3	17	0.000007
wt0.1-32	9000	23.5	1470000000	1	1	2	1	1	1		0.3	23	0.000006
wt0.1-33	9000	44.75	1350000000	2	2	2	1	0	0		0.3	23	0.000007
wt0.1-34	9000	44.75	900000000	2	1	2	4	3	1		0.3	43	0.00001
wt0.1-35	45000	46.58	1750000000	5	0	11	3	3	1		0.3	77	0.00003
wt0.1-36	45000	46.58	1550000000	268	263	256	241	237	220		0.3	4950	0.00003
wt0.1-37	45000	46.58	1750000000	1	0	0	1	2	0		0.3	13	0.00003
wt0.1-38	45000	46.58	930000000	26	29	27	24	34	30		0.3	567	0.00005
wt0.1-39	45000	46.58	2250000000	1	2	0	0	0	2		0.3	17	0.00002
wt0.1-40	45000	22.42	1870000000	8	0	0	0	0	0		0.3	27	0.00002
wt0.1-41	45000	22.42	1750000000	0	4	0	6	0	15		0.3	83	0.00003
wt0.1-42	45000	22.42	2020000000	0	0	0	19	0	15		0.3	113	0.00002
wt0.1-43	45000	22.42	1680000000	13	15	16	11	13	26		0.3	313	0.00003
wt0.1-44	45000	22.42	1580000000	0	2	0	1	0	0		0.3	10	0.00003
wt0.1-45	45000	22.42	1970000000	11	10	7	7	8	0		0.3	143	0.00002
wt0.1-46	9000	45.5	1336000000	0	0	0	0	0	0		0.3	0	0.000007
wt0.1-47	9000	45.5	1497000000	0	0	0	0	0	0		0.3	0	0.000006
wt0.1-48	9000	45.5	1477000000	0	0	0	0	0	0		0.3	0	0.000006
wt0.1-49	9000	45.5	1081000000	0	0	0	0	0	0		0.3	0	0.000008
wt0.1-50	9000	45.5	1145000000	0	0	0	1	0	0		0.3	3	0.000008
wt0.1-51	9000	47.5	1090000000	0	0	0	0	0	0		0.3	0	0.000008

wt0.1-52	9000	47.5	1438000000	0	0	0	0	0	0		0.3	0	0.000006
wt0.1-53	9000	47.5	1340000000	0	0	0	0	0	0		0.3	0	0.000007
wt0.1-54	9000	41.25	1060000000	0	0	0	0	0	0		0.3	0	0.000009
wt0.1-55	9000	41.25	1190000000	0	0	0	0	0	0		0.3	0	0.000008
wt0.1-56	9000	41.25	1110000000	0	3	2	1	0	0		0.3	20	0.000008
wt0.1-57	9000	44	1281333333	0	0	0	0	2	0		0.3	7	0.000007
wt0.1-58	9000	44	951333333	1	6	3	5	2	1		0.3	60	0.00001
wt0.1-59	9000	44	2440000000	0	0	0	1	1	1		0.3	10	0.000004
wt0.1-60	9000	44	2526666667	0	0	1	0	0	0		0.3	3	0.000004
wt0.1-61	9000	44	2200000000	4	4	2	1	5	2		0.3	60	0.000004
wt0.1-62	9000	44	2695000000	6	6	3	8	2	2		0.3	90	0.000003
wt0.1-63	9000	44	2700000000	0	0	0	1	1	0		0.3	7	0.000003
wt0.1-64	9000	44	2515000000	0	0	0	0	0	0		0.3	0	0.000004
wt0.1-65	9000	44	2153333333	1	0	0	0	0	0		0.3	3	0.000004
wt0.1-66	9000	44	1706666667	0	0	0	1	0	0		0.3	3	0.000005
wt0.1-67	9000	44	964666667	1	2	3	0	0	0		0.3	20	0.000009
wt0.1-68	9000	49.75	1617000000	0	1	1	0	0	0	2	0.35	11	0.000006
Average	15750	39	1605102941										0.00001
SD	13229	11	513327283										0.000009
Median	9000	44	1473500000										0.000007
Average			1.6×10^9										
SD			0.5×10^9										
Median			1.5×10^9										

zeros = 11
C = 68
$p_0 = 0.162$
$m_{obs} = 1.82$
$m_{act} = 3.53$
$\mu = 2.40 \times 10^{-9}$

	**%**	**μ, × 10^{-9}**
**AT→GC**	66	1.59
**GC→AT**	12	0.282
**GC→TA**	4.4	0.106
**AT→CG**	2.9	0.0706
**AT→TA**	7.4	0.176
**GC→CG**	0	0
BS	93	2.22
Indels	0	0
Unknown	7.4	0.176
**Total**	100	2.40

0.2 mM fdU

59 experiments selected from totally 131 experiments

**Culture**	**N_0**	**Time (h)**	**N_t**	**Mutants/plate**							**z**	**r**	**N_0/N_t**
				**1**	**2**	**3**	**4**	**5**	**6**	**7**			
wt0.2-1	45000	22.58	1870000000	2	3	1	6	1	2		0.3	50	0.00002
wt0.2-2	45000	22.58	1460000000	5	4	4	4	5	3		0.3	83	0.00003
wt0.2-3	45000	22.58	1930000000	1	2	1	1	4	1		0.3	33	0.00002
wt0.2-4	45000	22.58	2080000000	13	9	15	12	9	9		0.3	223	0.00002
wt0.2-5	45000	22.58	1570000000	28	21	19	20	34	38		0.3	533	0.00003
wt0.2-6	45000	22.58	2220000000	4	2	0	3	4	4		0.3	57	0.00002
wt0.2-7	9000	19	2274000000	13	18	10	21	7	9		0.3	260	0.000004
wt0.2-8	9000	19	2460000000	1	0	2	3	0	0		0.3	20	0.000004
wt0.2-9	9000	19	2194000000	2	3	2	9	3	4		0.3	77	0.000004
wt0.2-10	9000	19	2526000000	0	3	8	5	2	4		0.3	73	0.000004
wt0.2-11	9000	19	1906000000	1	2	2	2	2	3		0.3	40	0.000005
wt0.2-12	9000	23.25	1874000000	2	2	3	2	3	6		0.3	60	0.000005

wt0.2-13	9000	23.25	1150000000	1	6	7	5	4	4	0.3	90	0.000008
wt0.2-14	9000	23.25	1370000000	4	7	9	1	5	2	0.3	93	0.000007
wt0.2-15	9000	23.25	1130000000	3	4	2	13	13	8	0.3	143	0.000008
wt0.2-16	9000	23.25	1090000000	2	4	2	2	1	7	0.3	60	0.000008
wt0.2-17	9000	44.5	2426666667	3	0	1	1	1		0.25	24	0.000004
wt0.2-18	9000	44.5	2020000000	0	0	0	0	0	1	0.3	3	0.000005
wt0.2-19	9000	44.5	2140000000	0	1	1	0	0	0	0.3	7	0.000004
wt0.2-20	9000	44.5	2040000000	0	0	0	0	1	0	0.3	3	0.000004
wt0.2-21	9000	44.5	2460000000	5	2	4	0	0	2	0.3	43	0.000004
wt0.2-22	9000	44.5	1710000000	2	3	2	7	3	3	0.3	67	0.000005
wt0.2-23	9000	44.5	1630000000	0	0	1	0	0	0	0.3	3	0.000006
wt0.2-24	9000	44.5	1833333333	0	0	0	0	0	0	0.3	0	0.000005
wt0.2-25	9000	44.5	1420000000	0	0	1	1	2	1	0.3	17	0.000006
wt0.2-26	9000	44.5	1520000000	1	1	0	1	0	0	0.3	10	0.000006
wt0.2-27	9000	44	900000000	0	3	1	3	0	5	0.3	40	0.00001
wt0.2-28	9000	44	1173333333	0	0	0	1	0	0	0.3	3	0.000008
wt0.2-29	9000	44	1050000000	0	0	0	3	0	0	0.3	10	0.000009
wt0.2-30	9000	44	1470000000	0	0	0	0	0	0	0.3	0	0.000006
wt0.2-31	9000	44	1800000000	0	2	1	2	1	0	0.3	20	0.000005
wt0.2-32	9000	44	1330000000	0	0	1	3	2	4	0.3	33	0.000007
wt0.2-33	9000	44	1220000000	0	1	1	1	0	1	0.3	13	0.000007
wt0.2-34	9000	44	960000000	0	0	1	0	0	0	0.3	3	0.000009
wt0.2-35	9000	44	1016000000	0	0	0	0	0	2	0.3	7	0.000009
wt0.2-36	9000	44	1220000000	0	0	0	0	0	0	0.3	0	0.000007
wt0.2-37	9000	44	1573333333	0	1	0	0	0	0	0.3	3	0.000006
wt0.2-38	9000	44	1180000000	0	0	0	0	1	1	0.3	7	0.000008
wt0.2-39	9000	48.17	1103500000	0	0	1	0	0	0	0.3	3	0.000008
wt0.2-40	9000	48.17	1127000000	0	0	0	1	1	0	0.3	7	0.000008
wt0.2-41	9000	45.17	1570500000	0	0	0	0	0	0	0.3	0	0.000006
wt0.2-42	9000	45.17	1234500000	0	0	0	0	0	0	0.3	0	0.000007
wt0.2-43	9000	45.17	1299500000	0	0	0	0	0	1	0.3	3	0.000007
wt0.2-44	9000	45.17	1322000000	0	0	0	0	0	0	0.3	0	0.000007
wt0.2-45	9000	45.17	1182500000	0	0	0	0	0	0	0.3	0	0.000008
wt0.2-46	9000	45.17	1550500000	0	0	0	0	0	0	0.3	0	0.000006
wt0.2-47	9000	45.17	948000000	0	0	0	0	0	0	0.3	0	0.00001
wt0.2-48	9000	45.17	1392500000	0	0	0	0	0	0	0.3	0	0.000007
wt0.2-49	9000	46.25	1651000000	0	0	0	0	0	0	0.3	0	0.000006
wt0.2-50	9000	46.25	1670000000	0	0	0	0	0	0	0.3	0	0.000005
wt0.2-51	9000	46.25	1056000000	0	0	0	0	0	0	0.3	0	0.000009
wt0.2-52	9000	46.25	1698666667	0	0	0	0	0	0	0.3	0	0.000005
wt0.2-53	9000	68.75	920000000	0	2	1	0	0	0	0.3	10	0.00001
wt0.2-54	9000	69	920000000	0	0	1	0	2	0	0.3	10	0.00001
wt0.2-55	9000	46	1020000000	0	0	0	0	0	0	0.3	0	0.000009
wt0.2-56	9000	46	1846666667	0	2	1	1	1	0	0.3	17	0.000005
wt0.2-57	9000	46	1706666667	0	2	2	2	3	1	0.3	33	0.000005
wt0.2-58	9000	46	1860000000	0	0	1	0	0	2	0.3	10	0.000005
wt0.2-59	9000	46	2100000000	3	3	3	4	5	3	0.3	70	0.000004
Average	12661	39	1565697740									0.000008
SD	10974	12	453912281									0.000006
Median	9000	45	1550500000									0.000007
Average			1.6×10^9									
SD			0.5×10^9									
Median			1.6×10^9									

% **μ, × 10^{-9}**

zeros = 15
C = 59
p_0 = 0.254
m_{obs} = 1.37
m_{act} = 2.65
μ = 1.71 × 10^{-9}

AT→GC	40	0.644
GC→AT	35	0.545
GC→TA	19	0.297
AT→CG	1.8	0.0248
AT→TA	3.5	0.0496
GC→CG	1.8	0.0248
BS	93	1.59
Indels	1.5	0.0248
Unknown	5.8	0.0992
**Total**	100	1.71

***alkA*⁻**

0 mM fdU (spontaneous mutagenesis)

189 experiments selected from totally 258 experiments

Culture	N_0	Time (h)	N_t	Mutants/plate 1	2	3	4	5	6	7	z	r	N_0/N_t
alkA0-1	9000	21.67	2366000000	0	0	0	0	0	0		0.30	0	0.000004
alkA0-2	9000	21.67	1250000000	0	0	0	0	0	0		0.30	0	0.000007
alkA0-3	9000	22	2180000000	0	0	0	1	0	0		0.30	3	0.000004
alkA0-4	9000	25.42	1866000000	0	0	1	0	0	1		0.30	7	0.000005
alkA0-5	9000	24.08	1886000000	0	0	0	1	1	0		0.30	7	0.000005
alkA0-6	9000	24.08	1660000000	2	0	0	0	1	0		0.30	10	0.000005
alkA0-7	9000	21.92	1690000000	1	0	0	0	0	1		0.30	7	0.000005
alkA0-8	9000	21.92	1620000000	1	1	1	1	1	0		0.30	17	0.000006
alkA0-9	9000	68.41	1800000000	0	0	0	0	0	0		0.30	0	0.000005
alkA0-10	9000	68.41	1270000000	0	0	3	0	0	1		0.30	13	0.000007
alkA0-11	9000	68.41	2890000000	3	0	0	1	0	0		0.30	13	0.000003
alkA0-12	9000	68.41	1870000000	0	0	0	0	0	0		0.30	0	0.000005
alkA0-13	9000	68.41	1340000000	0	0	0	0	0	1		0.30	3	0.000007
alkA0-14	9000	40.5	980000000	0	0	0	0	2	1		0.30	10	0.000009
alkA0-15	9000	23	1130000000	0	0	0	0	0	0		0.30	0	0.000008
alkA0-16	9000	23	1510000000	1	0	0	0	0	0		0.30	3	0.000006
alkA0-17	9000	23	1220000000	0	0	0	0	0	0		0.30	0	0.000007
alkA0-18	9000	23	3090000000	0	2	1	0	2	0		0.30	17	0.000003
alkA0-19	9000	23	2080000000	0	0	0	1	0	1		0.30	7	0.000004
alkA0-20	9000	23	1940000000	0	0	0	0	0	0		0.30	0	0.000005
alkA0-21	9000	23	2310000000	1	1	1	0	1	0		0.30	13	0.000004
alkA0-22	9000	23	1670000000	0	1	3	2	2	3		0.30	37	0.000005
alkA0-23	9000	23	2960000000	0	0	0	0	0	0		0.30	0	0.000003
alkA0-24	9000	23	2320000000	0	0	0	0	0	0		0.30	0	0.000004
alkA0-25	9000	41.75	1610000000	0	0	0	0	0	0		0.30	0	0.000006
alkA0-26	9000	26.25	2940000000	1	1	0	0	0	0		0.30	7	0.000003
alkA0-27	9000	45.17	1200000000	1	5	6	6	15	12		0.30	150	0.000008
alkA0-28	9000	25.17	2020000000	1	3	0	0	1	0		0.30	17	0.000005
alkA0-29	9000	25.17	1980000000	3	4	11	2	3	2		0.30	83	0.000005
alkA0-30	9000	25.17	2180000000	0	1	2	1	0	3		0.30	23	0.000004
alkA0-31	9000	45.17	2730000000	0	0	0	0	0	0		0.30	0	0.000003
alkA0-32	9000	45.17	3040000000	1	0	17	0	0	15		0.30	110	0.000003
alkA0-33	9000	45.17	2120000000	0	0	1	0	4	15		0.30	67	0.000004
alkA0-34	9000	26.5	1560000000	9	10	10	9	7	5		0.30	167	0.000006

alkA0-35	9000	26.5	2020000000	0	0	0	0	0	0	0.30	0	0.000005
alkA0-36	9000	26.5	1820000000	0	0	0	0	0	0	0.30	0	0.000005
alkA0-37	9000	26.5	2270000000	0	0	0	0	0	0	0.30	0	0.000004
alkA0-38	9000	26.5	1400000000	0	0	0	0	0	0	0.30	0	0.000006
alkA0-39	9000	26.5	1340000000	0	0	0	0	2	0	0.30	7	0.000007
alkA0-40	9000	26.5	2000000000	1	0	1	0	0	0	0.30	7	0.000005
alkA0-41	9000	42	1040000000	0	0	0	0	0	0	0.30	0	0.000009
alkA0-42	9000	42	1070000000	0	1	0	1	1	0	0.30	10	0.000008
alkA0-43	9000	42	1220000000	45	44	36	46	56	43	0.30	900	0.000007
alkA0-44	9000	42	1470000000	0	0	0	0	0	0	0.30	0	0.000006
alkA0-45	9000	42	1590000000	3	13	4	4	11	11	0.30	153	0.000006
alkA0-46	9000	42	1880000000	1	1	0	1	0	0	0.30	10	0.000005
alkA0-47	9000	23	1690000000	0	0	0	0	0	0	0.30	0	0.000005
alkA0-48	9000	23	2270000000	0	0	0	0	1	0	0.30	3	0.000004
alkA0-49	9000	23	1830000000	4	5	9	3	8	7	0.30	120	0.000005
alkA0-50	9000	23	2200000000	0	0	1	0	0	1	0.30	7	0.000004
alkA0-51	9000	23	1090000000	0	0	1	0	0	1	0.30	7	0.000008
alkA0-52	9000	23	1830000000	0	0	0	0	0	0	0.30	0	0.000005
alkA0-53	9000	23	2120000000	0	0	0	0	0	0	0.30	0	0.000004
alkA0-54	9000	23	2330000000	0	0	0	0	3		0.25	12	0.000004
alkA0-55	9000	23	1360000000	6	5	3	0	1	2	0.30	57	0.000007
alkA0-56	9000	23	2560000000	2	1	0	1	0	2	0.30	20	0.000004
alkA0-57	9000	23	2600000000	3	6	4	0	1	3	0.30	57	0.000004
alkA0-58	9000	23	2120000000	0	0	0	0	0	0	0.30	0	0.000004
alkA0-59	9000	23	2400000000	0	0	0	2	0	0	0.30	7	0.000004
alkA0-60	9000	46.42	1160000000	1	0	3	0	1	1	0.30	20	0.000008
alkA0-61	9000	46.42	1800000000	0	0	0	0	0	1	0.30	3	0.000005
alkA0-62	9000	46.42	1100000000	1	1	1	1	0	1	0.30	17	0.000008
alkA0-63	9000	46.42	1100000000	0	1	1	2	0		0.25	16	0.000008
alkA0-64	9000	46.42	1240000000	1	0	3	0	0	2	0.30	20	0.000007
alkA0-65	9000	45.25	1580000000	0	0	0	0	0	0	0.30	0	0.000006
alkA0-66	18000	24.92	2420000000	0	2	1	0	0	0	0.30	10	0.000007
alkA0-67	18000	24.92	1460000000	0	0	0	0	1	0	0.30	3	0.00001
alkA0-68	18000	44.33	1340000000	0	0	0	0	1	0	0.30	3	0.00001
alkA0-69	45000	112.37	1000000000	0	0	0	0	1	0	0.30	3	0.00005
alkA0-70	45000	68.5	2230000000	0	3	2	5	2	2	0.30	47	0.00002
alkA0-71	45000	63.5	2020000000	0	0	1	0	0	0	0.30	3	0.00002
alkA0-72	45000	63.5	2310000000	0	0	0	0	0	1	0.30	3	0.00002
alkA0-73	45000	63.5	1380000000	1	0	3	1	1	2	0.30	27	0.00003
alkA0-74	45000	63.5	1030000000	1	0	0	0	0	0	0.30	3	0.00004
alkA0-75	45000	63.5	1700000000	1	0	0	0	0	1	0.30	7	0.00003
alkA0-76	45000	45.45	2140000000	12	12	5	7	3	10	0.30	163	0.00002
alkA0-77	45000	45.45	1550000000	1	0	0	1	1	1	0.30	13	0.00003
alkA0-78	45000	45.45	1850000000	3	0	0	0	0	0	0.30	10	0.00002
alkA0-79	45000	45.45	2140000000	1	0	0	1	0	0	0.30	7	0.00002
alkA0-80	45000	45.45	1830000000	0	0	0	0	1	0	0.30	3	0.00003
alkA0-81	45000	45.45	2180000000	0	0	0	0	1	0	0.30	3	0.00002
alkA0-82	45000	44.65	1330000000	0	0	1	0	1	2	0.30	13	0.00003
alkA0-83	45000	44.65	1310000000	3	1	2	1	5	4	0.30	53	0.00003
alkA0-84	45000	44.65	1400000000	1	11	6	7	0	3	0.30	93	0.00003
alkA0-85	45000	22.3	1660000000	7	3	3	3	3	2	0.30	70	0.00003
alkA0-86	45000	22.3	2210000000	0	1	1	2	0	2	0.30	20	0.00002
alkA0-87	45000	22.3	2040000000	0	1	0	0	1	0	0.30	7	0.00002
alkA0-88	45000	22.3	3000000000	0	0	0	0	0	2	0.30	7	0.00002

alkA0-89	45000	22.3	1940000000	0	1	0	2	0	0	0.30	10	0.00002
alkA0-90	45000	48.03	2150000000	0	3	0	0	3	0	0.30	20	0.00002
alkA0-91	45000	48.03	2090000000	0	0	0	0	0	0	0.30	0	0.00002
alkA0-92	45000	48.03	2120000000	0	0	0	2	2	0	0.30	13	0.00002
alkA0-93	45000	48.03	2050000000	2	4	3	3	2	9	0.30	77	0.00002
alkA0-94	45000	46.42	1520000000	5	7	7	9	9	7	0.30	147	0.00003
alkA0-95	45000	46.42	1950000000	2	2	3	2	0	2	0.30	37	0.00002
alkA0-96	45000	46.42	1300000000	0	0	0	0	0	1	0.30	3	0.00004
alkA0-97	45000	46.42	1960000000	2	3	2	5	3	3	0.30	60	0.00002
alkA0-98	45000	46.42	980000000	1	4	5	2	3	4	0.30	63	0.00005
alkA0-99	45000	50.5	1350000000	3	0	3	0	3	0	0.30	30	0.00003
alkA0-100	45000	50.5	1340000000	2	3	1	3	2	0	0.30	37	0.00003
alkA0-101	45000	50.5	1260000000	0	0	0	0	1	3	0.30	13	0.00004
alkA0-102	45000	50.5	2040000000	1	2	0	0	0	0	0.30	10	0.00002
alkA0-103	45000	50.5	1880000000	1	0	2	1	3	1	0.30	27	0.00002
alkA0-104	45000	50.5	1170000000	3	4	8	12	8	9	0.30	147	0.00004
alkA0-105	45000	23.00	1560000000	3	0	3	0	1	2	0.30	30	0.00003
alkA0-106	45000	47.42	1200000000	1	3	0	1	1	4	0.30	33	0.00004
alkA0-107	45000	47.42	1690000000	3	1	1	2	3	3	0.30	43	0.00003
alkA0-108	45000	47.42	2020000000	0	0	0	0	0	0	0.30	0	0.00002
alkA0-109	45000	47.42	1130000000	9	9	9	12	7	4	0.30	167	0.00004
alkA0-110	45000	47.42	1180000000	0	1	2	1	1	1	0.30	20	0.00004
alkA0-111	45000	49.58	2710000000	3	7	4	6	7	7	0.30	113	0.00002
alkA0-112	45000	49.58	2000000000	3	1	5	4	3	2	0.30	60	0.00002
alkA0-113	45000	49.58	1910000000	0	0	0	0	0	0	0.30	0	0.00002
alkA0-114	45000	49.58	2110000000	0	1	0	1	0	0	0.30	7	0.00002
alkA0-115	45000	49.58	1860000000	0	1	0	1	1	0	0.30	10	0.00002
alkA0-116	45000	49.58	1760000000	0	0	0	3	3	1	0.30	23	0.00003
alkA0-117	9000	25.83	1820000000	0	0	0	1	0	0	0.30	3	0.000005
alkA0-118	9000	25.83	1790000000	0	1	0	0	0	0	0.30	3	0.000005
alkA0-119	9000	25.83	1990000000	0	0	0	0	0	0	0.30	0	0.000005
alkA0-120	9000	25.83	1970000000	0	1	1	4	1	0	0.30	23	0.000005
alkA0-121	9000	21.92	1380000000	0	0	2	0	1	0	0.30	10	0.000007
alkA0-122	9000	21.92	1510000000	1	0	1	0	0	1	0.30	10	0.000006
alkA0-123	9000	21.92	1480000000	0	0	0	0	1	0	0.30	3	0.000006
alkA0-124	9000	21.92	1370000000	1	1	0	1	0	0	0.30	10	0.000007
alkA0-125	9000	21.92	1910000000	0	1	1	1	0	0	0.30	10	0.000005
alkA0-126	9000	21.167	1640000000	0	0	0	0	0	0	0.30	0	0.000006
alkA0-127	9000	21.167	1520000000	0	0	1	0	0	0	0.30	3	0.000006
alkA0-128	9000	21.167	1770000000	0	0	1	1	1	1	0.30	13	0.000005
alkA0-129	9000	21.167	1290000000	1	2	1	1	1	1	0.30	23	0.000007
alkA0-130	9000	21.167	1373400000	0	0	0	0	0	0	0.30	0	0.000007
alkA0-131	9000	18.67	1220000000	1	0	0	0	0	0	0.30	3	0.000007
alkA0-132	9000	18.67	1530000000	0	0	0	0	0	0	0.30	0	0.000006
alkA0-133	9000	18.67	1230000000	2	0	0	0	0	0	0.30	7	0.000007
alkA0-134	9000	20.75	1390000000	0	0	0	0	0	0	0.30	0	0.000007
alkA0-135	9000	20.75	2910000000	0	0	0	0	0	0	0.30	0	0.000003
alkA0-136	9000	20.75	1370000000	0	0	0	0	1	0	0.30	3	0.000007
alkA0-137	9000	20.75	2080000000	4	0	0	0	0	0	0.30	13	0.000004
alkA0-138	9000	21.58	2410000000	2	1	0	0	3	2	0.30	27	0.000004
alkA0-139	9000	21.58	2910000000	1	0	0	0	0	1	0.30	7	0.000003
alkA0-140	9000	21.58	1680000000	1	0	0	0	0	0	0.30	3	0.000005
alkA0-141	9000	21.58	2180000000	0	0	0	0	0	0	0.30	0	0.000004
alkA0-142	9000	21.58	1430000000	0	0	0	2	0	0	0.30	7	0.000006
alkA0-143	9000	19.42	1210000000	0	0	0	0	0	0	0.30	0	0.000007

alkA0-144	9000	19.42	1640000000	2	0	0	0	0	0		0.30	7	0.000006
alkA0-145	9000	19.42	1900000000	0	0	0	1	0	3		0.30	13	0.000005
alkA0-146	9000	19.42	1640000000	1	0	0	0	0	0		0.30	3	0.000006
alkA0-147	9000	19.42	1300000000	2	0	0	0	0	0		0.30	7	0.000007
alkA0-148	9000	23.25	2960000000	0	0	0	0	0	0		0.30	0	0.000003
alkA0-149	9000	23.25	1000000000	1	1	1	0	0	0		0.30	10	0.000009
alkA0-150	9000	23.25	1600000000	1	0	0	0	0	0		0.30	3	0.000006
alkA0-151	9000	23.25	1490000000	1	0	0	0	0	1		0.30	7	0.000006
alkA0-152	9000	44.17	1768666667	0	0	0	0	0	0		0.30	0	0.000005
alkA0-153	9000	44.17	978000000	0	0	0	0	0	0		0.30	0	0.000009
alkA0-154	9000	53	1240500000	1	0	0	0	0	0		0.30	3	0.000007
alkA0-155	9000	45.25	961000000	0	1	1	0	0	0		0.30	7	0.000009
alkA0-156	9000	45.25	1397000000	0	0	2	0	5	2		0.30	30	0.000006
alkA0-157	9000	41.25	1833333333	0	1	0	0	0	0		0.30	3	0.000005
alkA0-158	9000	41.25	1633333333	0	0	0	0	0	0		0.30	0	0.000006
alkA0-159	9000	44.75	2060000000	0	0	0	0	0	0		0.30	0	0.000004
alkA0-160	9000	44.75	1913333333	0	0	0	1	0	0		0.30	3	0.000005
alkA0-161	9000	44.75	2140000000	0	0	1	0	0	0		0.30	3	0.000004
alkA0-162	9000	44.75	2160000000	0	0	0	1	1	0		0.30	7	0.000004
alkA0-163	9000	44.08	1966666667	0	0	0	0	0	0		0.30	0	0.000005
alkA0-164	9000	44.08	1905000000	0	0	0	0	0	0		0.30	0	0.000005
alkA0-165	9000	45	2270000000	0	0	0	0	0	0		0.30	0	0.000004
alkA0-166	9000	45	2150000000	0	0	0	1	0	0		0.30	3	0.000004
alkA0-167	9000	45	2370000000	0	0	0	0	0	0		0.30	0	0.000004
alkA0-168	9000	46	1213333333	0	0	0	0	0	0		0.30	0	0.000007
alkA0-169	9000	46	1817000000	0	0	0	0	0	0		0.30	0	0.000005
alkA0-170	9000	40.28	1316666667	0	0	0	0	0	0	0	0.35	0	0.000007
alkA0-171	9000	48	1400500000	0	0	0	0	0	0		0.30	0	0.000006
alkA0-172	9000	47	1110000000	0	0	2	0	0	0		0.30	7	0.000008
alkA0-173	9000	46.58	998666667	0	0	0	0	0	0		0.30	0	0.000009
alkA0-174	9000	45.67	966666667	0	2	2	2	1	3		0.30	33	0.000009
alkA0-175	9000	45	1314500000	0	0	0	0	0	0		0.30	0	0.000007
alkA0-176	9000	45	980666667	0	0	0	0	0	0		0.30	0	0.000009
alkA0-177	9000	38.92	1513500000	0	0	0	0	0	0		0.30	0	0.000006
alkA0-178	9000	45.17	968666667	0	0	0	0	0	0		0.30	0	0.000009
alkA0-179	9000	46	1780000000	4	4	4	2	3	2		0.30	63	0.000005
alkA0-180	9000	46	1350000000	0	0	0	0	0	1		0.30	3	0.000007
alkA0-181	9000	46.83	1633000000	0	0	0	0	0	0		0.30	0	0.000006
alkA0-182	9000	46.83	1506000000	0	0	0	0	0	0		0.30	0	0.000006
alkA0-183	9000	46.83	1794666667	1	0	0	0	0	0		0.30	3	0.000005
alkA0-184	9000	48	3041333333	1	1	0	0	0	0		0.30	7	0.000003
alkA0-185	9000	48	1748000000	1	0	0	0	0	0		0.30	3	0.000005
alkA0-186	9000	48	1877333333	1	0	0	0	0	0		0.30	3	0.000005
alkA0-187	9000	46	2612000000	1	1	2	0	0	0		0.30	13	0.000003
alkA0-188	9000	46	2521000000	16	13	8	9	12	11		0.30	230	0.000004
alkA0-189	9000	46	1882666667	1	5	0	0	0	0		0.30	20	0.000005
Average	18286	37	1764679365										0.00001
SD	15669	15	504721425										0.00001
Median	9000	42	1768666667										0.000006
Average			1.8×10^9										
SD			0.5×10^9										
Median			1.8×10^9										
												**%**	**μ, × 10^{-9}**
			zeros = 53								**AT→GC**	9.3	0.129

C = 189
p_0 = 0.28
m_{obs} = 1.27
m_{act} = 2.46
μ = 1.39 × 10^{-9}

GC→AT	38	0.533
GC→TA	15	0.210
AT→CG	22	0.307
AT→TA	7.0	0.0970
GC→CG	1.2	0.0161
BS	93	1.29
Indels	2.3	0.0324
Unknown	4.7	0.0646
**Total**	100	1.39

0.1 mM fdU

57 experiments selected from totally 128 experiments

Culture	N_0	Time (h)	N_t	Mutants/plate							z	r	N_0/N_t
				1	2	3	4	5	6	7			
alkA0.1-1	9000	45.08	3070000000	0	3	1	1	0	1		0.30	20	0.000003
alkA0.1-2	9000	45.08	1614000000	4	4	6	5	4	3		0.30	87	0.000006
alkA0.1-3	9000	49.67	1600000000	8	7	8	5	6	7		0.30	137	0.000006
alkA0.1-4	9000	24.08	1306000000	5	5	5	2	0	4		0.30	70	0.000007
alkA0.1-5	9000	24.08	1356000000	1	2	1	1	0	0		0.30	17	0.000007
alkA0.1-6	9000	44.17	1200000000	3	5	2	1	9	2		0.30	73	0.000008
alkA0.1-7	9000	44.17	1540000000	8	7	7	5	10	7		0.30	147	0.000006
alkA0.1-8	9000	44.17	1186000000	10	6	6	5	7	7		0.30	137	0.000008
alkA0.1-9	9000	65.41	1440000000	0	0	0	0	0	1		0.30	3	0.000006
alkA0.1-10	9000	68.41	2560000000	0	0	0	0	2	2		0.30	13	0.000004
alkA0.1-11	9000	65.41	1666000000	0	0	0	0	2	1		0.30	10	0.000005
alkA0.1-12	9000	65.41	1350000000	0	1	0	0	0	0		0.30	3	0.000007
alkA0.1-13	9000	23	2090000000	0	1	0	3	0	0		0.30	13	0.000004
alkA0.1-14	45000	112.37	1100000000	1	1	2	0	0	1		0.30	17	0.00004
alkA0.1-15	45000	112.37	1090000000	0	0	1	0	0	1		0.30	7	0.00004
alkA0.1-16	45000	68.5	2110000000	8	10	1	6	0	3		0.30	93	0.00002
alkA0.1-17	45000	68.5	2990000000	1	0	2	1	1	1		0.30	20	0.00002
alkA0.1-18	45000	68.5	1850000000	4	15	6	3	2	1		0.30	103	0.00002
alkA0.1-19	45000	48.03	2050000000	1	1	0	1	0	1		0.30	13	0.00002
alkA0.1-20	45000	48.03	2550000000	0	0	1	1	0	0		0.30	7	0.00002
alkA0.1-21	45000	48.03	1280000000	3	0	2	2	1	0		0.30	27	0.00004
alkA0.1-22	45000	48.03	1960000000	2	1	0	3	1	1		0.30	27	0.00002
alkA0.1-23	45000	48.03	1790000000	7	3	3	3	2	3		0.30	70	0.00003
alkA0.1-24	45000	23	1160000000	18	23	16	15	10	24		0.30	353	0.00004
alkA0.1-25	45000	47.42	1180000000	2	7	7	4	5	4		0.30	97	0.00004
alkA0.1-26	45000	47.42	1080000000	2	0	3	3	2	1		0.30	37	0.00004
alkA0.1-27	45000	47.42	1320000000	4	2	3	6	1	1		0.30	57	0.00003
alkA0.1-28	45000	47.42	1060000000	0	2	1	3	2	2		0.30	33	0.00004
alkA0.1-29	45000	47.42	1320000000	15	13	10	9	8	9		0.30	213	0.00003
alkA0.1-30	9000	44.17	1383333333	2	0	2	0	2	0		0.30	20	0.000007
alkA0.1-31	9000	44.17	1123333333	0	0	0	0	0	0		0.30	0	0.000008
alkA0.1-32	9000	44.17	1118666667	0	0	0	0	0	0		0.30	0	0.000008
alkA0.1-33	9000	44.17	908250000	1	1	2	0	0	0		0.30	13	0.00001
alkA0.1-34	9000	44.17	1497333333	0	0	2	1	0	2		0.30	17	0.000006
alkA0.1-35	9000	44.17	1198000000	1	0	1	1	0	0		0.30	10	0.000008
alkA0.1-36	9000	53	939680000	1	5	6	4	6	8		0.30	100	0.00001
alkA0.1-37	9000	53	985000000	1	2	5	0	5	2		0.30	50	0.000009
alkA0.1-38	9000	53	1338000000	2	2	1	1	0	2		0.30	27	0.000007
alkA0.1-39	9000	53	1657000000	2	6	5	3	5	7		0.30	93	0.000005

alkA0.1-40	9000	53	1605000000	3	18	12	13	12	15		0.30	243	0.000006
alkA0.1-41	9000	53	1392500000	0	0	2	1	3	2		0.30	27	0.000007
alkA0.1-42	9000	45.5	968500000	0	0	0	0	1	0		0.30	3	0.000009
alkA0.1-43	9000	45.25	1120500000	1	0	1	5	6	4		0.30	57	0.000008
alkA0.1-44	9000	45.25	1332500000	1	2	3	0	8	9		0.30	77	0.000007
alkA0.1-45	9000	45.25	1389000000	0	0	1	2	2	0		0.30	17	0.000007
alkA0.1-46	9000	45.25	1595500000	3	8	8	1	0	6		0.30	87	0.000006
alkA0.1-47	9000	45.25	1813000000	2	7	5	4	3	4		0.30	83	0.000005
alkA0.1-48	9000	45.25	1441000000	3	1	2	3	0	3		0.30	40	0.000006
alkA0.1-49	9000	45.25	3069000000	4	2	5	0	1	1		0.30	43	0.000003
alkA0.1-50	9000	45.25	1191000000	0	2	0	1	0	1		0.30	13	0.000008
alkA0.1-51	9000	41.25	1420000000	0	0	1	0	0	2		0.30	10	0.000006
alkA0.1-52	9000	41.25	1886666667	0	0	0	0	0	0		0.30	0	0.000005
alkA0.1-53	9000	41.25	1720000000	0	0	0	0	0	0		0.30	0	0.000005
alkA0.1-54	9000	41.25	1406666667	0	1	0	0	0	1		0.30	7	0.000006
alkA0.1-55	9000	41.25	1566666667	0	0	0	0	0	0		0.30	0	0.000006
alkA0.1-56	9000	41.25	1466666667	0	0	0	0	0	0		0.30	0	0.000006
alkA0.1-57	9000	41.25	1473333333	0	0	0	0	0	0		0.30	0	0.000006
Average	19105	49	1541650819										0.00001
SD	16320	16	506327265										0.00001
Median	9000	45	1406666667										0.000007
Average			1.5×10^{9}										
SD			0.5×10^{9}										
Median			1.4×10^{9}										

zeros = 7
C = 57
p_0 = 0.123
m_{obs} = 2.10
m_{act} = 4.06
μ = 2.89×10^{-9}

	%	μ, $\times 10^{-9}$
AT→GC	62	1.80
GC→AT	23	0.657
GC→TA	4.6	0.131
AT→CG	0	0
AT→TA	6.1	0.175
GC→CG	0	0
BS	95	2.76
Indels	0	0
Unknown	4.6	0.131
**Total**	100	2.89

0.2 mM fdU

59 experiments selected from totally 101 experiments

**Culture**	N_0	**Time (h)**	N_t	**Mutants/plate**							z	r	N_0/N_t
				**1**	**2**	**3**	**4**	**5**	**6**	**7**			
alkA0.2-1	9000	23	2360000000	0	0	0	0	0	0		0.30	0	0.000004
alkA0.2-2	9000	23	1340000000	16	12	17	17	9	18		0.30	297	0.000007
alkA0.2-3	9000	23	1660000000	0	1	1	1	1	0		0.30	13	0.000005
alkA0.2-4	9000	23	1530000000	1	0	1	0	1	0		0.30	10	0.000006
alkA0.2-5	9000	40	2240000000	3	0	1	0	0	2		0.30	20	0.000004
alkA0.2-6	9000	26.5	1760000000	0	0	0	0	0	0		0.30	0	0.000005
alkA0.2-7	9000	42	1290000000	1	0	0	1	1	0		0.30	10	0.000007
alkA0.2-8	9000	42	1220000000	0	0	1	0	0	1		0.30	7	0.000007
alkA0.2-9	9000	42	980000000	0	0	0	0	0	0		0.30	0	0.000009
alkA0.2-10	9000	42	1270000000	1	1	1	0	2	0		0.30	17	0.000007
alkA0.2-11	9000	42	1070000000	0	0	0	0	0	0		0.30	0	0.000008

alkA0.2-12	9000	23	2500000000	1	1	0	0	0	1	0.30	10	0.000004
alkA0.2-13	9000	23	1710000000	0	0	0	0	0	0	0.30	0	0.000005
alkA0.2-14	9000	23	1260000000	0	0	0	0	0	0	0.30	0	0.000007
alkA0.2-15	9000	23	2430000000	0	1	0	0	0	0	0.30	3	0.000004
alkA0.2-16	9000	46.42	1150000000	0	2	2	3	3		0.25	40	0.000008
alkA0.2-17	45000	50.5	1220000000	2	5	3	3	3	2	0.30	60	0.00004
alkA0.2-18	45000	50.5	910000000	2	1	8	2	0	2	0.30	50	0.00005
alkA0.2-19	45000	50.5	1280000000	0	1	0	0	0	0	0.30	3	0.00004
alkA0.2-20	45000	50.5	1280000000	10	13	9	16	8	11	0.30	223	0.00004
alkA0.2-21	45000	50.5	1040000000	2	1	0	2	1	1	0.30	23	0.00004
alkA0.2-22	45000	50.5	1190000000	2	3	0	0	0	1	0.30	20	0.00004
alkA0.2-23	9000	48	1956000000	2	3	2	3	3	1	0.30	47	0.000005
alkA0.2-24	9000	48	2176800000	0	1	4	1	3	0	0.30	30	0.000004
alkA0.2-25	9000	48	1560000000	1	0	1	2	0	0	0.30	13	0.000006
alkA0.2-26	9000	48	2080000000	2	4	1	1	5	0	0.30	43	0.000004
alkA0.2-27	9000	48	2354000000	0	4	3	3	0	0	0.30	33	0.000004
alkA0.2-28	9000	48	916000000	3	0	0	2	2	1	0.30	27	0.00001
alkA0.2-29	9000	48	2522000000	5	0	0	0	1	1	0.30	23	0.000004
alkA0.2-30	9000	48	996000000	0	1	1	0	0	0	0.30	7	0.000009
alkA0.2-31	9000	48	1188500000	0	0	2	4	1	0	0.30	23	0.000008
alkA0.2-32	9000	48	1259500000	0	0	0	0	0	0	0.30	0	0.000007
alkA0.2-33	9000	47.5	1020500000	0	0	1	0	0	0	0.30	3	0.000009
alkA0.2-34	9000	47.5	1081333333	0	0	0	0	0	0	0.30	0	0.000008
alkA0.2-35	9000	47.5	971500000	0	0	0	0	0	0	0.30	0	0.000009
alkA0.2-36	9000	47	994666667	0	0	0	1	0	0	0.30	3	0.000009
alkA0.2-37	9000	46.58	925333333	0	0	0	0	0	1	0.30	3	0.00001
alkA0.2-38	9000	46	1208000000	1	2	2	2	1	2	0.30	33	0.000008
alkA0.2-39	9000	46	1240000000	0	1	2	0	0	1	0.30	13	0.000007
alkA0.2-40	9000	46	1240000000	1	1	0	1	0	2	0.30	17	0.000007
alkA0.2-41	9000	46	1380000000	1	0	0	0	1	0	0.30	7	0.000007
alkA0.2-42	9000	46	1320000000	0	2	0	1	0	1	0.30	13	0.000007
alkA0.2-43	9000	46	1340000000	1	7	10	6	4	3	0.30	103	0.000007
alkA0.2-44	9000	46	1250000000	1	1	2	0	0	0	0.30	13	0.000007
alkA0.2-45	9000	46	1190000000	0	0	0	0	0	0	0.30	0	0.000008
alkA0.2-46	9000	46	1360000000	2	2	1	1	1	0	0.30	23	0.000007
alkA0.2-47	9000	46	1230000000	0	0	1	0	0	0	0.30	3	0.000007
alkA0.2-48	9000	72	994500000	1	1	1	2	0	0	0.30	17	0.000009
alkA0.2-49	9000	46.83	2620000000	27	12	29	37	12	27	0.30	480	0.000003
alkA0.2-50	9000	46.83	1040000000	3	2	2	0	0	0	0.30	23	0.000009
alkA0.2-51	9000	46	3006400000	7	3	1	4	1	8	0.30	80	0.000003
alkA0.2-52	9000	46	2884000000	2	0	0	0	0	0	0.30	7	0.000003
alkA0.2-53	9000	46	2208000000	6	4	1	6	5	7	0.30	97	0.000004
alkA0.2-54	9000	46	2470000000	9	11	17	14	12	12	0.30	250	0.000004
alkA0.2-55	9000	46	1592000000	3	4	7	2	4	9	0.30	97	0.000006
alkA0.2-56	9000	46	2624000000	1	4	2	9	4	3	0.30	77	0.000003
alkA0.2-57	9000	46	2376000000	1	2	3	0	0	0	0.30	20	0.000004
alkA0.2-58	9000	46	2135000000	4	1	2	0	2	2	0.30	37	0.000004
alkA0.2-59	9000	46	1238000000	1	4	1	4	0	0	0.30	33	0.000007
Average	12661	43	1570136158									0.00001
SD	10974	9	585319998									0.00001
Median	9000	46	1280000000									0.000007
Average			1.6×10^9									
SD			0.6×10^9									
Median			1.3×10^9									

zeros = 10
C = 59
p_0 = 0.169
m_{obs} = 1.77
m_{act} = 3.44
μ = 2.69 × 10^{-9}

	%	μ, × 10^{-9}
AT→GC	55	1.48
GC→AT	15	0.417
GC→TA	17	0.455
AT→CG	1.4	0.0379
AT→TA	1.4	0.0379
GC→CG	0	0
BS	90	2.42
Indels	0	0
Unknown	9.9	0.265
**Total**	100	2.69

xth⁻ nfo⁻

0 mM fdU (spontaneous mutagenesis)

105 experiments selected from totally 153 experiments

Culture	N_0	Time (h)	N_t	Mutants/plate 1	2	3	4	5	6	7	z	r	N_0/N_t
xthnfo0-1	9000	44.28	1630000000	4	5	2	6	0	4		0.30	70	0.000006
xthnfo0-2	9000	44.28	1660000000	1	2	0	1	3	1		0.30	27	0.000005
xthnfo0-3	9000	44	1990000000	5	1	3	4	5	1		0.30	63	0.000005
xthnfo0-4	9000	44	1910000000	21	20	24	13	19	13		0.30	367	0.000005
xthnfo0-5	9000	44	1320000000	13	8	8	16	13	17		0.30	250	0.000007
xthnfo0-6	9000	44	1553400000	3	3	3	10	7	9		0.30	117	0.000006
xthnfo0-7	9000	42.58	2810000000	14	11	10	17	16	21		0.30	297	0.000003
xthnfo0-8	9000	42.58	2920000000	1	3	2	2	1	3		0.30	40	0.000003
xthnfo0-9	9000	68.33	1710000000	0	0	0	1	2	0		0.30	10	0.000005
xthnfo0-10	9000	44.08	2990000000	1	1	3	1	1	3		0.30	33	0.000003
xthnfo0-11	18000	47	1510000000	0	2	0	0	0	0		0.30	7	0.00001
xthnfo0-12	18000	47	960000000	2	3	1	0	0	0		0.30	20	0.00002
xthnfo0-13	9000	26.5	1320000000	0	0	1	0	0	0		0.30	3	0.000007
xthnfo0-14	9000	26.5	1380000000	1	0	0	0	0	0		0.30	3	0.000007
xthnfo0-15	9000	26.5	1950000000	0	0	0	1	0	0		0.30	3	0.000005
xthnfo0-16	9000	26.5	2080000000	0	0	1	1	0	1		0.30	10	0.000004
xthnfo0-17	9000	26.5	2290000000	0	0	0	0	0	0		0.30	0	0.000004
xthnfo0-18	9000	26.5	2530000000	0	0	0	0	0	1		0.30	3	0.000004
xthnfo0-19	9000	26.5	1180000000	26	22	0	1	0	1		0.30	167	0.000008
xthnfo0-20	9000	44	920000000	0	0	0	0	0	0		0.30	0	0.00001
xthnfo0-21	9000	44.25	1058500000	0	0	0	0	0	0		0.30	0	0.000009
xthnfo0-22	9000	44.67	1031500000	0	0	0	0	0	0		0.30	0	0.000009
xthnfo0-23	9000	44.67	968000000	0	0	0	0	0	0		0.30	0	0.000009
xthnfo0-24	18000	45	968000000	15	8	7	6	4	8		0.30	160	0.00002
xthnfo0-25	18000	45	1174000000	2	0	0	0	0	0		0.30	7	0.00002
xthnfo0-26	27000	45	1219000000	0	0	0	0	0	0		0.30	0	0.00002
xthnfo0-27	27000	45	1769000000	1	1	1	1	0	0		0.30	13	0.00002
xthnfo0-28	27000	45	2095200000	0	0	0	0	0	0		0.30	0	0.00001
xthnfo0-29	18000	45	1647600000	0	0	0	0	0	0		0.30	0	0.00001
xthnfo0-30	27000	45	1841500000	1	0	0	0	0	0		0.30	3	0.00002
xthnfo0-31	18000	45	1962800000	0	0	0	0	0	0		0.30	0	0.000009
xthnfo0-32	27000	45	1820000000	1	2	1	0	0	0		0.30	13	0.00002

xthnfo0-33	18000	45	1120000000	1	1	0	0	1	0	0.30	10	0.00002
xthnfo0-34	18000	48	1460000000	0	0	0	0	0	0	0.30	0	0.00001
xthnfo0-35	18000	48	1300000000	1	0	0	0	0		0.25	4	0.00001
xthnfo0-36	18000	48	2133333333	0	0	0	0	0		0.25	0	0.000008
xthnfo0-37	18000	48	2160000000	1	1	0	0	0	0	0.30	7	0.000008
xthnfo0-38	18000	48	1306666667	0	0	0	1	1	0	0.30	7	0.00001
xthnfo0-39	18000	48	2375000000	1	1	0	0	0	0	0.30	7	0.000008
xthnfo0-40	18000	48	2025000000	1	0	0	0	0	0	0.30	3	0.000009
xthnfo0-41	18000	48	2866666667	0	0	0	0	0	0	0.30	0	0.000006
xthnfo0-42	18000	48	2160000000	1	1	0	0	0	0	0.30	7	0.000008
xthnfo0-43	18000	48	2535000000	1	0	0	0	0	0	0.30	3	0.000007
xthnfo0-44	18000	48	2635000000	1	0	0	0	0	0	0.30	3	0.000007
xthnfo0-45	18000	48	2160000000	1	1	0	0	0	0	0.30	7	0.000008
xthnfo0-46	18000	48	2465000000	5	1	4	0	0	1	0.30	37	0.000007
xthnfo0-47	18000	48	2235000000	0	0	0	0	0	0	0.30	0	0.000008
xthnfo0-48	18000	48	2530000000	1	0	0	0	0		0.25	4	0.000007
xthnfo0-49	18000	48	2255000000	14	9	14	9	6	8	0.30	200	0.000008
xthnfo0-50	18000	48	3065000000	1	3	1	2	1	0	0.30	27	0.000006
xthnfo0-51	18000	48	2420000000	1	3	6	7	0	0	0.30	57	0.000007
xthnfo0-52	18000	46.5	2285000000	0	0	0	0	0		0.25	0	0.000008
xthnfo0-53	18000	46.5	2165000000	2	0	4	2	1		0.25	36	0.000008
xthnfo0-54	18000	46.5	1980000000	2	1	2	3	3		0.25	44	0.000009
xthnfo0-55	18000	46.5	2340000000	0	0	0	0	0		0.25	0	0.000008
xthnfo0-56	18000	46.5	2400000000	0	2	0	0	0		0.25	8	0.000008
xthnfo0-57	18000	46.5	2006666667	0	0	2	0	1		0.25	12	0.000009
xthnfo0-58	18000	46.5	1713333333	0	0	0	2	0		0.25	8	0.00001
xthnfo0-59	18000	46.5	1560000000	6	0	2	1	2		0.25	44	0.00001
xthnfo0-60	8600	72	1236400000	0	0	0	0	0	0	0.30	0	0.000007
xthnfo0-61	34450	48	920000000	0	0	0	0	0	0	0.30	0	0.00004
xthnfo0-62	18000	48	1455000000	0	1	1	1	0	0	0.30	10	0.00001
xthnfo0-63	18000	48	1180000000	0	0	0	0	0	1	0.30	3	0.00002
xthnfo0-64	18000	48	1169000000	1	0	1	0	0	0	0.30	7	0.00002
xthnfo0-65	18000	48	1613000000	1	0	0	2	3	0	0.30	20	0.00001
xthnfo0-66	18000	72	1041333333	0	0	0	0	0	0	0.30	0	0.00002
xthnfo0-67	18000	48	1124000000	1	3	3	0	2	0	0.30	30	0.00002
xthnfo0-68	18000	48	1112000000	0	0	0	0	0	0	0.30	0	0.00002
xthnfo0-69	18000	48	1028000000	1	0	0	0	0	1	0.30	7	0.00002
xthnfo0-70	18000	48	1509000000	0	0	0	0	0	0	0.30	0	0.00001
xthnfo0-71	18000	48	1427000000	0	0	0	0	0	0	0.30	0	0.00001
xthnfo0-72	18000	48	1312000000	0	0	0	0	0	0	0.30	0	0.00001
xthnfo0-73	18000	48	1249000000	0	0	0	0	0	0	0.30	0	0.00001
xthnfo0-74	18000	48	1227000000	1	0	1	0	0	0	0.30	7	0.00002
xthnfo0-75	18000	48	2259000000	0	1	0	0	0	0	0.30	3	0.000008
xthnfo0-76	18000	72	1938000000	0	0	0	0	0	1	0.30	3	0.000009
xthnfo0-77	18000	72	1574000000	1	0	0	1	0	0	0.30	7	0.00001
xthnfo0-78	18000	48	1827000000	0	0	0	0	0	0	0.30	0	0.00001
xthnfo0-79	18000	48	1703000000	1	0	0	0	0	0	0.30	3	0.00001
xthnfo0-80	18000	48	2131200000	0	0	0	0	1	0	0.30	3	0.000008
xthnfo0-81	18000	48	2307200000	0	0	1	0	1	1	0.30	10	0.000008
xthnfo0-82	18000	48	1822400000	0	0	0	1	1	0	0.30	7	0.00001
xthnfo0-83	18000	48	2160000000	0	0	0	0	1	0	0.30	3	0.000008
xthnfo0-84	18000	48	1189000000	0	1	3	0	1	0	0.30	17	0.00002
xthnfo0-85	18000	48	1015000000	0	0	0	0	0	0	0.30	0	0.00002
xthnfo0-86	18000	48	1370000000	0	0	0	0	0	0	0.30	0	0.00001

xthnfo0-87	18000	48	971000000	0	0	0	0	1	0		0.30	3	0.00002
xthnfo0-88	18000	48	997500000	0	1	1	3	1	0		0.30	20	0.00002
xthnfo0-89	18000	48	1526000000	1	1	2	3	1	1		0.30	30	0.00001
xthnfo0-90	18000	48	2131000000	0	0	0	0	0	0		0.30	0	0.000008
xthnfo0-91	18000	48	1767000000	1	1	4	0	2	2		0.30	33	0.00001
xthnfo0-92	18000	48	1576000000	0	0	0	0	0	0		0.30	0	0.00001
xthnfo0-93	18000	48	2020000000	100	150	200	250	300	500		0.30	5000	0.000009
xthnfo0-94	18000	48	1272000000	0	0	0	0	0	0		0.30	0	0.00001
xthnfo0-95	18000	48	2376800000	0	3	5	0	1	3		0.30	40	0.000008
xthnfo0-96	18000	48	1550000000	0	1	1	0	1	0		0.30	10	0.00001
xthnfo0-97	18000	48	1417000000	2	3	3	3	4	3		0.30	60	0.00001
xthnfo0-98	18000	48	1169000000	0	0	0	0	0	0		0.30	0	0.00002
xthnfo0-99	18000	48	2521600000	1	1	0	0	0	0		0.30	7	0.000007
xthnfo0-100	18000	48	1907000000	0	1	0	0	0	0		0.30	3	0.000009
xthnfo0-101	18000	48	2351000000	1	0	0	1	1	0		0.30	10	0.000008
xthnfo0-102	18000	48	2680000000	0	8	2	7	2	0		0.30	63	0.000007
xthnfo0-103	18000	48	961333333.3	1	0	0	0	0	0		0.30	3	0.00002
xthnfo0-104	18000	48	1298666667	0	0	0	0	0	0		0.30	0	0.00001
xthnfo0-105	18000	48	1625333333	0	0	0	0	0	0		0.30	0	0.00001
Average	16696	47	1750875556										0.00002
SD	4689	8	553982584										0.00001
Median	18000	48	1703000000										0.00001
Average			1.8×10^9										
SD			0.6×10^9										
Median			1.7×10^9										

zeros = 32

C = 105

$p_0 = 0.305$

$m_{obs} = 1.19$

$m_{act} = 2.30$

$\mu = 1.35 \times 10^{-9}$

	**%**	μ**, × 10^-9**
AT→GC	15	0.202
GC→AT	46	0.625
GC→TA	13	0.181
AT→CG	10	0.141
AT→TA	6.0	0.0806
GC→CG	1.5	0.0201
BS	93	1.25
Indels	1.5	0.0201
Unknown	6.0	0.0806
**Total**	100	1.35

0.1 mM fdU

58 experiments selected from totally 87 experiments

**Culture**	N_0	**Time (h)**	N_t	**Mutants/plate**							z	r	N_0/N_t
				**1**	**2**	**3**	**4**	**5**	**6**	**7**			
xthnfo0.1-1	9000	44.28	1190000000	12	11	13	12	13	7		0.30	227	0.000008
xthnfo0.1-2	9000	44.28	1590000000	1	1	1	0	0	0		0.30	10	0.000006
xthnfo0.1-3	9000	44	1500000000	10	17	21	17	10	6		0.30	270	0.000006
xthnfo0.1-4	9000	44	1880000000	29	23	8	18	29	19		0.30	420	0.000005
xthnfo0.1-5	9000	44	1290000000	2	3	2	10	0	5		0.30	73	0.000007
xthnfo0.1-6	9000	44	1030000000	14	9	9	14	7	10		0.30	210	0.000009
xthnfo0.1-7	9000	42.58	2580000000	0	1	0	1	0	3		0.30	17	0.000004
xthnfo0.1-8	9000	44	1860000000	1	0	0	0	1	0		0.30	7	0.000005
xthnfo0.1-9	9000	44	2250000000	3	1	0	1	1	1		0.30	23	0.000004
xthnfo0.1-10	9000	44.08	1840000000	7	1	8	7	7	9		0.30	130	0.000005
xthnfo0.1-11	9000	44.08	1840000000	4	4	5	1	4	5		0.30	77	0.000005

xthnfo0.1-12	9000	100	1021500000	0	0	0	0	0	0	0.30	0	0.000009
xthnfo0.1-13	18000	47	1490000000	0	0	0	0	0	0	0.30	0	0.00001
xthnfo0.1-14	18000	47	1330000000	0	0	0	0	0	0	0.30	0	0.00001
xthnfo0.1-15	9000	44	974400000	0	1	0	1	0	0	0.30	7	0.000009
xthnfo0.1-16	9000	44	1323200000	1	0	0	2	1	3	0.30	23	0.000007
xthnfo0.1-17	9000	44	1080000000	1	0	0	0	0	2	0.30	10	0.000008
xthnfo0.1-18	9000	44	910400000	0	2	0	1	0	1	0.30	13	0.00001
xthnfo0.1-19	9000	44.25	1037333333	0	0	1	0	0	0	0.30	3	0.000009
xthnfo0.1-20	9000	44.25	1896000000	0	1	0	0	0	0	0.30	3	0.000005
xthnfo0.1-21	9000	44.25	1020800000	1	0	1	0	1	0	0.30	10	0.000009
xthnfo0.1-22	9000	44.25	1850400000	3	8	8	3	6	5	0.30	110	0.000005
xthnfo0.1-23	9000	44.25	922800000	2	4	0	1	1	0	0.30	27	0.00001
xthnfo0.1-24	18000	45	1514400000	1	1	0	0	0	0	0.30	7	0.00001
xthnfo0.1-25	18000	45	1458500000	0	0	0	0	0	0	0.30	0	0.00001
xthnfo0.1-26	27000	45	1430000000	0	0	0	0	0	0	0.30	0	0.00002
xthnfo0.1-27	27000	45	1469000000	0	0	0	0	0	0	0.30	0	0.00002
xthnfo0.1-28	18000	45	1646800000	18	16	21	21	18	13	0.30	357	0.00001
xthnfo0.1-29	18000	45	1739000000	0	0	0	0	0	0	0.30	0	0.00001
xthnfo0.1-30	27000	45	1701200000	1	1	0	0	0	0	0.30	7	0.00002
xthnfo0.1-31	27000	45	1727500000	1	3	2	0	0	0	0.30	20	0.00002
xthnfo0.1-32	18000	72	1631200000	2	0	0	0	4	1	0.30	23	0.00001
xthnfo0.1-33	18000	72	1628000000	2	5	1	1	1	1	0.30	37	0.00001
xthnfo0.1-34	18000	72	1535000000	1	1	0	0	1	0	0.30	10	0.00001
xthnfo0.1-35	18000	48	2367000000	0	1	0	0	0	0	0.30	3	0.000008
xthnfo0.1-36	18000	48	1247200000	0	0	0	0	0	0	0.30	0	0.00001
xthnfo0.1-37	18000	48	1188000000	6	5	7	15	7	15	0.30	183	0.00002
xthnfo0.1-38	18000	48	1042666667	0	0	0	0	0	0	0.30	0	0.00002
xthnfo0.1-39	18000	48	2252800000	2	0	0	0	1	0	0.30	10	0.000008
xthnfo0.1-40	18000	48	1750400000	0	0	0	1	0	0	0.30	3	0.00001
xthnfo0.1-41	18000	48	2037000000	0	0	0	0	1	0	0.30	3	0.000009
xthnfo0.1-42	18000	48	1467000000	0	0	1	0	0	0	0.30	3	0.00001
xthnfo0.1-43	18000	48	1679000000	0	0	1	0	2	3	0.30	20	0.00001
xthnfo0.1-44	18000	48	1468000000	0	1	0	0	0	0	0.30	3	0.00001
xthnfo0.1-45	18000	48	2000000000	0	0	0	0	0	1	0.30	3	0.000009
xthnfo0.1-46	18000	48	1490400000	1	3	0	0	1	2	0.30	23	0.00001
xthnfo0.1-47	18000	48	1380000000	5	1	2	1	3	2	0.30	47	0.00001
xthnfo0.1-48	18000	48	1036000000	0	0	0	0	0	0	0.30	0	0.00002
xthnfo0.1-49	18000	48	1193333333	1	1	1	2	0	0	0.30	17	0.00002
xthnfo0.1-50	18000	48	1569600000	0	0	0	0	1	0	0.30	3	0.00001
xthnfo0.1-51	18000	48	1278400000	0	0	1	0	0	0	0.30	3	0.00001
xthnfo0.1-52	18000	48	1171000000	0	0	0	0	2	0	0.30	7	0.00002
xthnfo0.1-53	18000	48	1873600000	1	1	0	1	1	1	0.30	17	0.00001
xthnfo0.1-54	18000	48	2179000000	1	0	0	0	0	0	0.30	3	0.000008
xthnfo0.1-55	18000	48	1289000000	0	0	0	0	1	1	0.30	7	0.00001
xthnfo0.1-56	18000	48	1336800000	0	0	0	2	2	0	0.30	13	0.00001
xthnfo0.1-57	18000	48	1428800000	0	0	0	0	2	0	0.30	7	0.00001
xthnfo0.1-58	18000	48	1944400000	2	1	0	0	0	1	0.30	13	0.000009
Average	15362	48	1532014368									0.00001
SD	5333	9	388151573									0.000004
Median	18000	47	1490200000									0.00001
Average			1.5×10^9									
SD			0.4×10^9									
Median			1.5×10^9									

zeros = 10

C = 58

p_0 = 0.172

m_{obs} = 1.76

m_{act} = 3.41

μ = 2.29 × 10^{-9}

	%	μ, × 10^{-9}
AT→GC	49	1.11
GC→AT	33	0.752
GC→TA	7.1	0.164
AT→CG	1.4	0.0327
AT→TA	4.3	0.0982
GC→CG	1.4	0.0327
BS	96	2.19
Indels	0	0
Unknown	4.3	0.0982
**Total**	100	2.29

0.2 mM fdU

59 experiments selected from totally 94 experiments

**Culture**	N_0	**Time (h)**	N_t	**Mutants/plate**							z	r	N_0/N_t
				**1**	**2**	**3**	**4**	**5**	**6**	**7**			
xthnfo0.2-1	9000	26.5	1280000000	2	4	1	0	0	1		0.30	27	0.000007
xthnfo0.2-2	9000	44.67	1008500000	0	0	0	1	1	0		0.30	7	0.000009
xthnfo0.2-3	9000	45	1274000000	0	0	0	0	0	0		0.30	0	0.000007
xthnfo0.2-4	18000	45	2715000000	2	1	1	0	0	0		0.30	13	0.000007
xthnfo0.2-5	18000	45	3095000000	1	2	2	0	0	0		0.30	17	0.000006
xthnfo0.2-6	18000	45	2460000000	1	2	4	0	0	0		0.30	23	0.000007
xthnfo0.2-7	18000	45	2785000000	1	1	0	0	0	0		0.30	7	0.000007
xthnfo0.2-8	18000	45	1117000000	0	0	0	0	0	0		0.30	0	0.00002
xthnfo0.2-9	18000	48	2440000000	0	0	0	0	0	0		0.30	0	0.000007
xthnfo0.2-10	18000	48	1420000000	4	5	3	1	0			0.25	52	0.00001
xthnfo0.2-11	18000	48	1430000000	1	1	0	0	0	0		0.30	7	0.00001
xthnfo0.2-12	18000	48	2480000000	1	2	0	1	0	0		0.30	13	0.000007
xthnfo0.2-13	18000	48	2285000000	1	1	0	0	0			0.25	8	0.000008
xthnfo0.2-14	18000	48	2180000000	1	1	1	2	0	0		0.30	17	0.000008
xthnfo0.2-15	18000	48	2595000000	4	3	2	3	7	6		0.30	83	0.000007
xthnfo0.2-16	18000	48	2130000000	2	1	0	0	0	0		0.30	10	0.000009
xthnfo0.2-17	18000	48	2590000000	1	1	1	0	0			0.25	12	0.000007
xthnfo0.2-18	18000	50.5	2220000000	2	3	1	0	2	3		0.30	37	0.000008
xthnfo0.2-19	18000	50.5	2170000000	0	0	0	0	0	1		0.30	3	0.000008
xthnfo0.2-20	18000	50.5	1350000000	0	1	0	0	0	0		0.30	3	0.00001
xthnfo0.2-21	18000	50.5	1490000000	0	0	4	0	0	1		0.30	17	0.00001
xthnfo0.2-22	18000	46.5	1770000000	1	5	1	4	2			0.25	52	0.00001
xthnfo0.2-23	18000	46.5	1580000000	0	0	0	0	0			0.25	0	0.00001
xthnfo0.2-24	18000	46.5	1860000000	0	0	0	2	1			0.25	12	0.00001
xthnfo0.2-25	18000	46.5	1500000000	0	0	1	0	0			0.25	4	0.00001
xthnfo0.2-26	18000	46.5	2190000000	4	0	1	2	0			0.25	28	0.000008
xthnfo0.2-27	18000	46.5	1620000000	2	1	3	4	1			0.25	44	0.00001
xthnfo0.2-28	18000	46.5	1630000000	0	1	1	1	3			0.25	24	0.00001
xthnfo0.2-29	18000	46.5	2090000000	1	1	1	2	2			0.25	28	0.000009
xthnfo0.2-30	18000	46.5	2525000000	1	0	1	3	1			0.25	24	0.000007
xthnfo0.2-31	18000	46.5	1640000000	2	2	2	7	3			0.25	64	0.00001
xthnfo0.2-32	18000	46.5	1940000000	0	1	3	1	1			0.25	24	0.000009
xthnfo0.2-33	18000	46.5	1700000000	0	0	1	0	0			0.25	4	0.00001
xthnfo0.2-34	18000	46.5	1840000000	0	0	0	0	0			0.25	0	0.00001
xthnfo0.2-35	18000	46.5	1650000000	1	0	0	0	0			0.25	4	0.00001
xthnfo0.2-36	18000	46.5	1300000000	2	3	3	1	1			0.25	40	0.00001
xthnfo0.2-37	18000	46.5	1320000000	2	2	0	3	0			0.25	28	0.00001

xthnfo0.2-38	18000	48	1443000000	0	0	0	0	0	0	0.30	0	0.00001
xthnfo0.2-39	18000	48	1292000000	0	0	1	1	0	0	0.30	7	0.00001
xthnfo0.2-40	18000	48	1160000000	2	1	0	2	1	0	0.30	20	0.00002
xthnfo0.2-41	18000	48	1529000000	0	0	0	0	0	0	0.30	0	0.00001
xthnfo0.2-42	18000	48	995000000	0	1	0	2	0	1	0.30	13	0.00002
xthnfo0.2-43	18000	48	1087000000	0	0	1	0	0	0	0.30	3	0.00002
xthnfo0.2-44	18000	48	1199000000	2	2	1	2	3	1	0.30	37	0.00002
xthnfo0.2-45	18000	48	1468000000	0	0	0	0	2	2	0.30	13	0.00001
xthnfo0.2-46	18000	72	1068000000	1	2	2	6	0	0	0.30	37	0.00002
xthnfo0.2-47	18000	48	931000000	0	0	0	0	0	1	0.30	3	0.00002
xthnfo0.2-48	18000	48	917000000	1	0	1	3	1	0	0.30	20	0.00002
xthnfo0.2-49	18000	48	1463000000	0	0	0	1	0	0	0.30	3	0.00001
xthnfo0.2-50	18000	48	1296000000	0	0	0	0	0	0	0.30	0	0.00001
xthnfo0.2-51	18000	48	911000000	0	0	1	0	0	0	0.30	3	0.00002
xthnfo0.2-52	18000	48	1596000000	0	2	0	1	0	1	0.30	13	0.00001
xthnfo0.2-53	18000	48	1758000000	0	1	0	0	1	1	0.30	10	0.00001
xthnfo0.2-54	18000	48	1102000000	1	2	1	2	1	1	0.30	27	0.00002
xthnfo0.2-55	18000	48	1457000000	0	0	0	0	1	0	0.30	3	0.00001
xthnfo0.2-56	18000	48	1009000000	0	1	1	1	2	0	0.30	17	0.00002
xthnfo0.2-57	18000	48	1342000000	0	0	0	0	0	0	0.30	0	0.00001
xthnfo0.2-58	18000	48	989000000	3	2	4	3	4	2	0.30	60	0.00002
xthnfo0.2-59	18000	48	1556500000	1	0	1	1	0	0	0.30	10	0.00001
Average	17542	47	1665050847									0.00001
SD	1994	4	548459698									0.000004
Median	18000	48	1529000000									0.00001
Average			1.7×10^9									
SD			0.5×10^9									
Median			1.5×10^9									

zeros = 9

C = 59

$p_0 = 0.153$

$m_{obs} = 1.88$

$m_{act} = 3.64$

$\mu = 2.38 \times 10^{-9}$

	**%**	**μ, × 10^{-9}**
**AT→GC**	11	0.257
**GC→AT**	45	1.06
**GC→TA**	26	0.611
AT→CG	2.7	0.0643
AT→TA	5.4	0.129
GC→CG	1.4	0.0321
BS	91	2.15
Indels	0	0
Unknown	9.5	0.225
**Total**	100	2.38

Secondary Selection: $N_t = 0.6–0.7 \pm 0.2 \times 10^9$ cells

Wild-type

0 mM fdU (spontaneous mutagenesis)

41 experiments selected from totally 313 experiments

**Culture**	**N_0**	**Time (h)**	**N_t**	**Mutants/plate**	**z**	**r**	**N_0/N_t**

				1	2	3	4	5	6	7			
wt0-1	18000	49.67	590000000	0	1	0	0	0	0		0.3	3	0.00003
wt0-2	18000	49.67	470000000	0	0	1	0	0	2		0.3	10	0.00004
wt0-3	18000	89.75	380000000	1	0	0	0	0	0		0.3	3	0.00005
wt0-4	9000	114.42	780000000	0	0	0	1	0	0		0.3	3	0.00001
wt0-5	9000	40.75	450000000	1	1	2	2	4			0.25	40	0.00002
wt0-6	9000	40.75	690000000	0	0	4	2	3	0		0.3	30	0.00001
wt0-7	9000	40.75	840000000	1	0	0	0	0	0		0.3	3	0.00001
wt0-8	9000	46.5	810000000	1	1	1	0	0	0		0.3	10	0.00001
wt0-9	9000	64.5	610000000	0	0	0	0	0	0		0.3	0	0.00002
wt0-10	9000	64.5	840000000	1	1	0	0	0	0		0.3	7	0.00001
wt0-11	9000	64.5	730000000	0	0	0	0	0	0		0.3	0	0.00001
wt0-12	9000	64.5	660000000	10	26	7	11	25	22		0.3	337	0.00001
wt0-13	45000	22.58	710000000	1	0	1	0	2	1		0.3	17	0.00006
wt0-14	45000	20.92	750000000	0	0	0	0	0			0.25	0	0.00006
wt0-15	45000	22.42	560000000	0	2	0	2	0			0.25	16	0.00008
wt0-16	45000	22.42	450000000	0	1	0	0	0			0.25	4	0.0001
wt0-17	9000	44.5	694000000	1	0	0	2	1	0		0.3	13	0.00001
wt0-18	9000	44.5	526000000	0	0	0	0	0	0		0.3	0	0.00002
wt0-19	9000	44.5	679000000	0	0	0	0	0	0		0.3	0	0.00001
wt0-20	9000	47.5	659000000	0	0	0	0	0	0		0.3	0	0.00001
wt0-21	9000	48	594666667	0	0	0	0	0	0		0.3	0	0.00002
wt0-22	9000	48	840000000	0	0	0	1	0	1		0.3	7	0.00001
wt0-23	9000	48	310000000	0	0	0	0	0	0		0.3	0	0.00003
wt0-24	9000	48	352666667	0	1	0	0	0	0		0.3	3	0.00003
wt0-25	9000	48	464666667	0	0	0	0	0	0		0.3	0	0.00002
wt0-26	9000	44	453333333	0	0	0	0	0	0		0.3	0	0.00002
wt0-27	9000	43	773333333	0	0	0	0	0	0		0.3	0	0.00001
wt0-28	9000	50.83	730000000	2	2	1	2	0	0		0.3	23	0.00001
wt0-29	9000	144	521333333	0	0	0	0	0	0		0.3	0	0.00002
wt0-30	9000	144	590000000	0	0	0	0	0	0		0.3	0	0.00002
wt0-31	9000	48	533500000	1	0	1	0	0	0		0.3	7	0.00002
wt0-32	9000	48	840000000	0	0	0	0	1	0		0.3	3	0.00001
wt0-33	9000	114.5	680000000	0	0	0	0	0	0		0.3	0	0.00001
wt0-34	9000	44.5	660000000	1	3	5	5	4	3		0.3	70	0.00001
wt0-35	9000	44.5	570000000	4	2	4	4	2	2		0.3	60	0.00002
wt0-36	9000	44.5	650000000	0	0	0	0	0	0		0.3	0	0.00001
wt0-37	9000	44.5	550000000	0	0	0	0	0	1		0.3	3	0.00002
wt0-38	9000	69	770000000	0	0	0	0	0	0		0.3	0	0.00001
wt0-39	9000	69	850000000	0	0	0	0	0	0		0.3	0	0.00001
wt0-40	9000	69	760000000	0	0	0	0	0	0		0.3	0	0.00001
wt0-41	9000	46	797500000	3	3	1	2	2	1		0.3	40	0.00001
Average	13171	56	638268293										0.00002
SD	10856	28	146703858										0.00002
Median	9000	48	660000000										0.00001
Average			0.6×10^9										
SD			0.1×10^9										
Median			0.7×10^9										

zeros = 18

C = 41

p_0 = 0.439

m_{obs} = 0.823

m_{act} = 1.60

	**%**	**μ, × 10^{-9}**
**AT→GC**	14	0.338
**GC→AT**	35	0.844
**GC→TA**	14	0.338
**AT→CG**	12	0.281
**AT→TA**	10	0.253

μ = 2.42 × 10^{-9}

GC→CG	3.5	0.0845
BS	88	2.14
Indels	2.3	0.0564
Unknown	9.3	0.225
**Total**	100	2.42

0.1 mM fdU

22 experiments selected from totally 140 experiments

**Culture**	N_0	**Time (h)**	N_t	**Mutants/plate 1**	**2**	**3**	**4**	**5**	**6**	**7**	z	r	N_0/N_t
wt0.1-1	9000	20.28	520000000	0	0	0	0	0	0		0.3	0	0.00002
wt0.1-2	18000	22.17	530000000	1	1	1	0	0	0		0.3	0	0.00003
wt0.1-3	18000	22.17	590000000	0	0	0	0	0	0		0.3	0	0.00003
wt0.1-4	9000	43.25	860000000	0	0	0	0	2	0		0.3	0	0.00001
wt0.1-5	18000	49.67	820000000	1	1	1	0	1	1		0.3	0	0.00002
wt0.1-6	9000	114.42	880000000	0	0	0	0	0	2		0.3	0	0.00001
wt0.1-7	9000	23.5	690000000	0	0	0	0	0	0		0.3	0	0.00001
wt0.1-8	9000	44.75	860000000	2	1	0	0	0	0		0.3	0	0.00001
wt0.1-9	45000	46.58	670000000	6	18	10	24	15	15		0.3	0	0.00007
wt0.1-10	9000	46	356880000	0	0	1	2	2	4		0.3	0	0.00003
wt0.1-11	9000	46	430080000	1	0	1	0	0	1		0.3	0	0.00002
wt0.1-12	9000	46	348760000	1	0	1	0	0	0		0.3	0	0.00003
wt0.1-13	9000	46	322450000	0	2	0	0	6	0		0.3	0	0.00003
wt0.1-14	9000	44.5	542000000	7	5	2	2	2	0		0.3	0	0.00002
wt0.1-15	9000	44.5	688000000	3	5	3	6	1	4		0.3	0	0.00001
wt0.1-16	9000	44.5	642000000	13	1	7	5	6	0		0.3	0	0.00001
wt0.1-17	9000	44.5	623000000	0	0	1	0	0	0		0.3	0	0.00001
wt0.1-18	9000	44.5	556000000	2	6	3	3	0	0		0.3	0	0.00002
wt0.1-19	9000	44.5	403000000	5	4	5	1	3	1		0.3	0	0.00002
wt0.1-20	9000	44.5	588000000	2	1	9	4	0	0		0.3	0	0.00002
wt0.1-21	9000	45.5	563000000	0	0	0	0	0	0		0.3	0	0.00002
wt0.1-22	9000	44	654000000	0	2	0	1	2	2		0.3	0	0.00001
Average	11864	44	597144091										0.00002
SD	8043	18	165173969										0.00001
Median	9000	45	589000000										0.00002
Average			0.6 × 10^9										
SD			0.2 × 10^9										
Median			0.6 × 10^9										

zeros = 4
C = 22
p_0 = 0.182
m_{obs} = 1.70
m_{act} = 3.30
μ = 5.61 × 10^{-9}

	%	μ, × 10^{-9}
AT→GC	66	3.71
GC→AT	12	0.660
GC→TA	4.4	0.247
AT→CG	2.9	0.165
AT→TA	7.4	0.412
GC→CG	0	0
BS	93	5.20
Indels	0	0
Unknown	7.4	0.412
**Total**	100	5.61

0.2 mM fdU

36 experiments selected from totally 131 experiments

Culture	N_0	Time (h)	N_t	Mutants/plate 1	2	3	4	5	6	7	z	r	N_0/N_t
wt0.2-1	9000	48	678500000	0	0	0	0	0	0		0.3	0	0.00001
wt0.2-2	9000	48	479500000	0	1	0	2	1	0		0.3	13	0.00002
wt0.2-3	9000	48	620000000	0	1	3	1	3	0		0.3	27	0.00002
wt0.2-4	9000	48	330000000	0	0	0	0	0	0		0.3	0	0.00003
wt0.2-5	9000	48	355333333	0	0	0	0	0	1		0.3	3	0.00003
wt0.2-6	9000	44	840000000	0	0	0	0	0	0		0.3	0	0.00001
wt0.2-7	9000	44	720000000	0	0	0	1	2	0		0.3	10	0.00001
wt0.2-8	9000	44	461333333	0	1	0	1	0	1		0.3	10	0.00002
wt0.2-9	9000	44	439500000	0	4	2	2	1	1		0.3	33	0.00002
wt0.2-10	9000	44	773333333	0	0	0	0	0	1		0.3	3	0.00001
wt0.2-11	9000	44	697000000	0	2	1	0	0	0		0.3	10	0.00001
wt0.2-12	9000	43	534500000	0	0	0	0	0	0		0.3	0	0.00002
wt0.2-13	9000	43	660000000	0	1	0	0	0	0		0.3	3	0.00001
wt0.2-14	9000	43	403000000	1	3	2	0	2	2		0.3	33	0.00002
wt0.2-15	9000	43	578666667	0	1	0	0	2	1		0.3	13	0.00002
wt0.2-16	9000	43	462000000	0	0	0	0	0	0		0.3	0	0.00002
wt0.2-17	9000	43	610666667	0	0	0	0	0	0		0.3	0	0.00002
wt0.2-18	9000	43	364000000	0	0	0	0	0	0		0.3	0	0.00003
wt0.2-19	9000	43	516000000	0	0	0	0	0	0		0.3	0	0.00002
wt0.2-20	9000	43	662666667	0	0	0	0	0	0		0.3	0	0.00001
wt0.2-21	9000	43	502000000	0	0	0	0	0	0		0.3	0	0.00002
wt0.2-22	9000	43	499333333	0	0	0	0	0	0		0.3	0	0.00002
wt0.2-23	9000	47.5	450000000	0	0	0	0	1	0		0.3	3	0.00002
wt0.2-24	9000	45.17	736666667	0	0	0	0	0	0		0.3	0	0.00001
wt0.2-25	9000	72.5	350000000	0	0	0	0	0	0		0.3	0	0.00003
wt0.2-26	9000	72.5	360000000	0	0	0	0	0	0		0.3	0	0.00003
wt0.2-27	9000	50.83	360000000	1	1	0	0	0	0		0.3	7	0.00003
wt0.2-28	9000	50.83	330000000	0	0	0	0	0	0		0.3	0	0.00003
wt0.2-29	9000	114.5	646666667	1	0	0	0	0	0		0.3	3	0.00001
wt0.2-30	9000	114.5	790000000	0	0	0	0	0	0		0.3	0	0.00001
wt0.2-31	9000	72	586500000	0	0	0	0	0	0		0.3	0	0.00002
wt0.2-32	9000	46	366666667	0	0	0	0	0	0		0.3	0	0.00003
wt0.2-33	9000	46	676666667	0	0	0	0	0	1		0.3	3	0.00001
wt0.2-34	9000	69	570000000	0	0	0	0	0	0		0.3	0	0.00002
wt0.2-35	9000	69	806666667	0	1	0	0	2	1		0.3	13	0.00001
wt0.2-36	9000	46	590000000	1	2	1	3	0	1		0.3	27	0.00002
Average	9000	53	550199074										0.00002
SD	0	18	151940009										0.000005
Median	9000	46	552250000										0.00002
Average			0.6×10^9										
SD			0.2×10^9										
Median			0.6×10^9										

zeros = 19

C = 36

$p_0 = 0.528$

$m_{obs} = 0.639$

$m_{act} = 1.24$

$\mu = 2.24 \times 10^{-9}$

	%	$\mu, \times 10^{-9}$
AT→GC	40	0.844
GC→AT	35	0.714
GC→TA	19	0.390
AT→CG	1.8	0.0325
AT→TA	3.5	0.0650
GC→CG	1.8	0.0325

	%	μ, × 10^{-9}
BS	93	2.08
Indels	1.5	0.0325
Unknown	5.8	0.130
**Total**	100	2.24

alkA $^{-}$

0 mM fdU (spontaneous mutagenesis)

26 experiments selected from totally 258 experiments

**Culture**	N_0	**Time (h)**	N_t	**Mutants/plate** 1	2	3	4	5	6	7	z	r	N_0/N_t
alkA0-1	2250	23.58	780000000	0	1	1	0	1	0		0.3	10	0.000003
alkA0-2	9000	68.41	800000000	0	0	0	0	0	1		0.3	3	0.00001
alkA0-3	9000	26.25	620000000	0	0	0	0	0	0		0.3	0	0.00002
alkA0-4	9000	48.92	800000000	0	0	0	0	0	0		0.3	0	0.00001
alkA0-5	9000	48.92	580000000	0	0	0	1	0	0		0.3	3	0.00002
alkA0-6	45000	112.37	700000000	0	3	0	0	1	0		0.3	13	0.00006
alkA0-7	45000	44.65	850000000	1	0	1	0	0	0		0.3	7	0.00005
alkA0-8	9000	18.67	700000000	0	0	0	0	0	0		0.3	0	0.00001
alkA0-9	9000	18.67	650000000	0	0	0	0	0	0		0.3	0	0.00001
alkA0-10	9000	20.75	480000000	0	0	0	0	0	0		0.3	0	0.00002
alkA0-11	9000	43.5	853000000	0	2	0	0	0	1		0.3	10	0.00001
alkA0-12	9000	45	377500000	0	0	0	0	0	0		0.3	0	0.00002
alkA0-13	9000	45.5	404666667	0	0	0	0	0	0		0.3	0	0.00002
alkA0-14	9000	45.5	401000000	0	0	0	0	0	0		0.3	0	0.00002
alkA0-15	9000	53	847500000	0	0	0	0	0	0		0.3	0	0.00001
alkA0-16	9000	45.5	708500000	0	0	0	0	0	0		0.3	0	0.00001
alkA0-17	9000	48	514000000	0	0	0	0	0	0		0.3	0	0.00002
alkA0-18	9000	47.68	610000000	1	3	6	7	1	1		0.3	63	0.00002
alkA0-19	9000	47.5	458666667	0	0	0	0	0	0		0.3	0	0.00002
alkA0-20	9000	44.5	893000000	0	0	0	0	0	0		0.3	0	0.00001
alkA0-21	9000	44.5	878666667	0	0	0	0	0	0		0.3	0	0.00001
alkA0-22	9000	46.5	576666667	0	0	0	0	0	0		0.3	0	0.00002
alkA0-23	9000	45.67	550666667	0	0	0	0	0	0		0.3	0	0.00002
alkA0-24	9000	45	476000000	2	0	1	3	1	0		0.3	23	0.00002
alkA0-25	9000	46.08	774666667	0	0	0	0	3	0		0.3	10	0.00001
alkA0-26	9000	38.92	560666667	0	0	0	0	0	0		0.3	0	0.00002
Average	11510	45	647891026										0.00002
SD	9947	18	162314824										0.00001
Median	9000	46	635000000										0.00002
Average			0.6 × 10^9										
SD			0.2 × 10^9										
Median			0.6 × 10^9										

zeros = 17

C = 26

p_0 = 0.654

m_{obs} = 0.425

m_{act} = 0.823

μ = 1.30 × 10^{-9}

	**%**	μ, × 10^{-9}
**AT→GC**	9.3	0.121
**GC→AT**	38	0.499
**GC→TA**	15	0.197
**AT→CG**	22	0.287
**AT→TA**	7.0	0.0907
**GC→CG**	1.2	0.0151
BS	93	1.21

Indels	2.3	0.0303
Unknown	4.7	0.0605
**Total**	100	1.30

0.1 mM fdU

23 experiments selected from totally 128 experiments

**Culture**	N_0	**Time (h)**	N_t	**Mutants/plate** 1	**2**	**3**	**4**	**5**	**6**	**7**	z	r	N_0/N_t
alkA0.1-1	9000	65.41	480000000	1	0	0	0	0	0		0.30	3	0.00002
alkA0.1-2	9000	23	670000000	0	0	0	0	0	0		0.30	0	0.00001
alkA0.1-3	45000	68.5	780000000	0	2	0	1	5	3		0.30	37	0.00006
alkA0.1-4	9000	45.5	385300000	0	0	0	0	0	0		0.30	0	0.00002
alkA0.1-5	9000	45.5	560200000	0	0	0	4	0	0		0.30	13	0.00002
alkA0.1-6	9000	45.5	450000000	0	0	0	0	1	0		0.30	3	0.00002
alkA0.1-7	9000	44.17	834000000	0	0	0	0	0	0		0.30	0	0.00001
alkA0.1-8	9000	44.17	339000000	0	0	0	0	0	0		0.30	0	0.00003
alkA0.1-9	9000	44.17	391000000	0	0	1	0	1	0		0.30	7	0.00002
alkA0.1-10	9000	53	581500000	4	6	9	6	11	4		0.30	133	0.00002
alkA0.1-11	9000	53	553500000	9	8	5	4	1			0.25	108	0.00002
alkA0.1-12	9000	45.5	457000000	0	1	0	0	0	0		0.30	3	0.00002
alkA0.1-13	9000	45.5	811000000	1	0	1	2	1	0		0.30	17	0.00001
alkA0.1-14	9000	45.5	316566667	0	0	0	1	0	0		0.30	3	0.00003
alkA0.1-15	9000	45.5	663500000	0	0	3	2	13	0		0.30	60	0.00001
alkA0.1-16	9000	45.5	808500000	0	0	1	1	1	0		0.30	10	0.00001
alkA0.1-17	9000	45.5	615440000	0	1	1	0	0	0		0.30	7	0.00002
alkA0.1-18	9000	45.5	496500000	0	0	0	0	0	0		0.30	0	0.00002
alkA0.1-19	9000	45.5	396120000	0	0	2	0	0	1		0.30	10	0.00002
alkA0.1-20	9000	45.25	534240000	9	2	6	13	10	11		0.30	170	0.00002
alkA0.1-21	9000	45.25	640666667	0	0	2	0	1	0		0.30	10	0.00001
alkA0.1-22	9000	41.25	480000000	0	0	0	0	1	0		0.30	3	0.00002
alkA0.1-23	9000	41.25	760000000	0	0	0	0	0	0		0.30	0	0.00001
Average	10565	46	565392754										0.00002
SD	7507	8	158788198										0.00001
Median	9000	46	553500000										0.00002
Average			0.6×10^9										
SD			0.2×10^9										
Median			0.6×10^9										

zeros = 6

C = 23

$p_0 = 0.261$

$m_{obs} = 1.34$

$m_{act} = 2.60$

$\mu = 4.70 \times 10^{-9}$

	**%**	μ**, × 10**$^{-9}$
**AT→GC**	62	2.92
**GC→AT**	23	1.07
**GC→TA**	4.6	0.214
**AT→CG**	0	0
**AT→TA**	6.1	0.285
**GC→CG**	0	0
BS	95	4.49
Indels	0	0
Unknown	4.6	0.214
**Total**	100	4.70

0.2 mM fdU

33 experiments selected from totally 101 experiments

Culture	N_0	Time (h)	N_t	Mutants/plate 1	2	3	4	5	6	7	*z*	*r*	N_0/N_t
alkA0.2-1	9000	42	800000000	1	1	1	1	0	1		0.3	17	0.00001
alkA0.2-2	9000	46.42	810000000	0	0	0	0	0	0		0.3	0	0.00001
alkA0.2-3	9000	46.42	380000000	0	0	0	0	0	1		0.3	3	0.00002
alkA0.2-4	9000	48	589333333	0	0	0	1	0	0		0.3	3	0.00002
alkA0.2-5	9000	48	868500000	0	0	0	1	1	0		0.3	7	0.00001
alkA0.2-6	9000	47.68	560000000	0	1	1	3	1	0		0.3	20	0.00002
alkA0.2-7	9000	47.68	640000000	0	1	0	0	0	0		0.3	3	0.00001
alkA0.2-8	9000	48	628000000	0	0	1	0	2	0		0.3	10	0.00001
alkA0.2-9	9000	48	591000000	1	3	2	4	10	7		0.3	90	0.00002
alkA0.2-10	9000	47.5	814000000	0	0	0	0	0	0		0.3	0	0.00001
alkA0.2-11	9000	47.5	666000000	0	0	0	0	0	0		0.3	0	0.00001
alkA0.2-12	9000	47.5	678000000	0	2	1	3	4	7		0.3	57	0.00001
alkA0.2-13	9000	47.5	520000000	0	1	0	0	0	0		0.3	3	0.00002
alkA0.2-14	9000	47.5	517200000	0	0	0	0	0	0		0.3	0	0.00002
alkA0.2-15	9000	47	412000000	0	0	0	0	0	0		0.3	0	0.00002
alkA0.2-16	9000	47	535333333	0	0	0	0	0	0		0.3	0	0.00002
alkA0.2-17	9000	47	859333333	0	0	0	0	0	1		0.3	3	0.00001
alkA0.2-18	9000	47	374000000	0	0	0	0	0	0		0.3	0	0.00002
alkA0.2-19	9000	46.58	786500000	0	0	0	0	0	0		0.3	0	0.00001
alkA0.2-20	9000	46.58	599333333	1	0	1	0	2	1		0.3	17	0.00002
alkA0.2-21	9000	46.58	576666667	0	0	0	0	0	0		0.3	0	0.00002
alkA0.2-22	9000	46.58	834000000	0	0	0	0	0	1		0.3	3	0.00001
alkA0.2-23	9000	46.5	861333333	0	1	1	0	0	0		0.3	7	0.00001
alkA0.2-24	9000	46.5	849000000	0	1	0	0	0	0		0.3	3	0.00001
alkA0.2-25	9000	46.5	512000000	0	0	0	0	0	0		0.3	0	0.00002
alkA0.2-26	9000	46.5	489333333	0	0	0	0	0	0		0.3	0	0.00002
alkA0.2-27	9000	72	794666667	27	22	11	17	16	18		0.3	370	0.00001
alkA0.2-28	9000	46.83	780000000	2	1	1	0	0	0		0.3	13	0.00001
alkA0.2-29	9000	46.83	828000000	0	0	0	0	0	0		0.3	0	0.00001
alkA0.2-30	9000	46.83	615000000	3	4	1	1	1	0		0.3	33	0.00002
alkA0.2-31	9000	46.83	720000000	2	1	0	0	0	0		0.3	10	0.00001
alkA0.2-32	9000	46.83	435333333	2	1	0	0	0	0		0.3	10	0.00002
alkA0.2-33	9000	46.83	720000000	5	1	0	0	0	0		0.3	20	0.00001
Average	9000	48	655874747										0.00002
SD	0	4	153285112										0.00000
Median	9000	47	640000000										0.00001
Average			0.7×10^9										
SD			0.2×10^9										
Median			0.6×10^9										

zeros = 12
C = 33
$p_0 = 0.340$
$m_{obs} = 1.01$
$m_{act} = 1.96$
$\mu = 3.06 \times 10^{-9}$

	%	μ, $\times 10^{-9}$
AT→GC	55	1.68
GC→AT	15	0.474
GC→TA	17	0.517
AT→CG	1.4	0.0431
AT→TA	1.4	0.0431
GC→CG	0	0
BS	90	2.76
Indels	0	0
Unknown	9.9	0.302
**Total**	100	3.06
